# Activities and specificities of CRISPR/Cas9 and Cas12a nucleases for targeted mutagenesis in maize

**DOI:** 10.1111/pbi.12982

**Published:** 2018-07-22

**Authors:** Keunsub Lee, Yingxiao Zhang, Benjamin P. Kleinstiver, Jimmy A. Guo, Martin J. Aryee, Jonah Miller, Aimee Malzahn, Scott Zarecor, Carolyn J. Lawrence‐Dill, J. Keith Joung, Yiping Qi, Kan Wang

**Affiliations:** ^1^ Crop Bioengineering Center Iowa State University Ames IA USA; ^2^ Department of Agronomy Iowa State University Ames IA USA; ^3^ Department of Plant Science and Landscape Architecture University of Maryland College Park MD USA; ^4^ Molecular Pathology Unit Center for Cancer Research, and Center for Computational and Integrative Biology Massachusetts General Hospital Charlestown MA USA; ^5^ Department of Pathology Harvard Medical School Boston MA USA; ^6^ Department of Biostatistics Harvard T.H. Chan School of Public Health Boston MA USA; ^7^ Department of Genetics, Development and Cell Biology Iowa State University Ames IA USA; ^8^ Bioinformatics and Computational Biology Program Iowa State University Ames IA USA; ^9^ Institute for Bioscience and Biotechnology Research University of Maryland Rockville MD USA

**Keywords:** Cas12a (Cpf1), CIRCLE‐seq, CRISPR/Cas, genome editing, off‐target, *Zea mays*

## Abstract

CRISPR/Cas9 and Cas12a (Cpf1) nucleases are two of the most powerful genome editing tools in plants. In this work, we compared their activities by targeting maize *glossy2* gene coding region that has overlapping sequences recognized by both nucleases. We introduced constructs carrying SpCas9‐guide RNA (gRNA) and LbCas12a‐CRISPR RNA (crRNA) into maize inbred B104 embryos using *Agrobacterium*‐mediated transformation. On‐target mutation analysis showed that 90%–100% of the Cas9‐edited T0 plants carried indel mutations and 63%–77% of them were homozygous or biallelic mutants. In contrast, 0%–60% of Cas12a‐edited T0 plants had on‐target mutations. We then conducted CIRCLE‐seq analysis to identify genome‐wide potential off‐target sites for Cas9. A total of 18 and 67 potential off‐targets were identified for the two gRNAs, respectively, with an average of five mismatches compared to the target sites. Sequencing analysis of a selected subset of the off‐target sites revealed no detectable level of mutations in the T1 plants, which constitutively express Cas9 nuclease and gRNAs. In conclusion, our results suggest that the CRISPR/Cas9 system used in this study is highly efficient and specific for genome editing in maize, while CRISPR/Cas12a needs further optimization for improved editing efficiency.

## Introduction

The clustered regularly interspaced short palindromic repeats (CRISPR)‐Cas9 system represents the most widely used genome editing platform for targeted genome modifications, including plants (Li *et al*., 2013; Nekrasov *et al*., 2013; Sander and Joung, 2014; Shan *et al*., 2013). For genome editing applications, a CRISPR/Cas9 system consists of two essential components: a Cas9 effector protein, which induces blunt‐end double strand breaks (DSBs), and a single‐guide RNA (sgRNA), which contains an approximately 20nt targeting sequence (Hsu *et al*., 2014; Jinek *et al*., 2012). DSBs are repaired primarily through either nonhomologous end joining (NHEJ) or homology‐directed repair (HDR) pathways (Bibikova *et al*., 2002; Khanna and Jackson, 2001). Loss‐of‐function mutations are generated by short indels introduced during NHEJ‐mediated repair pathway (Bibikova *et al*., 2002), whereas specific sequence modifications can be achieved by HDR pathway in the presence of a proper repair template (Cong *et al*., 2013), albeit at a much lower efficiency (Capecchi, 1989).

The Cas9 nuclease from *Streptococcus pyogenes* (SpCas9) has been the most widely used Cas9 orthologue, and early studies reported the potential for high level off‐target mutations in human cells (Tsai and Joung, 2016). Significant efforts have been made to reduce off‐target activities (Tsai and Joung, 2016). However, the scope of Cas9 off‐target effects in many flowering plants remains less well defined, especially in maize. Cas12a (Cpf1) is a recently identified class two CRISPR/Cas system (Zetsche *et al*., 2015) and has been successfully used for plant genome editing (Endo *et al*., 2016; Kim *et al*., 2017a; Li *et al*., 2018a; Tang *et al*., 2017; Wang *et al*., 2017; Xu *et al*., 2017; Yin *et al*., 2017). Compared to Cas9, Cas12a has distinct features. First, unlike SpCas9, which recognizes G‐rich ‘NGG’ protospacer‐adjacent motifs (PAMs), Cas12a requires a T‐rich ‘TTTV’ PAM (V = A, C, or G). This distinct PAM requirement enables targeting genomic sequences which lack appropriate PAMs for SpCas9, thus expanding the targetable regions in a given genome. Second, Cas12a only needs a CRISPR RNA (crRNA) and does not require a trans‐activating crRNA (tracrRNA). Third, Cas12a can process multiple crRNAs from an array, thus simplifying multiplex gene editing when compared to Cas9 (Zetsche *et al*., 2017). These characteristics make Cas12a a complementary genome editing tool to Cas9 (Mahfouz, 2017; Zaidi *et al*., 2017).

To our knowledge, Cas12a‐mediated mutagenesis in maize has not been reported in peer‐review journals, and no direct comparisons of Cas9 and Cas12a activities in maize have been made to date. In this study, we examined the efficiencies and specificities of Cas9 and Cas12a‐mediated genome editing in maize.

## Results and discussion

### Experimental design and on‐/off‐target potential prediction

**Figure 1:**
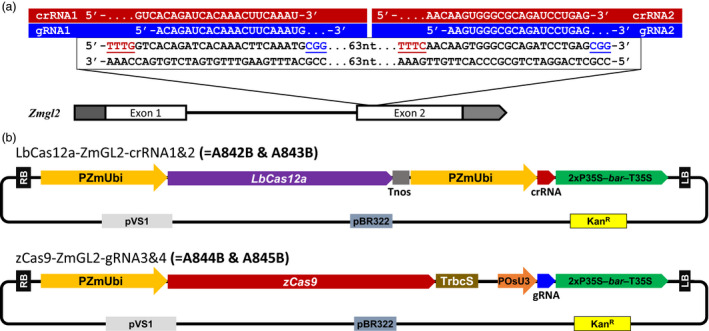
Comparison of Cas9 and Cas12a for genome editing in maize. (a) Cas9 and Cas12a target sequence in maize *glossy2* (*gl2*), which is involved in epicuticular wax deposition in juvenile tissues. PAM sequences (TTTV for Cas12a and NGG for Cas9) were colored and underlined. (b) Schematic representations of the SpCas9 and LbCas12a constructs for *Agrobacterium*‐mediated maize transformation. RB, right border; LB, left border; PZmUbi, *Zea mays* Ubiquitin promoter; Tnos; nopaline synthase terminator; P35S, cauliflower mosaic virus 35S RNA gene promoter; *bar*, bialaphos resistance gene; T35S, cauliflower mosaic virus 35S terminator; TrbcS, *Pisum sativum rbcS E9* terminator, POsU3, *Oryza sativa* U3 small RNA promoter; pVS1, replication origin from *Pseudomonas aeruginosa*; pBR322, replication origin from pMB1; Kan^R^, kanamycin resistance gene.

We chose maize *glossy2* (*gl2*) gene, a gene involved in epicuticular wax formation in juvenile leaves, as the target gene. As illustrated in Figure 1a, we targeted two sites in exon 2 of the *gl2*, where both ‘NGG’ PAM for Cas9 and ‘TTTV’ PAM for Cas12a were present on the two ends of the target site. Thus, at each of these two loci, the target sites of Cas9 and Cas12a are substantially overlapping (Figure 1a), thereby permitting a more direct comparison of Cas9 and Cas12a targeting efficiencies.

**Figure 2:**
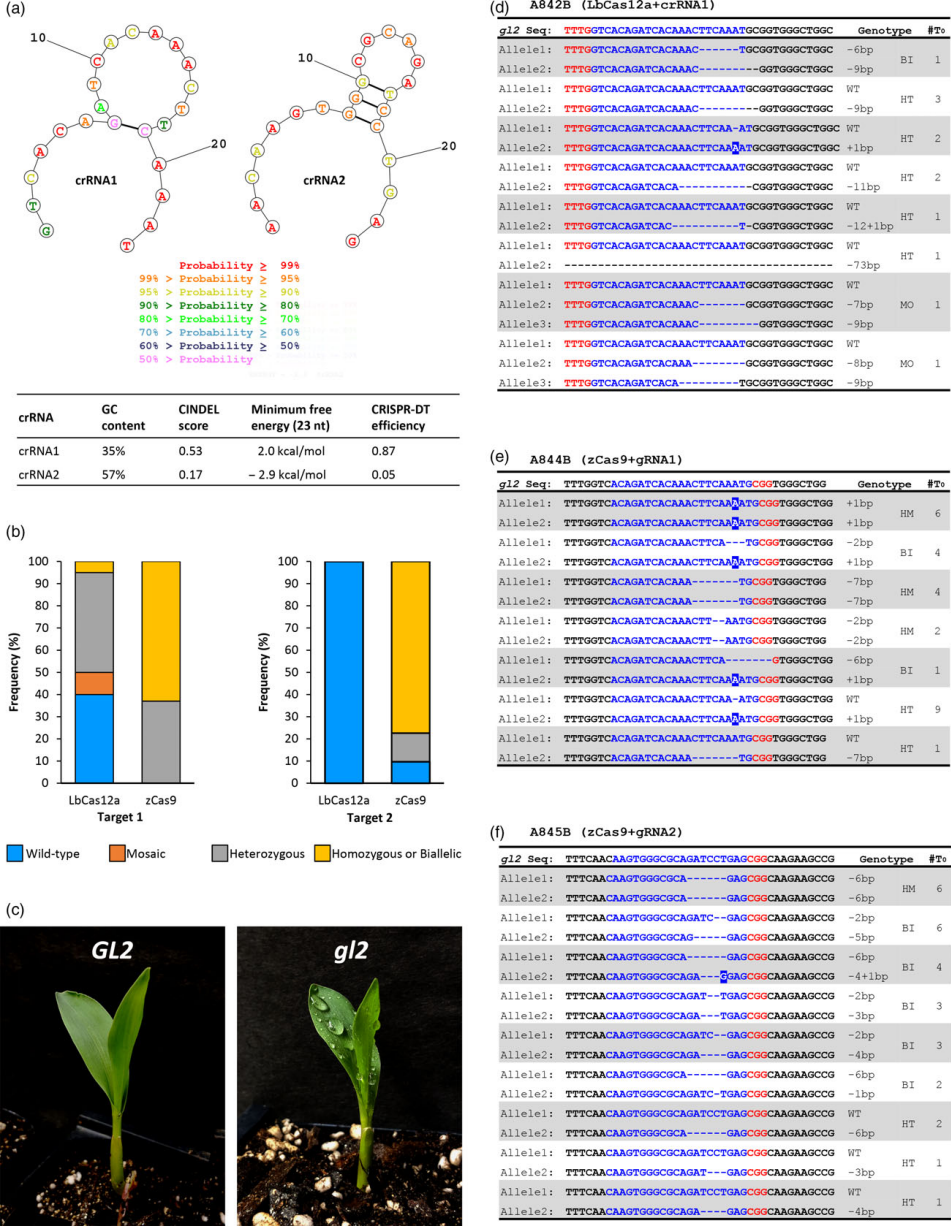
Comparison of on‐target efficiency of Cas12a and Cas9 on two targets. (a) Frequency of T0 genotypes in Cas12a and Cas9 plants at two target sites. (b) The phenotype of *glossy2* (*gl2*) loss‐of‐function of mutant (*gl2,* dull leaf surface retaining water drops) and wild type (*GL2,* glossy leaf surface). (c) Predicted targeting efficiencies and secondary structures of the 23 nt guide sequences of the two Cas12a crRNAs. (d‐f) Indel mutations in T0 transgenic lines. Red letters TTTG and CGG, PAM sequences for Cas12a and Cas9, respectively; blue letters, target sequences in *gl2* exon 2; white letters in blue boxes, insertion mutations; dash lines, indels; #T0, number of T0 mutant lines displays the mutant genotypes. HM, homozygous; BI, biallelic; HT, heterozygous; MO, mosaic.

We then compared on‐ and off‐target potentials of our gRNAs and crRNAs using available web tools. CRISPR Efficiency Predictor programme (Housden *et al*., 2015) predicted that the two Cas9‐gRNAs have 6.5 and 10.3 efficiency scores respectively. gRNAs with scores greater than 7.5 are predicted to have high efficiency in target sequence cleavage. For Cas12a, the two crRNAs were identified and designed manually to overlap the sequences of two gRNAs. Because public bioinformatics tools for Cas12a were not available at the time of design, we were not able to predict their targeting efficacies. However, we retrospectively examined efficiencies of the crRNAs using two recently published web tools, CRISPR AsCpf1 INDEL (CINDEL) score (Kim *et al*., 2017b) and CRISPR DNA Targeting (CRISPR‐DT; Zhu and Liang, 2018). CINDEL score and CRISPR‐DT predicted that crRNA1 has moderate‐high scores, 0.53–0.87 indel frequency, whereas crRNA2 has low scores, 0.05–0.17 indel frequency (Figure 2a). Thus, Cas9 could have a higher efficiency on the second target (6.5 vs. 10.3), whereas Cas12a might have a higher efficiency on the first target (0.53–0.87 vs. 0.05–0.17). Because these tools use different scoring algorithms and the predicted efficiency scores for Cas9 and Cas12a are not directly comparable, we could not predict which Cas system would work better in maize genome editing.

To evaluate off‐target activities of Cas9 and Cas12a in maize, we first examined how specific our intended on‐target sites were relative to the maize genome. To do this, we checked for on‐ and off‐target sites for B73 and B104 using the CRISPR Genome Analysis Tool (CGAT; Brazelton *et al*., 2015), and confirmed that our gRNAs and crRNAs have unique targeting sequence within the maize genome. Next we performed a more in‐depth *in silico* identification of closely related sites (off‐targets) using Cas‐OFFinder (Bae *et al*., 2014). Cas‐OFFinder predicted that Cas12a‐crRNA1 has far fewer sites with six or fewer mismatches in the maize genome than Cas9‐gRNA1: 1193 vs. 19029 (Table S1). Cas12a‐crRNA1 also has only ten sites with four or fewer mismatches, including a gap as a mismatch (Table S1), whereas Cas9‐gRNA1 has 93 such sites. Likewise, Cas12a‐crRNA2 has far fewer off‐target sites compared to Cas9‐gRNA2: 173 versus 71253 (Table S1).

### Constructs for maize transformation

To generate an efficient CRISPR/Cas9 system in maize, we expanded our previous CRISPR/Cas9 toolbox (Lowder *et al*., 2015) with two Gateway compatible vectors: pYPQ166 is an attL1‐attL5 entry vector carrying a codon‐optimized Cas9 for maize expression (Xing *et al*., 2014); and pYPQ210 is an attR1–attR2 destination vector that contains the maize ubiquitin promoter (PZmUbi) for Cas9 expression and bialaphos‐resistance gene (*bar*) for selection of transgenic plants. For CRISPR/Cas12a, we adopted the dual‐Pol II promoter and dual‐ribozyme LbCas12a system that was highly efficient in rice (Tang *et al*., 2017).

As can be seen in Figure 1b, the Cas12a‐crRNA constructs, A842B and A843B, used the maize ubiquitin promoter (PZmUbi) for the expression of both Cas12a and crRNAs, whereas Cas9‐gRNA constructs, A844B and A845B, used PZmUbi for the expression of Cas9 and rice U3 promoter (POsU3) for the expression of gRNAs (Figure 1b). We chose to use PZmUbi for both Cas12a and crRNAs because this combination was proven highly efficient in rice genome editing resulting in 100% targeting efficiency (Tang *et al*., 2017), whereas experiments using POsU3 (Xu *et al*., 2017) or POsU6 (Endo *et al*., 2016) for crRNA expression showed relatively poor targeting efficiency (Tang *et al*., 2017).

The resulting four T‐DNA vectors from the Gateway reactions were delivered into maize immature embryo cells via *Agrobacterium*‐mediated transformation (Frame *et al*., 2011). Four to nine independent bialaphos‐resistant callus events were obtained and 20–31 T0 plantlets were analysed per construct for on‐target mutagenesis (Table 1). Overall, T0 plants did not exhibit any noticeable morphological difference when compared to transgenic plants from other non‐CRISPR projects. They were acclimated in a growth chamber at 26/22 °C (day/night) for about a week before transferred to the greenhouse which has a 16 : 8 photoperiod with day/night temperature of 28/21 °C (Frame *et al*., 2011).

**Table 1:** Percentage of on‐target mutation of T0 maize plants transformed with different CRISPR nucleases and guide RNAs.^a^

Construct ID	CRISPR/Cas Systems	No. of Events	No. of plantlets Analysed	Genotype^b^					Total Efficiency
				Wild Type	Mosaic	Heterozygous	Biallelic	Homozygous	
A842B	LbCas12a + crRNA1	4	20	40% (8)	10% (2)	45% (9)	5% (1)	0% (0)	60% (20)
A843B	LbCas12a + crRNA2	6	23	100% (23)	0% (0)	0% (0)	0% (0)	0% (0)	0% (23)
A844B	zCas9 + gRNA1	7	27	0% (0)	0% (0)	37% (10)	18.5% (5)	44.4% (12)	100% (27)
A845B	zCas9 + gRNA2	9	31	9.7% (3)	0% (0)	12.9% (4)	58.1% (18)	19.4% (6)	90.3% (31)

a Numbers in parentheses represent number of plants analysed.

b Mosaic, three or more mutant sequences in a single plant; Heterozygous, wild‐type sequence and one mutant sequence; Biallelic, two different mutant sequences; Homozygous, one mutant sequence without wild‐type allele.

### On‐target mutagenesis analysis reveals high targeting efficiency of Cas9 in maize

We surveyed the targeting efficiency of Cas9 and Cas12a nucleases in the transgenic maize events. Genomic DNA was isolated from leaf tissues of each T0 plant and a 379 bp region of *gl2* was amplified by PCR using oligonucleotides Zm‐gl2‐exon2‐F1 and R1 (Table S2). Single‐band PCR products were either directly subjected to Sanger sequencing or cloned into pJET1.2 (cloning; ThermoFisher Scientific) first and then performing colony sequencing. Sequencing trace files were analysed by Tracking of Indels by Decomposition (TIDE; Brinkman *et al*., 2014) and DSDecode (Liu *et al*., 2015). Eight individual clones were sequenced for most of the T0 lines (Table S3). T0 and T1 lines were classified into five groups: biallelic, homozygous, heterozygous, mosaic and wild type (Table 1).

On‐target mutagenesis analysis revealed that the Cas9 system tested in this study has a higher efficiency than our Cas12a system for maize genome editing at these two sites (Table 1). Specifically, indel mutation frequency at target 1 was 100% for Cas9 (27/27 T0 plants from seven events) and 60% for Cas12a (12/20 T0 plants from four events; *P *=* *0.0003, two‐tailed *z*‐test). More dramatic contrast was observed on target 2, where Cas9 had a 90% indel mutation frequency (28/31 T0 plants from nine events) and Cas12a showed no indel mutations (0%) in 23 T0 plants from six events (Table 1; *P *=* *0.0001, two‐tailed *z*‐test). These results were consistent with *in silico* predictions which suggested that crRNA2 has a low targeting efficiency (Figure 2a). Importantly, the frequency of biallelic or homozygous T0 mutants was much higher in Cas9‐edited plants, 63%–77.4%, than that in Cas12a‐edited plants, 0%–5% (Figure 2b), indicating better performance of our Cas9 system in maize genome editing at these two sites. Loss‐of‐function mutant phenotypes were observed in T1 plants (Figure 2c), indicating that biallelic or homozygous mutant alleles were either passed down to the next generation or generated by continuing activities of Cas9 and Cas12a in the next generation.

### Profile and inheritance of mutations generated by Cas12a and Cas9

Short indels were the most commonly observed mutations of the target genes in the Cas9‐ and Cas12a‐mutated T0 plants (Figure 2d‐f). Ten out of twelve Cas12a‐crRNA1 plants had 6–73 bp deletions and two plants had 1 bp insertions (Figure 2d). By contrast, 1 bp insertions were the most commonly observed mutations in the Cas9‐gRNA1 plants (20/27; Figure 2e), and all Cas9‐gRNA2 plants (31/31) showed 1–6 bp deletions (Figure 2f). These observations are in line with previously reported mutations found in Cas9‐ and Cas12a‐mutated plants (Kim *et al*., 2017a; Li *et al*., 2018a; Tang *et al*., 2017; Xu *et al*., 2017). For Cas12a‐crRNA1 T0 plants, two of them carried mosaic mutations (Figure 2d and Table 1), which is consistent with its relatively low activity. No mosaic mutation was found in all Cas9 T0 lines (Figure 2e and f; Table 1). Notably, most biallelic or homozygous T0 lines originating from the same immature embryo event have identical mutations (Table 2 and Table S3), suggesting early on‐set of mutagenesis during the transformation process, likely at a single‐cell stage before callus induction.

**Table 2:** Inheritance of on‐target mutations in T1 lines

Event ID	T0^a^	T1‐1	T1‐2	T1‐3	T1‐4	T1‐5	T1‐6	T1‐7
A842B								
1–2	(0, 0)	(0, 0)	(0, 0)	(0, 0)	(0, 0)	(0, 0)	(0, 0)	(0, 0)
1–4	(0, −7, −9)	(0, −9)	(−8, +1)	(0, 0)	(0, 0)	(0, 0)		
2–2	(0, −12 + 1)	(+1, −10)	(+1, −10)	(+1, −10)	(0, −11)	(0, −11)	(0, −11)	(0, −11)
5–1	(0, +1)	(+1, −6)	(+2, −7)	(+1, −8)	(0, −7)	(0, −7)	(0, −9)	(0, −9)
A843B								
2–2	(0, 0)	(0, 0)	(0, 0)	(0, 0)	(0, 0)	(0, 0)	(0, 0)	(0, 0)
3–2	(0, 0)	(0, 0)	(0, 0)	(0, 0)	(0, 0)	(0, 0)	(0, 0)	(0, 0)
A844B								
1–4	(−7, −7)	(−7, +2)	(−7, −6)	(−7, −1)	(0, −7)			
2–5	(+1, +1)	(0, +1)	(0, +1)	(0, +1)				
3–2	(−2, −2)	(−2, −1)	(−5, −2)	(−2, −6)	(−2, −2)	(−2, +1)		
3–4	(−2, −2)	(−2, +1)	(−2, +1)	(−2, +1)	(−2, −2)			
5–2	(+1, −6)	(+1, +1)	(+1, −2)	(0, −6)	(+1, +1)			
6–1	(0, +1)	(−1, −7)	(0, 0)	(−7, −7)				
7–2	(0, +1)	(−1, +1)	(−3, +1)	(+1, +1)	(+1, +1)	(0, +1)		
8–2	(+1, −2)	(−2, −5)	(−1, −4)	(0, −2)	(0, −2)	(0, −2)	(0, +1)	
A845B								
2–4	(−6, −4 + 1)	(0, −3)	(−5, −2)	(0, −3)	(0, −6)			
3–3	(−2, −5)	(−5, −24)	(−5, −5)	(−5, −27)	(−2, −15)	(−2, −24)	(−2, −2)	(−2, −13)
3–4	(−2, −5)	(−5, −1)	(−2, −2)					
4–1	(−2, −4)	(−4, −1)	(−4, −1)	(−4, −5)	(0, −4)			
4–3	(−2, −4)	(−4, −1)	(0, −4)	(−2, −2)	(0, −4)	(−4, +4)		
5–3	(−2, −3)	(−1, −4)	(−3, −9)	(−3, −16)	(0, −3)	(−3, −6)		
6–4	(−6, −6)	(0, −6)	(−1, −6)	(−1, −6)				
7–3	(−1, −6)	(−2, +1)	(−1, −1)	(−1, −5)	(−1, −6)			
8–3	(0, 0)	(−7, −20)	(0, −4)	(0, 0)	(0, 0)			
10–2	(0, −6)	(0, −6)	(0, 0)					

a T0, mutant genotypes in T0 plants; T1‐1, ‐2 to ‐7, sibling T1 plants. Numbers in the parenthesis indicate indel sizes.

Genotyping of T1 progenies produced by crossing T0 lines with wild type B104 pollen donors showed that mutations observed in the maternal parent lines were readily inherited in the next generation (Table 2 and Table S3). However, new mutations were frequently discovered in Cas12a or Cas9 T1 lines (Table 2 and Table S3), consistent with the idea of desired‐target mutator (DTM) by carrying the CRISPR reagents into the next generation (Li *et al*., 2017).

### Low targeting efficiency of Cas12a in maize

The relatively low targeting efficiency of the Cas12a compared to Cas9 used in this study, especially the target 2 construct, prompted us to examine the Cas12a‐crRNA sequences. Cas12a has been successfully applied to plant genome editing, but mutagenesis efficiencies varied greatly depending on crRNA sequences (Kim *et al*., 2017a; Li *et al*., 2018a; Tang *et al*., 2017; Xu *et al*., 2017). As mentioned earlier, both CINDEL score and CRISPR‐DT predicted that crRNA1 has moderate‐high scores, 0.53–0.87 indel frequency, whereas crRNA2 has low scores, 0.05–0.17 indel frequency (Figure 2a). This prediction seems to be consistent with our results.

It has been suggested that crRNAs with a stable secondary structure within the crRNA targeting region are less efficient for gene editing (Kim *et al*., 2017b; Zhu and Liang, 2018). We predicted secondary structures of the two crRNAs using the RNAstructure program (Bellaousov *et al*., 2013) and crRNA2 indeed has a stable stem–loop structure and a lower minimum free energy (MFE) compared to crRNA1, −2.9 versus 2.0 kcal/mol (Figure 2a). Together, these information seems to suggest the possibility that Cas12a crRNAs might be more sensitive than Cas9 gRNAs to structural constraints, such as secondary structures and low MFE, within the guide sequence (Wong *et al*., 2015; Zhu and Liang, 2018). Therefore, one needs to consider not only the sequence specificity of the guide sequence but also possible intramolecular structure when attempting to design highly efficient crRNAs as has been done previously for SpCas9 (Thyme *et al*., 2016).

The poor targeting efficiency of Cas12a in maize could also be attributable to low temperatures (20–28 °C) used for *Agrobacterium*‐mediated maize transformation (Frame *et al*., 2011). Moreno‐Mateos *et al*. (2017) showed that Cas12a activity can be substantially affected by temperature and has a higher activity at 34 °C than at 28 °C. Likewise, Li *et al*. (2018b) also demonstrated temperature‐dependent performance of AsCas12a‐mediated editing in vertebrates. Consistent with these results, we achieved high editing efficiencies with LbCas12a and FnCas12a in rice when higher temperatures (28–30 °C) were applied (Tang *et al*., 2017; Zhong *et al*., 2018). Thus, it may be worth developing a sophisticated high temperature treatment regime for maize immature embryos during transformation to attempt to optimize Cas12a‐mediated genome editing as claimed by Cigan *et al*. (2016) in their patent application.

Another factor is that the Cas9 used in this study was codon‐optimized for maize (Xing *et al*., 2014), while Cas12a was rice codon‐optimized (Tang *et al*., 2017). While both crops are monocotyledonous plants and many promoters/genes have been working effectively in both systems, the codon of Cas nucleases may need to be optimized for specific plant species to maximize its activity.

One limitation in this maize study is its small sample size. Many recent large scale CRISPR system studies were carried out in readily transformable plant species, such as Arabidopsis, rice or tomato (Jacobs *et al*., 2017; Lu *et al*., 2017; Ma *et al*., 2015; Meng *et al*., 2017). Large‐scale analysis comparing efficacies of CRISPR guides and nucleases in recalcitrant crop species such as maize require substantial resources that are not affordable for most academic laboratories. In this study, we used two target sites with overlapping sequences to test Cas9 and Cas12a systems in maize plants. Although the data is limited, the overlapping sequences did allow us to eliminate potential ‘position effect’ of the targets in this comparison. Our Cas9 system appears to be more efficient than our Cas12a system for mutagenizing these two sites, which led to *gl2* mutant phenotype in many T1 individuals.

### Off‐target effects of Cas9 in maize genome editing

We used two approaches to analyse off‐target effects of Cas9 in the transgenic maize lines. We first identified potential off‐target sites using Cas‐OFFinder (Bae *et al*., 2014). This bioinformatics approach allows to identify closely matched off‐sites using simple sequence homology alignment. Our second approach was to employ a recently described Circularization for *In vitro* Reporting of CLeavage Effects by sequencing (CIRCLE‐seq) assay (Tsai *et al*., 2017). This is an *in vitro* biochemical method that can identify a list of closely related sites in the maize genome with Cas9 cleavage propensities. The *in vitro* off‐target list generated by CIRCLE‐seq was then used as a road map to assess the *in planta* off‐sites in the transgenic mutant maize lines.

One important challenge for maize off‐site analysis is that about 85% of the maize genome consists of highly repetitive sequences (Schnable *et al*., 2009). The vast majority of the closely matched sites found by Cas‐OFFinder were within highly repetitive regions. This feature presents challenges when designing primers specific for sequencing the off‐site regions. Specific primers could be designed for only one, two and five off‐targets with four or fewer mismatches for Cas12a‐crRNA1, Cas9‐gRNA1 and gRNA2 respectively (Table S4).

**Figure 3:**
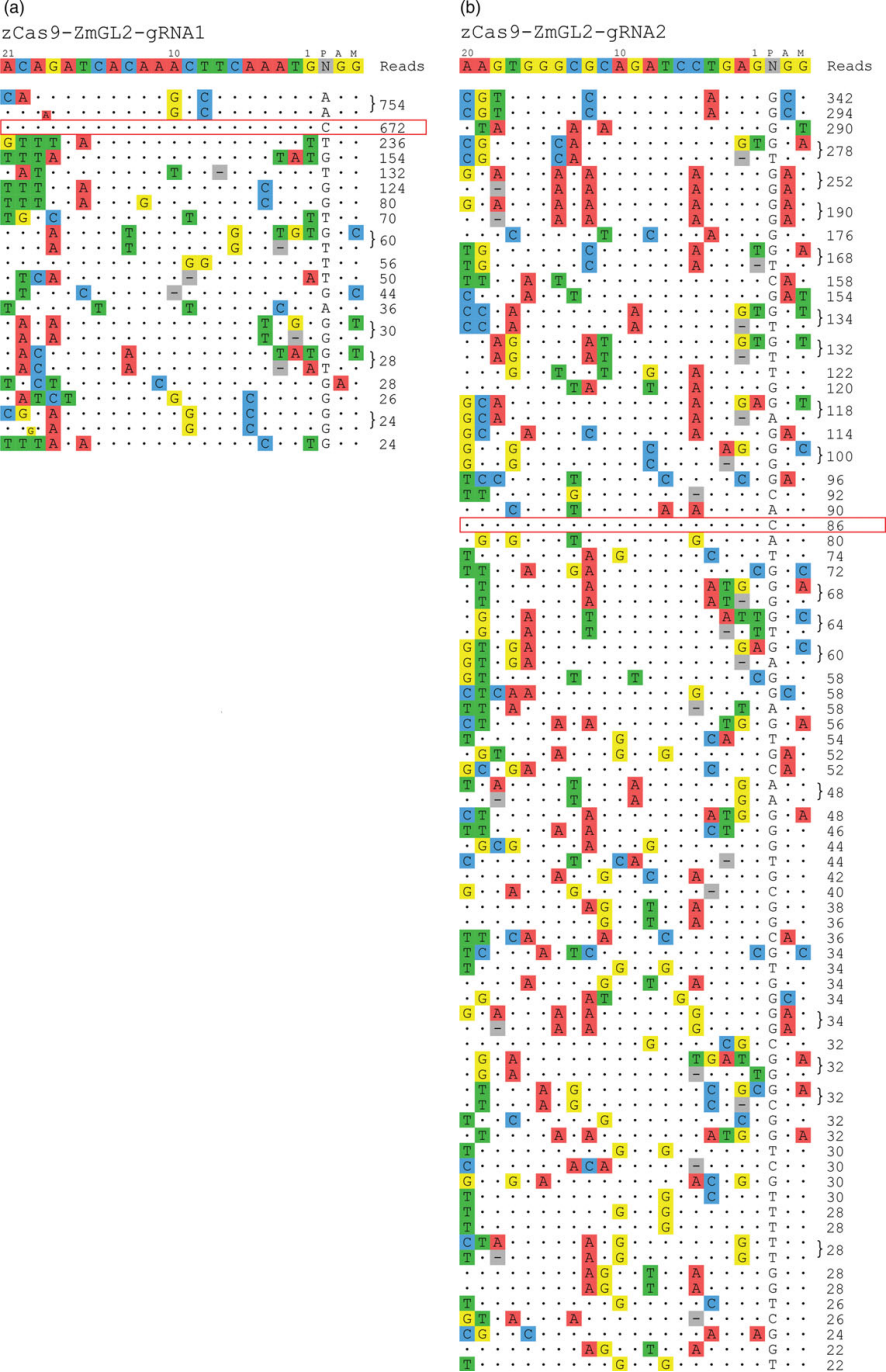
Candidate off‐targets identified by CIRCLE‐seq. (a) and (b) CIRCLE‐seq identified off‐targets for Cas9‐gRNA1 and gRNA2 respectively. Numbers on the right indicate the number of sequence reads mapped to the off‐targets. Targets are highlighted with a box.

We chose to focus on Cas9 and not Cas12a because of the smaller number of closely related sites in the maize genome for Cas12a nucleases as well as their higher genome‐wide specificities reported in human cells (Gao *et al*., 2017; Kim *et al*., 2016; Kleinstiver *et al*., 2016). CIRCLE‐seq analysis identified 18 and 67 off‐target sites cleaved *in vitro* by Cas9‐gRNA1 and Cas9‐gRNA2 respectively (Figure 3a and b; Table S4).

**Figure 4:**
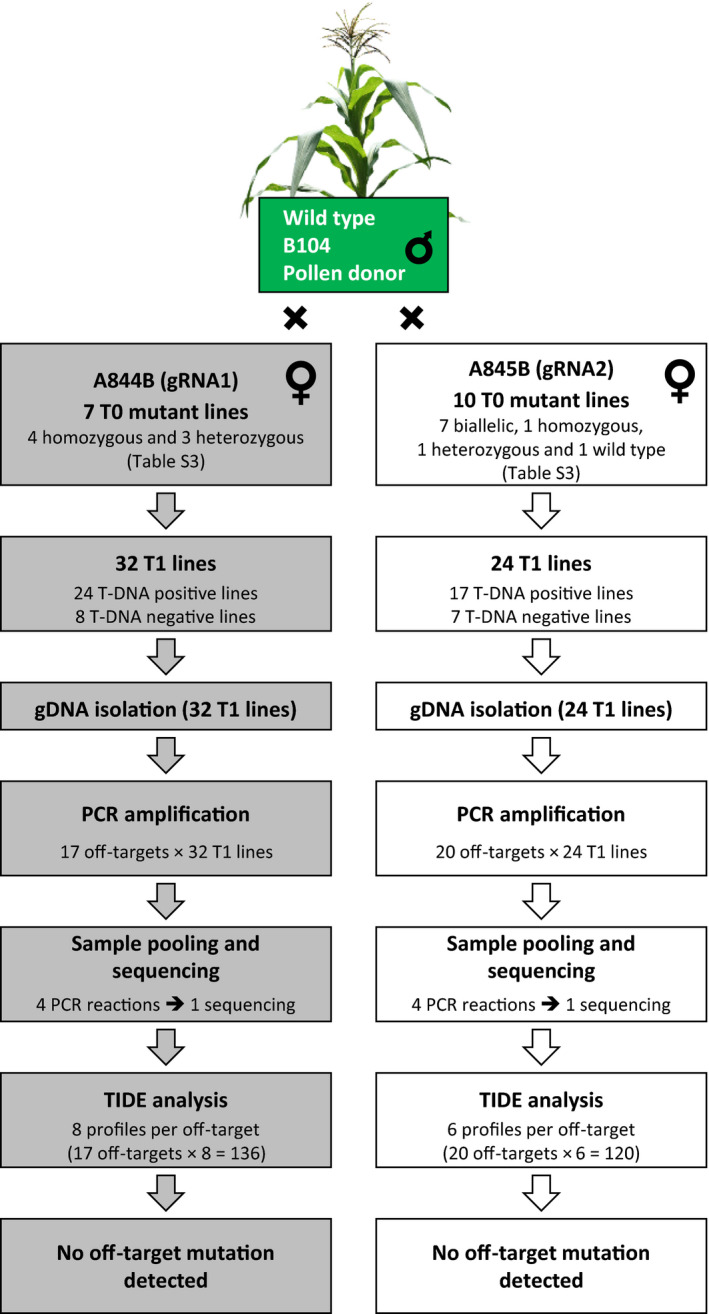
Detection of off‐target mutations. The overall procedure to detect Cas9‐induced off‐target mutations is illustrated. Transgenic T1 lines were obtained by backcrossing T0 plants with wild type pollen donors. See Data S1 for detail information.

We used TIDE (Brinkman *et al*., 2014) to examine whether the off‐target sites identified *in vitro* by CIRCLE‐seq would show mutations *in planta* in T1 maize plants originated from T0 lines bearing on‐target mutations (Figure 4; Table S3). We were unable to assess all of these CIRCLE‐seq off‐targets *in planta* because some of them are present within repetitive regions of the genome. Nevertheless, we were able to design PCR primers to amplify 15 of 18 gRNA1 off‐targets and 18 of 67 gRNA2 off‐targets (Table S4). In addition, we also examined two closely matched sites identified by Cas‐OFFinder for each gRNA (Table S4), thus a total of 17 and 20 off‐targets were examined for gRNA1 and gRNA2 respectively. We screened 32 A844B T1 lines originated from seven T0 lines and 24 A845B T1 lines originated from ten T0 lines (Figure 4; Table S2). Eight A844B and seven A845B T1 lines were T‐DNA negative segregants (Figure 4; Data S1). Most T‐DNA positive T1 lines (21 of 24 A844B and 12 of 17 A845B T1 lines) exhibited *gl2* null mutant phenotypes suggesting inherited Cas9 activities, whereas most T‐DNA negative lines (5 of 8 A844B and 5 of 7 A845B T1 lines) had wild type‐like phenotype for the *gl2* gene (Data S1). TIDE analysis revealed that none of the tested off‐target sites carried a detectable level of indel mutations (Figure 4; Data S1), suggesting no germ‐line–transmittable mutations at these potential off‐target sites.

The lack of detectable level of off‐target mutations in our study suggests a high specificity of our Cas9‐gRNAs in maize genome editing at the tested two sites. However, it might also be attributed to other factors such as sensitivity of the detection method, discrepancy between the *in vitro* and *in vivo* cleavage by Cas9 endonuclease, and incomplete coverage of potential off‐sites due to inability of assessing sequences in the highly repetitive regions. First, TIDE can detect indels with a frequency down to approximately 2% (Brinkman *et al*., 2014), which is sufficient to detect off‐target mutations inherited from T0 lines or new mutations that had occurred at an early stage during embryogenesis in T1 lines. More sensitive mutation detection methods, such as targeted deep sequencing (Tsai *et al*., 2017) using the next generation sequencing technology, would be able to detect rare off‐target mutations. Second, unlike the *in vitro* cleavage reactions which involve naked genomic DNA and a high concentration of Cas9‐gRNA complexes, *in vivo* cleavages are likely affected by numerous cellular factors. In addition, NHEJ repair pathway is relatively accurate (Bétermier *et al*., 2014), thus only a small fraction of DSBs result in indel mutations, further reducing the frequency of off‐target mutations *in vivo*. Lastly, we were unable to test 3 of 18 and 49 of 67 off‐targets identified by CIRCLE‐seq for the two gRNAs, respectively, due to their presence within the repetitive sequences. It is possible that some of the off‐targets within the repetitive sequences might carry mutations, especially for the gRNA2. Nonetheless, recent Cas9‐mediated plant genome editing studies suggest high specificity of Cas9 endonuclease (Feng *et al*., 2018; Tang *et al*., 2018). In general, low frequency somatic null mutations would not be as problematic in plants as they can be in animals, because the chance of passing such somatic mutations into the next generation is low and undesirable mutations in plants can be segregated through breeding in the following generations.

## Conclusion

In summary, we report a successful LbCas12a‐based genome mutagenesis in maize and provide a comparison of the efficiencies and specificities of Cas9 and Cas12a systems in maize. Both nucleases were able to introduce targeted indel mutations in the two sites of the *gl2* gene and generate *gl2* mutant phenotype within one generation, suggesting the utility of both CRISPR/Cas systems for maize genome editing. Compared to previously published Cas9 systems in maize (Char *et al*., 2017; Feng *et al*., 2016, 2018; Li *et al*., 2017; Svitashev *et al*., 2015; Zhu *et al*., 2016), our Cas9 system displayed very high editing efficiency (90%–100%), suggesting this system has promising applications for genome editing in maize. Importantly, it can be easily applied for multiplexed genome editing in maize as it is fully compatible with our multiplex CRISPR/Cas9 assembly toolbox (Lowder *et al*., 2015). Compared to our tested Cas9 system, Cas12a exhibited lower efficiency at the two target sites. Presence of a stable secondary structure in the crRNA2 might explain the lack of activity with Cas12a in our maize experiments (Kim *et al*., 2017b; Zhu and Liang, 2018), suggesting that it may be important to avoid potential secondary structures in the targeting region when designing highly efficient crRNAs for Cas12a. We also performed an analysis of the specificities of Cas9 nucleases in maize and could not detect any off‐target mutations passed to T1 progenies at potential off‐target sites we examined. Overall, our results demonstrate the potential utility of both Cas9 and Cas12a for performing efficient and specific genome editing in maize.

## Experimental procedures

### CRISPR/Cas9 and Cas12a vector construction

#### Construction of the destination vector pYPQ210

To generate the destination vector pYPQ210 (Addgene plasmid #109329) with PZmUbi promoter to drive Cas12a or Cas9 expression, the PZmUbi region was cut off from pYPQ203 (Addgene plasmid #86207) with HindIII and AscI and cloned into pMDC123 at the same sites (Curtis and Grossniklaus, 2003).

#### Construction of the zCas9 entry clone pYPQ166

Two oligonucleotides LNK‐165‐F‐NEW 5′‐CATGGGGTACCCAATTGTTCCGGAACTCTAGATAAGCTTA‐3′ and LNK‐165‐R‐NEW 5′‐CCGGTAAGCTTATCTAGAGTTCCGGAACAATTGGGTACCC‐3′ were phosphorylated using T4 Polynucleotide Kinase, annealed and ligated into pYPQ167 (Addgene plasmid #69329) to replace the Cas9p cassette at *Nco*I and *BspE*I sites, resulting in vector pYPQ167‐LNK‐165. Fragment pEGG‐zCas9‐rbS‐E9t was cut off from pHEE401E (Addgene plasmid #71287) at *Nco*I and *Mfe*I sites and cloned into pYPQ167‐LNK‐165 to generate pYPQ165 (Addgene plasmid #109327). zCas9 was PCR amplified with primers zCas9‐F‐NcoI 5′‐TGGACCATGGATTACAAGGACCACGACGGGGATTA‐3′ and zCas9‐R‐SacI 5′‐GTCGAAACCGATGATACGAACGAA‐3′ and cloned into pYPQ165 at *Nco*I and *Sac*I sites to generate the zCas9 entry clone pYPQ166 (Addgene plasmid #109328).

#### Construction of T‐DNA vectors for CRISPR/Cas12a

Vectors for CRISPR/Cas12a were constructed based on the methods described previously (Tang *et al*., 2017). To achieve maize genome editing using CRISPR/Cas12a, two crRNAs were designed to target the second exon of maize *glossy2* gene (*gl2*). crRNA1 was synthesized as duplexed primers ZmGL2‐crRNA1‐F 5′‐TAGATGTCACAGATCACAAACTTCAAAT‐3′ and ZmGL2‐crRNA1‐R 5′‐GGCCATTTGAAGTTTGTGATCTGTGACA‐3′; while crRNA2 was synthesized as duplexed primers ZmGL2‐crRNA2‐F 5′‐TAGATAACAAGTGGGCGCAGATCCTGAG‐3′ and ZmGL2‐crRNA2‐R 5′‐GGCCCTCAGGATCTGCGCCCACTTGTTA‐3′. Primers were phosphorylated, annealed and ligated into pYPQ141‐ZmUbi‐RZ‐Lb (Addgene plasmid #86197) at its *BsmB*I site. These two vectors, along with the LbCas12a entry clone pYPQ230 (Addgene plasmid #86210), and a destination vector pYPQ210, were assembled using a three‐way Gateway cloning system to make two final T‐DNA vectors (Earley *et al*., 2006).

#### Construction of T‐DNA vectors for CRISPR/Cas9

Vectors for CRISPR/Cas9 were constructed based on protocols described previously (Lowder *et al*., 2015). Similarly, two single gRNAs were designed to target the second exon of *gl2*, with the targeting sites overlapping with the Cas12a targeting sites. gRNA1 was synthesized as duplexed primers ZmGL2‐gR3‐F 5′‐TGGCACAGATCACAAACTTCAAATG‐3′ and ZmGL2‐gR3‐R 5′‐AAACCATTTGAAGTTTGTGATCTGT‐3′; while gRNA2 was synthesized as duplexed primers ZmGL2‐gR4‐F 5′‐TGGCAAGTGGGCGCAGATCCTGAG‐3′ and ZmGL2‐gR4‐R 5′‐AAACCTCAGGATCTGCGCCCACTT‐3′. Primers were phosphorylated, annealed and ligated into pYPQ141D (Addgene plasmid #69293) at its *BsmB*I site. These two vectors, along with the zCas9 (maize codon optimized Cas9) entry clone pYPQ166, and a destination vector pYPQ210, were assembled using the Gateway cloning system to make the final T‐DNA vectors. These vectors were used for *Agrobacterium*‐mediated transformation for maize genome editing.

#### Agrobacterium‐mediated maize transformation

Maize inbred line B104 was transformed using the *Agrobacterium*‐mediated protocol (Frame *et al*., 2011) by Iowa State University Plant Transformation Facility. Bialaphos‐resistant T0 plantlets were grown to maturity and crossed with wild‐type B104 as pollen donors.

### Genome‐wide off‐target identification

#### Cas‐OFFinder prediction

Genome‐wide off‐targets were predicted using the Cas‐OFFinder webtool (http://www.rgenome.net/cas-offinder/; Bae *et al*., 2014) and the Circularization for *In vitro* Reporting of CLeavage Effects by sequencing (CIRCLE‐seq) assay (Tsai *et al*., 2017). Closely matched genomic sites for Cas12a‐crRNAs were predicted using ‘TTTN’ PAM for LbCas12a and crRNA1 (5′‐GTCACAGATCACAAACTTCAAAT‐3′) and crRNA2 (5′‐AACAAGTGGGCGCAGATCCTGAG‐3′) sequences targeting *Zea mays* inbred line B73 (AGPv3). Likewise, Cas9‐gRNA1–2 off‐targets were predicted using “NGG’ PAM for SpCas9 and gRNA1 (5′‐ACAGATCACAAACTTCAAATG‐3′) and gRNA2 (5′‐AAGTTTCGCAGATCCTGAG‐3′) sequences. Cas‐OFFinder predicted off‐targets with four or fewer mismatches were downloaded and highly redundant off‐targets were removed because they were present within highly repetitive regions. Remaining off‐targets were BLAST searched against the B104 genome sequence (Manchanda *et al*., 2017) in MaizeGDB, the maize genetics and genomics database (www.maizegdb.org; Andorf *et al*., 2016), and approximately 1 kb genomic DNA sequences including an off‐target site were retrieved for PCR primer design using Primer3 (Untergasser *et al*., 2012).

#### CIRCLE‐seq analysis

CIRCLE‐seq experiments were performed as previously described (Tsai *et al*., 2017) with minor modifications. All enzymatic steps were purified using paramagnetic beads prepared similar to as previously described (Rohland and Reich, 2012) [GE Healthcare Sera‐Mag SpeedBeads (Fisher Scientific) washed in 0.1x TE and suspended in 20% PEG‐8000 (w/v), 1.5 m NaCl, 10 mm Tris‐HCl pH 8, 1 mm EDTA pH 8 and 0.05% Tween20]. Briefly, for each CIRCLE‐seq reaction, 25 μg of Maize B104 genomic DNA (gDNA) was sheared using an E220evolution (Covaris) to an average length of 300 bp (temperature, 6–7 °C; peak power, 140; duty factor, 10; cycles/burst, 200; duration, 80 s). The sheared gDNA was purified with beads and 3 μg was subject to an end‐repair, A‐tailing and adapter ligation protocol for the addition of uracil‐containing stem–loop DNA adaptors (oSQT1288: 5′‐P‐CGGTGGACCGATGATCUATCGGTCCACCG*T‐3′, where * indicates a phosphorothioate linkage) using the KAPA HTP Library Preparation (no amp) Kit (KAPA BioSystems). Adaptor‐ligated DNA was treated with a mixture of Lambda Exonuclease (NEB) and *E. coli* Exonuclease I (NEB), and then subsequently with a mixture of USER enzyme (NEB) and T4 polynucleotide kinase (NEB). DNA was circularized at 5 ng/μL in 100 μL with T4 DNA ligase, and subsequently treated with 50 U Plasmid‐Safe ATP‐dependent DNase (Epicentre) to degrade remaining linear DNA molecules. *In vitro* cleavage reactions were then performed in 100 μL volumes with 1x Cas9 Nuclease Reaction Buffer (NEB), 90 nm SpCas9 protein (NEB), 90 nm *in vitro* transcribed sgRNA, and 250 ng of Plasmid‐Safe‐treated circularized DNA at 37 °C for 60 min. Linearized products were A‐tailed and ligated (Kapa BioSystems) with NEBNext Illumina hairpin adaptors (NEB), treated with USER enzyme (NEB), PCR amplified using KAPA HiFi polymerase (KAPA Biosystems) with Dual Index NEBNext Multiplex Primers (set 1; NEB). Final libraries were quantified by droplet digital PCR (Bio‐Rad) and sequenced with 150 bp paired‐end reads on an Illumina MiSeq instrument.

Data analysis was performed using v1.1 of the CIRCLE‐seq analysis pipeline using standard parameters (https://github.com/tsailabSJ/circleseq). The analysis manifest file was constructed with the following parameters: reference_genome, Zm‐B104‐DRAFT‐ISU_USDA‐0.2.fa; window_size, 3; mapq_threshold, 50; start_threshold, 1; gap_threshold, 3; mismatch_threshold, 7; merged_analysis, False. The resulting CIRCLE‐seq data is summarized in Figure 3 and Table S4.

### On‐ and off‐target mutagenesis analysis

Total genomic DNA was isolated from leaf tissues of T0 and T1 maize plants as previously described (Allen *et al*., 2006). Oligonucleotides for PCR were designed using Primer3 (Untergasser *et al*., 2012) and primer sequence specificity was checked in MaizeGDB. Primer sequences used for PCR are listed in Table S1. PCR was carried for each locus including a target or off‐target with 50–100 ng of gDNA as template using Phusion high‐fidelity DNA polymerase (ThermoFisher Scientific). A typical PCR reaction contained 1x Phusion high‐fidelity buffer, 125 μm dNTPs, 0.5 μm primers and 0.4 U of Phusion DNA polymerase. PCR reactions were carried out in a thermocycler with the following cycling conditions: 30 s at 98 °C for initial denaturation followed by 35 cycles of [10 s at 98 °C, 20 s at 63 °C, 20 s at 72 °C], and 5 min at 72 °C for final extension. Single‐band amplification was verified by agarose gel electrophoresis. PCR product was cloned into a cloning vector, pJET1.2 (ThermoFisher Scientific), according to the manufacture's instruction or directly subjected to Sanger Sequencing after clean up by ExoSAP‐IT (ThermoFisher Scientific) treatment. The sequence trace files were analysed using the Tracking of Indels by Decomposition (TIDE; Brinkman *et al*., 2014) and DSDecode (Liu *et al*., 2015) with the default parameters.

## Competing interests

J.K.J. has financial interests in Beam Therapeutics, Editas Medicine, Monitor Biotechnologies, Pairwise Plants, Poseida Therapeutics and Transposagen Biopharmaceuticals. J.K.J.'s interests were reviewed and are managed by Massachusetts General Hospital and Partners HealthCare in accordance with their conflict of interest policies.

## Supporting information


**Table S1** Summary of Cas‐OFFinder prediction.
**Table S2** List of oligonucleotides used in this study.
**Table S3** Summary of genotyping results of T0 and T1 lines.
**Table S4** Summary of CIRCLE‐seq‐identified off‐targets.

**Data S1** Off‐target mutagenesis analysis.
