# Supplementary material for: Activities and specificities of CRISPR/Cas9 and Cas12a nucleases for targeted mutagenesis in maize
Source: Plant Biotechnol J. 2018 Jul 22;17(2):362–72. doi: 10.1111/pbi.12982 (PMC6320322; doi:10.1111/pbi.12982)
Supplement: Supplementary file 1 — Table S1 Summary of Cas‐OFFinder prediction. Table S2 List of oligonucleotides used in this study. Table S3 Summary of genotyping results of T0 and T1 lines. Table S4 Summary of CIRCLE‐seq‐identified off‐targets. [file PBI-17-362-s001.pdf]

Table S1. Summary of off-targets predicted by CasOffinder.

gRNA	Target Sequence	Gap	Mismatch	# off-targets predicted
crRNA1	TTTNGTCACAGATCACAAACTTCAAAT	0	4	1
crRNA1	TTTNGTCACAGATCACAAACTTCAAAT	0	5	10
crRNA1	TTTNGTCACAGATCACAAACTTCAAAT	-1	1	3
crRNA1	TTTNGTCACAGATCACAAACTTCAAAT	-1	2	1
crRNA1	TTTNGTCACAGATCACAAACTTCAAAT	-1	3	5
crRNA1	TTTNGTCACAGATCACAAACTTCAAAT	-1	4	74
crRNA1	TTTNGTCACAGATCACAAACTTCAAAT	-1	5	1099
**crRNA1 Total**				**1193**
crRNA2	TTTNAACAAGTGGGCGCAGATCCTGAG	-1	2	1
crRNA2	TTTNAACAAGTGGGCGCAGATCCTGAG	-1	3	3
crRNA2	TTTNAACAAGTGGGCGCAGATCCTGAG	-1	4	14
crRNA2	TTTNAACAAGTGGGCGCAGATCCTGAG	-1	5	155
**crRNA2 Total**				**173**
gRNA1	ACAGATCACAAACTTCAAATGNGG	0	2	1
gRNA1	ACAGATCACAAACTTCAAATGNGG	0	4	19
gRNA1	ACAGATCACAAACTTCAAATGNGG	0	5	249
gRNA1	ACAGATCACAAACTTCAAATGNGG	-1	1	1
gRNA1	ACAGATCACAAACTTCAAATGNGG	-1	2	3
gRNA1	ACAGATCACAAACTTCAAATGNGG	-1	3	69
gRNA1	ACAGATCACAAACTTCAAATGNGG	-1	4	1083
gRNA1	ACAGATCACAAACTTCAAATGNGG	-1	5	17604
**gRNA1 Total**				**19029**
gRNA2	AAGTGGGCGCAGATCCTGAGNGG	0	2	12
gRNA2	AAGTGGGCGCAGATCCTGAGNGG	0	3	357
gRNA2	AAGTGGGCGCAGATCCTGAGNGG	0	4	3699
gRNA2	AAGTGGGCGCAGATCCTGAGNGG	0	5	2255
gRNA2	AAGTGGGCGCAGATCCTGAGNGG	-1	1	13
gRNA2	AAGTGGGCGCAGATCCTGAGNGG	-1	2	378
gRNA2	AAGTGGGCGCAGATCCTGAGNGG	-1	3	4205
gRNA2	AAGTGGGCGCAGATCCTGAGNGG	-1	4	9280
gRNA2	AAGTGGGCGCAGATCCTGAGNGG	-1	5	51054
**gRNA2 Total**				**71253**

Table S2. Oligonucleotides used in this study.

Forward primer	Sequence	Reverse primer	Sequence	Sequencing primer	Sequence
**On-target analysis**					
Zm-gl2-F2	CACAGCCTTGCAATCAATTC	Zm-gl2-R2	GCTGACGTGGAAGGAGTAGC	ZmGl2-exon2-F1	ACACCGTGTCTTCGTCAAAA
ZmGl2-exon2-F1	ACACCGTGTCTTCGTCAAAA	ZmGl2-exon2-R1	GACCAGCCACAGGTCCTC	ZmGl2-exon2-F1	ACACCGTGTCTTCGTCAAAA
**Cas9-gRNA1 off-target analysis**					
**CIRCLE-seq**					
CIRgRNA1-OFF1F	AGTGACTACATGGGGGCAAC	CIRgRNA1-OFF1R	CTGGCGATGTCGATCTCTTT	CIRgRNA1-OFF1seq	CACTATCGCCCCCTTGATTA
CIRgRNA1-OFF2F	GAGCACGGTGACGCTATGTA	CIRgRNA1-OFF2R	TTCGAAAGGATGTCAATCTGG	CIRgRNA1-OFF2seq	CTCTCACTTGCTGGCAAAAA
CIRgRNA1-OFF4F	TGGGGAGACTGAACATAGCA	CIRgRNA1-OFF4R	AGTGGTCCAGTTCCCAACAG	CIRgRNA1-OFF4seq	TGCATATCCGACCAGAAACA
CIRgRNA1-OFF5F	GAATGTCAGACTCGCCTTCC	CIRgRNA1-OFF5R	TCGCTGCTTCTGCTGTCTAC	CIRgRNA1-OFF5seq	CACTTCCCCTAAAGCAAGCA
CIRgRNA1-OFF6F	GAATGCGGATGCTTTTGTTT	CIRgRNA1-OFF6R	GAGCCTTTTGGGTTGGAGTT	CIRgRNA1-OFF6seq	TATGGGTCGTGCCATCTTTT
CIRgRNA1-OFF7F	TGGGAATTGGTTTCCTCTTG	CIRgRNA1-OFF7R	GGCATGGAAACTGTCTTGCT	CIRgRNA1-OFF7seq	TGGGAATTGGTTTCCTCTTG
CIRgRNA1-OFF8F	AAACCCACCGCTTCTGTAAA	CIRgRNA1-OFF8R	CTCCCGCTAGCACTTGAATC	CIRgRNA1-OFF8seq	GAATCGCCATTGTAAGAGCA
CIRgRNA1-OFF9F	AATTGGAGGGAAGGCTAGGA	CIRgRNA1-OFF9R	GAACCAAATGCGGAAACTGT	CIRgRNA1-OFF9seq	TAGGATGGGAATGCTTGAGG
CIRgRNA1-OFF10F	AAGCAACCAACTGGCACTGT	CIRgRNA1-OFF10R	GGCAAGCGGCTATTTTATCA	CIRgRNA1-OFF10seq	TGGCACTGTTCATCTTGTGC
CIRgRNA1-OFF11F	CCTTTCAGATCGTGCCTTTC	CIRgRNA1-OFF11R	TTGGTGTTTCCTCTCCCATC	CIRgRNA1-OFF11seq	GCCCCTAGCAAGAACCTTTT
CIRgRNA1-OFF12F	CCCGAAAGAAGGCTTCACTA	CIRgRNA1-OFF12R	GTTTTTGTACCGGCCTAACG	CIRgRNA1-OFF12seq	ATTCCAACAGCTTGCTCCAC
CIRgRNA1-OFF13F	AGAGTGCCAAGGACAACACC	CIRgRNA1-OFF13R	TTGAGCAACCCTAAGCAAGG	CIRgRNA1-OFF13seq	CGTGCCATTTATGAGTCCAA
CIRgRNA1-OFF14F	ACACAAAGAATGCGGGTGAT	CIRgRNA1-OFF14R	GGTTGGACCGTTGGAGACTA	CIRgRNA1-OFF14seq	TGATTTGTTTCCCGAGGTTC
CIRgRNA1-OFF16F	TCCATTCCATTGATTGCTGA	CIRgRNA1-OFF16R	GGCCCTTCATGATCAGTGTT	CIRgRNA1-OFF16seq	GGAGAACCGTGGTCTGTCTT
CIRgRNA1-OFF17F	AAATGCTGGCCAAATTGTCT	CIRgRNA1-OFF17R	GTCCACCCATGCCTGATACT	CIRgRNA1-OFF17seq	GGCCAAATTGTCTGAAAAGG
**Cas-OFFinder**					
CASgRNA1-OFF1F	GCAGGTTGGGGAGACTGAAC	CASgRNA1-OFF1R	CAACCTACTGAACGGGACCG	CASgRNA1-OFF1seq	GCAGGTTGGGGAGACTGAAC
CASgRNA1-OFF2F	GGACAGTTTTTCCCACCCCT	CASgRNA1-OFF2R	CCTTCGTCGAGCAAGCTACA	CASgRNA1-OFF2seq	GGACAGTTTTTCCCACCCCT
**Cas9-gRNA2 off-target analysis**					
**CIRCLE-seq**					
CIRgRNA2-OFF3F	TTTTACCCACGCAGTCCAGT	CIRgRNA2-OFF3R	AGCCCTGACAAGGACGTCTA	CIRgRNA2-OFF3seq	TTCCTTTGTCCTCCTCAGCA
CIRgRNA2-OFF7F	GTGCCAAAAGCAGCTTATCC	CIRgRNA2-OFF7R	TGCATATAGCGCCAAAGATG	CIRgRNA2-OFF7seq	GCCTTTGCTTGTGCGATAAT
CIRgRNA2-OFF8F	TTGGTTGAGAGGGTTTCAGC	CIRgRNA2-OFF8R	CGGTCCATACCATGACACAG	CIRgRNA2-OFF8seq	GGTCCGTTTGGATTTGAGAA

Table S2. Oligonucleotides used in this study.

Forward primer	Sequence	Reverse primer	Sequence	Sequencing primer	Sequence
CIRgRNA2-OFF12F	GACCACTTTCCCACTCACAGA	CIRgRNA2-OFF12R	ACCCAGAAAGGGAAAAGGAG	CIRgRNA2-OFF12seq	GTGTCCTCGCGCAACACTA
CIRgRNA2-OFF13F	CATCGCGCCTTCTCATCTAT	CIRgRNA2-OFF13R	CTGGTGCTCTTTTCCCTCTG	CIRgRNA2-OFF13seq	TAATGAGTGGGACCCTCTGG
CIRgRNA2-OFF14F	CTTCTTCTTGGCCACCAGAC	CIRgRNA2-OFF14R	GTCGGCGATGTAGGTCGATA	CIRgRNA2-OFF14seq	GCACCTGCATCACCTAGCC
CIRgRNA2-OFF15F	CCTTCCCTCTTCTGGTCTCC	CIRgRNA2-OFF15R	AGGATCCGCATCTCCTTCTT	CIRgRNA2-OFF15seq	CGCTCTCAGATCCCTCTCC
CIRgRNA2-OFF16F	TTTATCAGCTTCGGGTCGAT	CIRgRNA2-OFF16R	GAAAGCTCCCTCGGAAACTC	CIRgRNA2-OFF16seq	GCTCAAGCCACGATGTATCA
CIRgRNA2-OFF17F	GCCTGCTTTAGCTCGCTATG	CIRgRNA2-OFF17R	TCAAGTTCTCCCGTGGAGTT	CIRgRNA2-OFF17seq	CATACTTGCCGCGATCCTAT
CIRgRNA2-OFF19F	CCAAAAGGGGTGGAGTTTTA	CIRgRNA2-OFF19R	CCGTTTCACGTAGGAGCATT	CIRgRNA2-OFF19seq	TGGCCAGGCCTTATTTGTTA
CIRgRNA2-OFF21F	TCTGATGATGGCATTCCTGA	CIRgRNA2-OFF21R	GCTTTAGCACCTGGAAAGCA	CIRgRNA2-OFF21seq	GAGAAGCATTCGTTCATTTGC
CIRgRNA2-OFF22F	GACGGTGTACGGCAACAA	CIRgRNA2-OFF22R	TTGTGCTTGTTGATGTGGTG	CIRgRNA2-OFF22seq	CGTCGACTTTCTCCGTGAAG
CIRgRNA2-OFF23F	CTGGCATTGCACAGTCGTAT	CIRgRNA2-OFF23R	GTCGCAAATCCTTGATCCAC	CIRgRNA2-OFF23seq	AGTCGTATCGTGGTCCTTCG
CIRgRNA2-OFF24F	CAGGGTTATTGGTGGGAGTG	CIRgRNA2-OFF24R	ACTACTCGCGGATGGAGTTG	CIRgRNA2-OFF24seq	AAGAGGGACCATCTGCTGTG
CIRgRNA2-OFF25F	GGGAGTGTACGCAACTTCCTA	CIRgRNA2-OFF25R	TGGCTGCTTGCTACTGTCAC	CIRgRNA2-OFF25seq	TGGGGTATTTGGTTTCATCC
CIRgRNA2-OFF28F	GGTTTGCAGAGGTGGTGACT	CIRgRNA2-OFF28R	CCAGGATGGCTATGAAGCTC	CIRgRNA2-OFF28seq	GTGAAGTCCCTGGACGAATG
CIRgRNA2-OFF29F	TTTGCAGCCTCGTCTGTCTA	CIRgRNA2-OFF29R	TCCCAAGTCCACACTCTCCT	CIRgRNA2-OFF29seq	CAACCATGCCCACATGAGTA
CIRgRNA2-OFF32F	AATTTTGGTTTCTCGGATCG	CIRgRNA2-OFF32R	ATACCCCTCCGTTTTGTCCT	CIRgRNA2-OFF32seq	TCGGATCGCGTGATACTACA
CIRgRNA2-OFF36F	GTCTTCACGACGGTCACCA	CIRgRNA2-OFF36R	CAGGTCACCAGCTTCAAGTGT	CIRgRNA2-OFF36seq	GTGACGTGGAAGGAGAAGGA
CIRgRNA2-OFF38F	GCTAATAATTTCCCGCAACG	CIRgRNA2-OFF38R	TGGATAGACTGGACTGGATGG	CIRgRNA2-OFF38seq	GAGACCGACGGACCTGTAAA
CIRgRNA2-OFF39F	CTCCAGAGGGAGGACAAATG	CIRgRNA2-OFF39R	CCGTCGTTAGGCAAAAACAC	CIRgRNA2-OFF39seq	AGGGAGGACAAATGCGAAG
CIRgRNA2-OFF41F	AAACCTGACCCTGATCCAAA	CIRgRNA2-OFF41R	AGTAGAAATCCGGCGAACCT	CIRgRNA2-OFF41seq	TCCCTTGCCTGCAATGTC
CIRgRNA2-OFF46F	AGCTCAGGTACACGCTGCTC	CIRgRNA2-OFF46R	CGGGTCTTTCTGGTCTTCAA	CIRgRNA2-OFF46seq	GAGCTCAAACGCTTCGCTAT
CIRgRNA2-OFF49F	TCACGAAAGGAACGAAAGGT	CIRgRNA2-OFF49R	TTTTCCATGGAGCCAGATTC	CIRgRNA2-OFF49seq	CGATGAGCACCAGTCATTGT
CIRgRNA2-OFF54F	GTGTCCCTGTTTCCATGACC	CIRgRNA2-OFF54R	ACATAGCCCTCCCCTAGCAT	CIRgRNA2-OFF54seq	GGGTGTTAGGGAGGGAGAAG
**Cas-OFFinder**					
CASgRNA2-OFF1F	TAGGCAGACAATGAGCCAAA	CASgRNA2-OFF1R	TAGATCGAGAAGGCGTCCTG	CASgRNA2-OFF1seq	CCAAAACTAACCTGAGCGATCT
CASgRNA2-OFF4F	CCGCAGATAAGACACTGACG	CASgRNA2-OFF4R	GTGACGTGGAAGGAGAAGGA	CASgRNA2-OFF4seq	CAAGACGACGTGTTCTGCTAA
**T-DNA screening**					
zCas9-F	CCGATTCTGGAGAAGATGGA	zCas9-R	TCGAAGAGATGGGCGTAAGT		
LbCas12a-F	AATGGAACGCGGAGTATGAC	LbCas12a-R	ACATGTCGCCCTTCTTGAAC		

Table S2. Oligonucleotides used in this study.

Forward primer	Sequence	Reverse primer	Sequence	Sequencing primer	Sequence
**Vector construction**					
LNK-165-F-NEW	CATGGGGTACCCAATTGTTCCGGAACTCTAGATAAGCTTA				
LNK-165-R-NEW	CCGGTAAGCTTATCTAGAGTTCCGGAACAATTGGGTACCC				
zCas9-F-NcoI	TGGACCATGGATTACAAGGACCACGACGGGGATTA				
zCas9-R-SacI	GTCGAAACCGATGATACGAACGAA				
ZmGL2-crRNA1-F	TAGATGTCACAGATCACAAACTTCAAAT				
ZmGL2-crRNA1-R	GGCCATTTGAAGTTTGTGATCTGTGACA				
ZmGL2-crRNA2-F	TAGATAACAAGTGGGCGCAGATCCTGAG				
ZmGL2-crRNA2-R	GGCCCTCAGGATCTGCGCCCACTTGTTA				
ZmGL2-gR3-F	TGGCACAGATCACAAACTTCAAATG				
ZmGL2-gR3-R	AAACCATTTGAAGTTTGTGATCTGT				
ZmGL2-gR4-F	TGGCAAGTGGGCGCAGATCCTGAG				
ZmGL2-gR4-R	AAACCTCAGGATCTGCGCCCACTT				
**CIRCLE-seq**					
oSQT1288	P-CGGTGGACCGATGATCUATCGGTCCACCG*T		*indicates a phosphorothioate linkage		

Table S3. Summary of genotyping results of T0 and T1 lines.

Construct	Event	Plant	T0 genotype		Method	#clone	Genotype of T1 progenies (pollen donor was wild-type)	Method	Off-target mutations
A842B	1	1	WT	(0, 0)	Cloning	7			
A842B	1	2	WT	(0, 0)	Cloning	7	(0, 0) x 7	TIDE	
A842B	1	3	Heterozygous	(0, -9)	Cloning	2, 6			
A842B	1	4	Mosaic	(0, -7, -9)	Cloning	5, 1, 2	(0,-9), (+1, -8)	TIDE	
A842B	1	5	Heterozygous	(0, -73)	Cloning	7, 1		TIDE	
A842B	2	1	Heterozygous	(0, -11)	TIDE			TIDE	
A842B	2	2	Heterozygous	(0, -12+1)	TIDE		(+1, -10) x 3, (0, -11) x 4	TIDE	
A842B	2	3	Heterozygous	(0, -9)	TIDE				
A842B	2	4	WT	(0, 0)	TIDE				
A842B	2	5	Heterozygous	0/-11	TIDE				
A842B	3	1	WT	(0, 0)	TIDE				
A842B	3	2	WT	(0, 0)	TIDE				
A842B	3	3	WT	(0, 0)	TIDE				
A842B	3	4	WT	(0, 0)	TIDE				
A842B	3	5	WT	(0, 0)	TIDE				
A842B	5	1	Heterozygous	(0, +1)	TIDE		(+1, -6), (+2, -7), (+1, -8), (0, -7) x 2, (0, -9) x 2	TIDE	
A842B	5	3	Mosaic	(+1, -8, -9)	TIDE				
A842B	5	4	Heterozygous	(0, +1)	TIDE				
A842B	5	5	Biallelic	(-6, -9)	TIDE				
A842B	5	7	Heterozygous	(0, -9)	TIDE				
A843B	1	1	WT	(0, 0)	Cloning	7			
A843B	1	2	WT	(0, 0)	Cloning	8			
A843B	1	3	WT	(0, 0)	Cloning	8			
A843B	1	4	WT	(0, 0)	Cloning	5			
A843B	2	1	WT	(0, 0)	Cloning	8			
A843B	2	2	WT	(0, 0)	Cloning	8	(0, 0) x 7	TIDE	
A843B	2	3	WT	(0, 0)	Cloning	8			
A843B	2	4	WT	(0, 0)	Cloning	8			
A843B	2	5	WT	(0, 0)	Cloning	6			
A843B	3	1	WT	(0, 0)	Cloning	5			
A843B	3	2	WT	(0, 0)	Cloning	5	(0, 0) x 7	TIDE	
A843B	3	3	WT	(0, 0)	Cloning	7			
A843B	3	4	WT	(0, 0)	Cloning	3			
A843B	3	5	WT	(0, 0)	Cloning	7			
A843B	3	6	WT	(0, 0)	TIDE				
A843B	4	1	WT	(0, 0)	TIDE				
A843B	4	2	WT	(0, 0)	TIDE				
A843B	4	3	WT	(0, 0)	TIDE				
A843B	4	4	WT	(0, 0)	TIDE				
A843B	4	5	WT	(0, 0)	TIDE				
A843B	4	6	WT	(0, 0)	TIDE				
A843B	6	1	WT	(0, 0)	TIDE				
A843B	6	2	WT	(0, 0)	TIDE				
A844B	1	1	Heterozygous	(0, -7)	TIDE				
A844B	1	2	Homozygous	(-7, -7)	TIDE				
A844B	1	3	Homozygous	(-7, -7)	TIDE				
A844B	1	4	Homozygous	(-7, -7)	TIDE		(+2, -7), (-7, -6), (-1, -7), (0, -7)	TIDE	NO
A844B	1	5	Homozygous	(-7, -7)	TIDE				
A844B	2	1	Homozygous	(+1, +1)	TIDE				
A844B	2	2	Homozygous	(+1, +1)	TIDE				
A844B	2	3	Homozygous	(+1, +1)	TIDE				
A844B	2	4	Homozygous	(+1, +1)	TIDE				
A844B	2	5	Homozygous	(+1, +1)	TIDE		(0,+1) x3	TIDE	NO
A844B	3	2	Homozygous	(-2, -2)	TIDE		(-2,-1), (-2,-5), (-2,-6), (-2,-2), (-2, +1)	TIDE	NO
A844B	3	4	Homozygous	(-2, -2)	Cloning	8	(-2,+1) x 3, (-2,-2)	TIDE	NO
A844B	3	5	Homozygous	(+1, +1)	Cloning	6			

**Construct**	**Event**	**Plant**	**T0 genotype**		**Method**	**#clone**	**Genotype of T1 progenies (pollen donor was wild-type)**	**Method**	**Off-target mutations**
A844B	5	1	Heterozygous	(0, +1)	TIDE				
A844B	5	2	Biallelic	(+1, -6)	TIDE		(+1, +1) x 2, (+1, -2), (0, -6)	TIDE	
A844B	6	1	Heterozygous	(0, +1)	TIDE		(-1,-7), (-7, -7), (0, 0)	TIDE	NO
A844B	6	2	Heterozygous	(0, +1)	TIDE				
A844B	6	3	Heterozygous	(0, +1)	TIDE				
A844B	6	4	Heterozygous	(0, +1)	TIDE				
A844B	6	5	Heterozygous	(0, +1)	TIDE				
A844B	7	1	Heterozygous	(0, +1)	TIDE				
A844B	7	2	Heterozygous	(0, +1)	TIDE		(+1,-1), (+1,-3), (+1, +1)x2, (0, +1)	TIDE	NO
A844B	8	1	Biallelic	(+1, -2)	TIDE				
A844B	8	2	Biallelic	(+1, -2)	TIDE		(-2, -5), (-1,-4), (0, +1), (0, -2)x3	TIDE	NO
A844B	8	3	Heterozygous	(0, +1)	TIDE				
A844B	8	4	Biallelic	(+1, -2)	TIDE				
A844B	8	5	Biallelic	(+1, -2)	TIDE				
A845B	1	1	WT	(0, 0)	Cloning	8			
A845B	1	2	WT	(0, 0)	Cloning	6			
A845B	2	1	Homozygous	(-6, -6)	Cloning	5			
A845B	2	2	Biallelic	(-6, -4+1)	Cloning	6, 2			
A845B	2	3	Biallelic	(-6, -4+1)	Cloning	4, 3			
A845B	2	4	Biallelic	(-6, -4+1)	Cloning	4, 4	(-2, -5), (0, -6), (0,-3) x 2	TIDE	NO
A845B	2	5	Biallelic	(-6, -4+1)	Cloning	5, 3			
A845B	3	1	Biallelic	(-2, -5)	Cloning	4, 4			
A845B	3	2	Biallelic	(-2, -5)	Cloning	1, 5			
A845B	3	3	Biallelic	(-2, -5)	Cloning	5, 3	(-2, -2), (-2, -13), (-2, -15), (-2, -24), (-5, -5), (-5, -24), (-5, -27)	TIDE	NO
A845B	3	4	Biallelic	(-2, -5)	Cloning	1, 7	(-2, -2), (-5, -1)	TIDE	NO
A845B	3	5	Biallelic	(-2, -5)	Cloning	4, 3			
A845B	3	6	Biallelic	(-2, -5)	Cloning	5, 3			
A845B	4	1	Biallelic	(-2, -4)	Cloning	10, 9	(-4, -1) x 2, (-4, -5), (0, -4)	TIDE	NO
A845B	4	3	Biallelic	(-2, -4)	Cloning	4, 2	(-2, -2), (-4, -1), (-4, +4), (0, -4)x2	TIDE	NO
A845B	4	4	Biallelic	(-2, -4)	Cloning	2, 4			
A845B	5	1	Biallelic	(-2, -3)	Cloning	4, 2			
A845B	5	2	Biallelic	(-2, -3)	Cloning	5, 3			
A845B	5	3	Biallelic	(-2, -3)	Cloning	1, 5	(-1, -4), (-3, -9), (-3, -16), (-3, -6), (0, -3)	TIDE	NO
A845B	6	1	Homozygous	(-6, -6)	Cloning	8			
A845B	6	2	Homozygous	(-6, -6)	Cloning	8			
A845B	6	3	Homozygous	(-6, -6)	Cloning	8			
A845B	6	4	Homozygous	(-6, -6)	Cloning	6	(-1, -6)x2, (0, -6)	TIDE	NO
A845B	6	5	Homozygous	(-6, -6)	Cloning	7			
A845B	7	1	Biallelic	(-1, -6)	TIDE				
A845B	7	3	Biallelic	(-1, -6)	TIDE		(-1, -1), (-2, +1), (-1, -5), (-1, -6)	TIDE	NO
A845B	8	1	Heterozygous	(0, -6)	Cloning	7, 1			
A845B	8	2	Heterozygous	(0, -4)	Cloning	7, 1			
A845B	8	3	WT	(0, 0)	Cloning	8	(-7, -20), (0, -4), (0, 0)x2	TIDE	NO
A845B	8	5	Heterozygous	(0, -3)	Cloning	6, 1			
A845B	10	2	Heterozygous	(0, -6)	TIDE		(0, -6), (0, 0)	TIDE	NO

Table S4. List of off-targets identified by CIRCLE-seq and Cas-OFFinder.

	Off-target	Chr.	OFF-target position	Strand	# mis-matches	Gap	CIRCLE-seq read	Off-target sequence	F primer	R primer	Sequencing primer	Sequencing ID
**Cas12a-crRNA1: CasOFFinder**												
1	CAScrRNA1-OFF1	chr2	16677669	-	3	-1		TTTCTTCCAGATCATAAAATTCAAAT	CACTATCGCCCCCTTGATTA	CTGGCGATGTCGATCTCTTT	CACTATCGCCCCCTTGATTA	gRNA1-OFF1
**Cas9-gRNA1: CIRCLE-seq**												
1	CIRgRNA1-OFF1	chr8	74971851	-	2	-1	754	A-AGATCACAAGCCTCAAATGAGG	CACTATCGCCCCCTTGATTA	CTGGCGATGTCGATCTCTTT	CACTATCGCCCCCTTGATTA	gRNA1-OFF1
2	Target	chr2	8112521	-	0		672	ACAGATCACAAACTTCAAATGCGG	CACAGCCTTGCAATCAATTC	GCTGACGTGGAAGGAGTAGC	ACACCGTGTCTTCGTCAAAA	
3	CIRgRNA1-OFF2	chr10	41964030	-	6		236	GTTTAACACAAACTTCAAATTTGG	TTGATGGTCATCAGTTTGGTG	TTCGAAAGGATGTCAATCTGG	TTGATGGTCATCAGTTTGGTG	gRNA1-OFF2
4	CIRgRNA1-OFF3	chr2	143072090	+	6		80	TTTGAACACGAACTTCACATGGGG	NA	NA	NA	
5	CIRgRNA1-OFF4	chr3	107726662	+	3	-1	60	ACAAATCATAAACTTGAA-TGTGG	TTGAAAACCATCCTGGTGCT	TCTTGCAGGAGCATAGCAAA	TTGAAAACCATCCTGGTGCT	gRNA1-OFF3
6	CIRgRNA1-OFF5	chr3	108808715	+	2		56	ACAGATCACAAAGGTCAAATGTGG	GAATGTCAGACTCGCCTTCC	TCGCTGCTTCTGCTGTCTAC	CACTTCCCCTAAAGCAAGCA	gRNA1-OFF4
7	CIRgRNA1-OFF6	chr3	1731070	-	3	-1	44	ATAGACCACAA-CTTCAAATGGGC	GAATGCGGATGCTTTTGTTT	GAGCCTTTTGGGTTGGAGTT	TATGGGTCGTGCCATCTTTT	gRNA1-OFF5
8	CIRgRNA1-OFF7	chr1	34341100	-	4		36	TCAGATTACAAATTTCAACTGAGG	TGGGAATTGGTTTCCTCTTG	GGCATGGAAACTGTCTTGCT	TGGGAATTGGTTTCCTCTTG	gRNA1-OFF6
9	CIRgRNA1-OFF8	chr4	103904220	-	3	-1	30	AAAAATCACAAACTTCATA-GGGG	TGGTAAACAAACCGGGTCTAA	CTCCCGCTAGCACTTGAATC	CTCCCGCTAGCACTTGAATC	gRNA1-OFF7
10	CIRgRNA1-OFF9	chr10	52293332	+	7		28	AACGATCAAAAACTTCAATATGGT	CCCACAGCCCCACTAATACA	CATGCAATTGAGCTGGAGAA	CCCACAGCCCCACTAATACA	gRNA1-OFF8
11	CIRgRNA1-OFF10	chr2	119407415	+	3	1	24	CGAAATCACAAAGTTCCAATGGGG	TTTGAAACATTGCAGCATGA	TCAAATGCAGCAGGTCAGTC	TTTGAAACATTGCAGCATGA	gRNA1-OFF9
12	CIRgRNA1-OFF11	chr8	72728483		7		24	TTTAAACACAAACTTCACATTGGG	GCCCCTAGCAAGAACCTTTT	CACCATTCCCGCACTCTAAT	GCCCCTAGCAAGAACCTTTT	gRNA1-OFF10
13	CIRgRNA1-OFF12	chr5	134878299	-	4	-1	50	ATCAATCACAAA-TTCAAATATGG	CCCGAAAGAAGGCTTCACTA	GTTTTTGTACCGGCCTAACG	ATTCCAACAGCTTGCTCCAC	gRNA1-OFF13
14	CIRgRNA1-OFF13	scaffold5.1434	9927	+	7		154	TTTAATCACAAACTTCAATATGGG	AGAGTGCCAAGGACAACACC	TTGAGCAACCCTAAGCAAGG	CGTGCCATTTATGAGTCCAA	gRNA1-OFF14
15	CIRgRNA1-OFF14	scaffold3.573	33430	-	3	-1	132	AATGATCACAATCT-CAAATGTGG	ACACAAAGAATGCGGGTGAT	GGTTGGACCGTTGGAGACTA	TGATTTGTTTCCCGAGGTTC	gRNA1-OFF15
16	CIRgRNA1-OFF15	scaffold7.852	31292	-	5		124	TTTGAACACAAACTTCACATGGGG	NA	NA	NA	
17	CIRgRNA1-OFF16	scaffold9.645	123031	-	5		70	TGACATCACAAATTTCAAATTTGG	TCCATTCCATTGATTGCTGA	GGCCCTTCATGATCAGTGTT	GGAGAACCGTGGTCTGTCTT	gRNA1-OFF16
18	CIRgRNA1-OFF17	scaffold4.2924	93708	-	5		28	TCCTATCACACACTTCAAATGGAG	AAATGCTGGCCAAATTGTCT	GTCCACCCATGCCTGATACT	GGCCAAATTGTCTGAAAAGG	gRNA1-OFF17
19	CIRgRNA1-OFF18	scaffold1.1776	51589	+	6		26	AATCTTCACAAGCTTCCAATGGGG	NA	NA	NA	
**Cas9-gRNA1: Cas-OFFinder**												
1	CASgRNA1-OFF1	chr3	107726661	+	3	-1		ACAAATCATAAACTTGAATGTGG	GCAGGTTGGGGAGACTGAAC	CAACCTACTGAACGGGACCG	TGCATATCCGACCAGAAACA	gRNA1-OFF11
2	CASgRNA1-OFF2	chr3	123022309	+	3	-1		ACAGTCACACACTTCCAAAGAGG	GGACAGTTTTTCCCACCCCT	CCTTCGTCGAGCAAGCTACA	GCACCACCAAAGAGACCAAT	gRNA1-OFF12
**Cas9-gRNA2: CIRCLE-seq**												
1	CIRgRNA2-OFF1	chr9	13588581	+	6		342	CGTTGGGCCCAGATCCAGAGGCG	NA	NA	NA	
2	CIRgRNA2-OFF2	chr7	89451810	+	6		294	CGTTGGGCCCAGATCCAGAGGCG	NA	NA	NA	
3	CIRgRNA2-OFF3	chr1	141204025	-	4	-1	278	CGGTGGCAGCAGATCCTG-GTGG	TTTTACCCACGCAGTCCAGT	AGCCCTGACAAGGACGTCTA	TTCCTTTGTCCTCCTCAGCA	gRNA2-OFF1
4	CIRgRNA2-OFF4	chr5	97442353	+	4	-1	252	AA-TGGACACAGATCATGAGGAG	NA	NA	NA	
5	CIRgRNA2-OFF5	chr10	20775888	+	4	-1	190	AA-TGGACACAGATCATGAGGAG	NA	NA	NA	
6	CIRgRNA2-OFF6	chr4	3175346	-	4		176	AAGCGGGCGTAGCTCCAGAGGGG	NA	NA	NA	
7	CIRgRNA2-OFF7	chr4	111140164	+	5		158	TTGTAGTCGCAGATCCTGAGCAG	GTGCCAAAAGCAGCTTATCC	TGCATATAGCGCCAAAGATG	GCCTTTGCTTGTGCGATAAT	gRNA2-OFF2
8	CIRgRNA2-OFF8	chr9	77561370	+	5		154	CAGTAGGTGCAGATCCTGAGGAT	TTGGTTGAGAGGGTTTCAGC	CGGTCCATACCATGACACAG	GGTCCGTTTGGATTTGAGAA	gRNA2-OFF3

Table S4. List of off-targets identified by CIRCLE-seq and Cas-OFFinder.

	Off-target	Chr.	OFF-target position	Strand	# mis-matches	Gap	CIRCLE-seq read	Off-target sequence	F primer	R primer	Sequencing primer	Sequencing ID
9	CIRgRNA2-OFF9	chr8	114426202	+	7		132	AAAGGGGCATAGATCCTGGTGGT	NA	NA	NA	
10	CIRgRNA2-OFF10	chr3	80437794	+	5		122	AAGGGGTCGTAGGTCATGAGTGG	NA	NA	NA	
11	CIRgRNA2-OFF11	chr1	71431596	-	4		120	AAGTGGGTACAGTTCATGAGGGG	NA	NA	NA	
12	CIRgRNA2-OFF12	chr9	43647126	-	7		118	GCATGGGCGCAGATCATGGAGGT	NA	NA	NA	
13	CIRgRNA2-OFF13	chr9	29274484	+	6		114	GCGTAGGCCCAGATCATGAGGAG	CATCGCGCCTTCTCATCTAT	CTGGTGCTCTTTTCCCTCTG	TAATGAGTGGGACCCTCTGG	gRNA2-OFF4
14	CIRgRNA2-OFF14	chr5	65412390	-	7		96	TCCTGGGTGCAGACCCTGCGGAG	NA	NA	NA	
15	CIRgRNA2-OFF15	chr7	13681611		3	-1	92	TTGTGGGGGCAGATC-TGAGCGG	CCTTCCCTCTTCTGGTCTCC	AGGATCCGCATCTCCTTCTT	CGCTCTCAGATCCCTCTCC	gRNA2-OFF5
16	CIRgRNA2-OFF16	chr4	89100310	-	4		90	AAGCGGGTGCAGAACATGAGAGG	TTTATCAGCTTCGGGTCGAT	GAAAGCTCCCTCGGAAACTC	GCTCAAGCCACGATGTATCA	gRNA2-OFF6
17	Target	chr2	8112430	-	0		86	AAGTGGGCGCAGATCCTGAGCGG	CACAGCCTTGCAATCAATTC	GCTGACGTGGAAGGAGTAGC	ACACCGTGTCTTCGTCAAAA	
18	CIRgRNA2-OFF17	chr9	53910322	-	4		80	AGGGGGGTGCAGATCGTGAGAGG	GCCTGCTTTAGCTCGCTATG	TCAAGTTCTCCCGTGGAGTT	CATACTTGCCGCGATCCTAT	gRNA2-OFF7
19	CIRgRNA2-OFF18	chr4	131654621	-	4		74	TAGTGGGCACGGATCCCGAGTGG	NA	NA	NA	
20	CIRgRNA2-OFF19	chr8	124038726	-	7		72	TTGTAGGGACAGATCCTGACGGC	CCAAAAGGGGTGGAGTTTTA	CCGTTTCACGTAGGAGCATT	TGGCCAGGCCTTATTTGTTA	gRNA2-OFF8
21	CIRgRNA2-OFF20	chr10	10741673	-	4	-1	68	ATGTGGGCACAGATCCAT-GGGG	NA	NA	NA	
22	CIRgRNA2-OFF21	chr5	99843540	+	4	-1	64	AGGTAGGCTCAGATCCT-ATTGG	TCTGATGATGGCATTCCTGA	GCTTTAGCACCTGGAAAGCA	GAGAAGCATTCGTTCATTTGC	gRNA2-OFF9
23	CIRgRNA2-OFF22	chr2	143394415	+	4	-1	60	GTGGAGGCGCAGATCCTG-GAGG	NA	NA	NA	
24	CIRgRNA2-OFF23	chr8	3233767	-	5		58	GTGTGGGTGCATATCCTGACGGG	NA	NA	NA	
25	CIRgRNA2-OFF24	chr4	102327148	+	6		52	AGTTGGACGCGGAGCCTGAGGAG	CAGGGTTATTGGTGGGAGTG	ACTACTCGCGGATGGAGTTG	AAGAGGGACCATCTGCTGTG	gRNA2-OFF10
26	CIRgRNA2-OFF25	chr4	120978466	+	3	-1	48	AA-TGGGTGCAAATCCTGGGAGG	GGGAGTGTACGCAACTTCCTA	TGGCTGCTTGCTACTGTCAC	TGGGGTATTTGGTTTCATCC	gRNA2-OFF11
27	CIRgRNA2-OFF26	chr8	35653730	-	7		48	CTGTGGGCACAGATCCATGGGGA	NA	NA	NA	
28	CIRgRNA2-OFF27	chr5	71309072	-	6		46	TTGTGGACACAGATCCCTAGGGG	NA	NA	NA	
29	CIRgRNA2-OFF28	chr10	2615234	-	5		44	AGCGGGGCACAGGTCCTGAGGGG	GGTTTGCAGAGGTGGTGACT	CCAGGATGGCTATGAAGCTC	GTGAAGTCCCTGGACGAATG	gRNA2-OFF12
30	CIRgRNA2-OFF29	chr3	37735259		4	-1	44	CAGTGGGTGCCAATCCT-AGTGG	TTTGCAGCCTCGTCTGTCTA	TCCCAAGTCCACACTCTCCT	CAACCATGCCCACATGAGTA	gRNA2-OFF13
31	CIRgRNA2-OFF30	chr8	45766735		3	-1	40	GAGAGGGGGCAGATCC-GAGCGG	NA	NA	NA	
32	CIRgRNA2-OFF31	chr3	22392038	+	4		38	AAGTGGGCAGAGTTCATGAGGGG	NA	NA	NA	
33	CIRgRNA2-OFF32	chr3	77520472	-	7		34	TCGTGAGTCCAGATCCTGACGGC	AATTTTGGTTTCTCGGATCG	ATACCCCTCCGTTTTGTCCT	TCGGATCGCGTGATACTACA	gRNA2-OFF14
34	CIRgRNA2-OFF33	chr5	104806341	-	3		34	TAGTGGGCGCGGAGCCTGAGTGG	NA	NA	NA	
35	CIRgRNA2-OFF34	chr5	34959898	-	4		34	AAGTAGGCGGAGTTCATGAGGGG	NA	NA	NA	
36	CIRgRNA2-OFF35	chr8	28495318	-	5		34	AGGTGGGCATAGATGCTGAGGCG	NA	NA	NA	
37	CIRgRNA2-OFF36	chr10	31243099	-	3		32	AAGTGGGCGCAGGTCCTCGGCGG	NA	NA	NA	
38	CIRgRNA2-OFF37	chr4	146758923	-	3	-1	32	AGGAGGGCGCAGATC-TGATGGG	NA	NA	NA	
39	CIRgRNA2-OFF38	chr5	122305522	+	4	-1	32	ATGTGAGGGCAGATCCCG-GCGG	GCTAATAATTTCCCGCAACG	TGGATAGACTGGACTGGATGG	GAGACCGACGGACCTGTAAA	gRNA2-OFF15
40	CIRgRNA2-OFF39	chr9	44365953	+	4		32	TAGCGGGCGGAGATCCTGCGGGG	NA	NA	NA	
41	CIRgRNA2-OFF40	chr3	33755704	+	3		30	TAGTGGGCGCGGAGCCTGAGTGG	NA	NA	NA	
42	CIRgRNA2-OFF41	chr4	31939351		4	-1	30	CAGTGGGACAAGATC-TGAGCGG	NA	NA	NA	
43	CIRgRNA2-OFF42	chr1	72856206	-	3		28	TAGTGGGCGCGGAGCCTGAGTGG	NA	NA	NA	
44	CIRgRNA2-OFF43	chr5	126727666	-	2		28	TAGTGGGCGCAGAGCCTGAGTGG	NA	NA	NA	
45	CIRgRNA2-OFF44	chr7	46673844	+	4	-1	28	TA-TGGGCACGGATCCTGGGTGG	NA	NA	NA	

Table S4. List of off-targets identified by CIRCLE-seq and Cas-OFFinder.

	Off-target	Chr.	OFF-target position	Strand	# mis-matches	Gap	CIRCLE-seq read	Off-target sequence	F primer	R primer	Sequencing primer	Sequencing ID
46	CIRgRNA2-OFF45	chr1	42186271	+	3		26	TAGTGGGCGCGGATCCCGAGTGG	NA	NA	NA	
47	CIRgRNA2-OFF46	chr6	3885198	-	5		24	CGGTCGGCGCAGATCCAGAAGGG	AGCTCAGGTACACGCTGCTC	CGGGTCTTTCTGGTCTTCAA	GAGCTCAAACGCTTCGCTAT	gRNA2-OFF16
48	CIRgRNA2-OFF47	chr6	19911665	-	4		22	AAGTGGGCAGAGTTCATGAGGGG	NA	NA	NA	
49	CIRgRNA2-OFF48	chr8	78599198	+	3		22	TAGTGGGCGCGGAGCCTGAGTGG	NA	NA	NA	
50	CIRgRNA2-OFF49	scaffold5.1481	2130	+	5		290	ATATGGGAGAAGATCCTGAGGGT	TCACGAAAGGAACGAAAGGT	TTTTCCATGGAGCCAGATTC	CGATGAGCACCAGTCATTGT	gRNA2-OFF19
51	CIRgRNA2-OFF50	scaffold7.1524	51025	+	6		168	TGGTGGGCCCAGATCATGATGGA	NA	NA	NA	
52	CIRgRNA2-OFF51	scaffold5.1556	182655	+	7		134	CCGAGGGCGCAAATCCTGGTGGT	NA	NA	NA	
53	CIRgRNA2-OFF52	scaffold5.1846	47657	+	6		100	GAGGGGGCGCAGCTCCTAGGGGC	NA	NA	NA	
54	CIRgRNA2-OFF53	scaffold1.2368	3376	+	7		58	CTCAAGGCGCAGATCGTGAGGCG	NA	NA	NA	
55	CIRgRNA2-OFF54	scaffold7.2180	31663	-	4	-1	58	TTGAGGGCGCAGATC-TGTGAGG	GTGTCCCTGTTTCCATGACC	ACATAGCCCTCCCCTAGCAT	GGGTGTTAGGGAGGGAGAAG	gRNA2-OFF20
56	CIRgRNA2-OFF55	scaffold7.857	39835	-	7		56	CTGTGGACACAGATCCTTGGGGA	NA	NA	NA	
57	CIRgRNA2-OFF56	scaffold7.361	17864	+	4		54	TAGTGGGCGCGGATCCCAAGTGG	NA	NA	NA	
58	CIRgRNA2-OFF57	scaffold5.414	18095	+	6		52	GCGGAGGCGCAGATCCCGAGCAG	NA	NA	NA	
59	CIRgRNA2-OFF58	scaffold7.575	108704	+	4		42	AAGTGGACGGAGCTCATGAGGGG	NA	NA	NA	
60	CIRgRNA2-OFF59	scaffold1.3684	8306	+	3		36	AAGTGGGCGGAGTTCATGAGGGG	NA	NA	NA	
61	CIRgRNA2-OFF60	scaffold9.2352	20721	-	7		36	TTGCAGGCGAAGACCCTGAGCAG	NA	NA	NA	
62	CIRgRNA2-OFF61	scaffold10.306	139567	-	6		34	GAATGGACACAGATCGTGAGGAG	NA	NA	NA	
63	CIRgRNA2-OFF62	scaffold4.1476	188749	+	7		32	ATGTGGACACAGATCCATGGGGA	NA	NA	NA	
64	CIRgRNA2-OFF63	scaffold3.2145	3787	+	6		30	GAGGGAGCGCAGATCACGGGGGG	NA	NA	NA	
65	CIRgRNA2-OFF64	scaffold3.903	101030	+	3		30	TAGTGGGCGCAGAGCCCGAGTGG	NA	NA	NA	
66	CIRgRNA2-OFF65	scaffold4.843	7341	+	4		28	AAGTGGGCAGAGTTCATGAGGGG	NA	NA	NA	
67	CIRgRNA2-OFF66	scaffold5.1936	55512	+	4		28	AAGTGGGCAGAGTTCATGAGGGG	NA	NA	NA	
68	CIRgRNA2-OFF67	scaffold2.1507	228125	+	4	-1	26	GTGAGGGAGCAGATC-TGAGCGG	NA	NA	NA	
**Cas9-gRNA2:**	**Cas-OFFinder**											
1	CASgRNA2-OFF1	chr1	35799146	-	2	-1		ACGTGGGCGCAATCCTGCGCGG	TAGGCAGACAATGAGCCAAA	TAGATCGAGAAGGCGTCCTG	CCAAAACTAACCTGAGCGATCT	gRNA2-OFF17
2	CASgRNA2-OFF2	chr1	73355715	+	2	-1		ATGTGGGCGCAATCCTGCGTGG	NA	NA	NA	
3	CASgRNA2-OFF3	chr1	93320177	-	2	-1		ACGTGGGCGCAATCCTGTGCGG	NA	NA	NA	
4	CASgRNA2-OFF4	chr10	31243108	-	3			AAGTGGGCGCAGGTCCTCGGCGG	CCGCAGATAAGACACTGACG	GTGACGTGGAAGGAGAAGGA	CAAGACGACGTGTTCTGCTAA	gRNA2-OFF18
5	CASgRNA2-OFF5	chr1	73355694	-	2	-1		ATGTGGGCGCAATCCTGCGTGG	NA	NA	NA	
