# Supplementary material for: Activities and specificities of CRISPR/Cas9 and Cas12a nucleases for targeted mutagenesis in maize
Source: Plant Biotechnol J. 2018 Jul 22;17(2):362–72. doi: 10.1111/pbi.12982 (PMC6320322; doi:10.1111/pbi.12982)
Supplement: Supplementary file 2 — Data S1 Off‐target mutagenesis analysis. [file PBI-17-362-s002.pdf]

Data S1. Off-target mutagenesis analysis

A844B (gRNA1) T1 sample information

A total of 32 T1 plants derived from seven different T0 lines bearing on-target mutations were screened for off-target mutagenesis analysis. Twenty four plants were T-DNA positive (+), which constitutively expresses Cas9 nuclease, whereas eight were negative segregants (-). PCR was carried for each gDNA sample, and then four reactions were pooled (Sample pool) for Sanger sequencing. Thus, eight Sanger sequencing reactions were carried out per off-target. Fifteen CIRCLE-seq found (gRNA1-OFF1-10, 13-17) and two Cas-OFFinder predicted off-targets (gRNA1-OFF11-12) were analyzed, and there was no detectable level of mutations in 32 T1 lines. HM, homozygous; BI, biallelic; HT, heterozygous; MO, mosaic; WT, wild type; MT, *gl2* mutant.

T0 genotype		A844B	T-DNA	T1 phenotype	Sample pool	Off-targets with mutation
(-7, -7)	HM	1-4-1	-	MT	Pool 1	None
(-7, -7)	HM	1-4-2	-	WT	Pool 2	None
(-7, -7)	HM	1-4-3	-	WT	Pool 3	None
(-7, -7)	HM	1-4-4	-	WT	Pool 4	None
(-7, -7)	HM	1-4-5	+	MT	Pool 5	None
(-7, -7)	HM	1-4-6	+	MT	Pool 6	None
(-7, -7)	HM	1-4-7	+	MT	Pool 7	None
(-7, -7)	HM	1-4-8	+	MT	Pool 8	None
(+1, +1)	HM	2-5-1	-	WT	Pool 1	None
(+1, +1)	HM	2-5-2	+	WT	Pool 2	None
(+1, +1)	HM	2-5-3	+	WT	Pool 3	None
(+1, +1)	HM	2-5-4	-	WT	Pool 4	None
(-2, -2)	HM	3-2-1	+	MT	Pool 5	None
(-2, -2)	HM	3-2-2	+	MT	Pool 6	None
(-2, -2)	HM	3-2-3	+	MT	Pool 7	None
(-2, -2)	HM	3-2-4	+	MT	Pool 8	None
(-2, -2)	HM	3-4-1	+	MT	Pool 1	None
(-2, -2)	HM	3-4-2	+	MT	Pool 2	None
(-2, -2)	HM	3-4-3	+	MT	Pool 3	None
(-2, -2)	HM	3-4-4	+	MT	Pool 4	None
(0, +1)	HT	6-1-1	+	MT	Pool 5	None
(0, +1)	HT	6-1-2	-	MT	Pool 6	None
(0, +1)	HT	6-1-3	+	MT	Pool 7	None
(0, +1)	HT	6-1-4	-	MT	Pool 8	None
(0, +1)	HT	7-2-1	+	MT	Pool 1	None
(0, +1)	HT	7-2-2	+	WT	Pool 2	None
(0, +1)	HT	7-2-3	+	MT	Pool 3	None
(0, +1)	HT	7-2-4	+	MT	Pool 4	None
(+1, -2)	BI	8-2-1	+	MT	Pool 5	None
(+1, -2)	BI	8-2-2	+	MT	Pool 6	None
(+1, -2)	BI	8-2-3	+	MT	Pool 7	None
(+1, -2)	BI	8-2-4	+	MT	Pool 8	None

gRNA1-OFF1

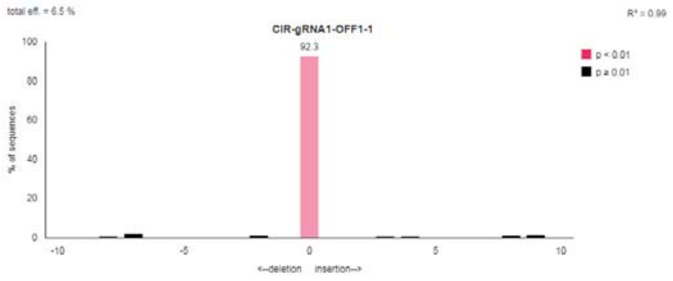
total eff. = 6.5 %
R² = 0.99
CIR-gRNA1-OFF1-1
92.3
p < 0.01
p ≥ 0.01
% of sequences
<--deletion insertion-->

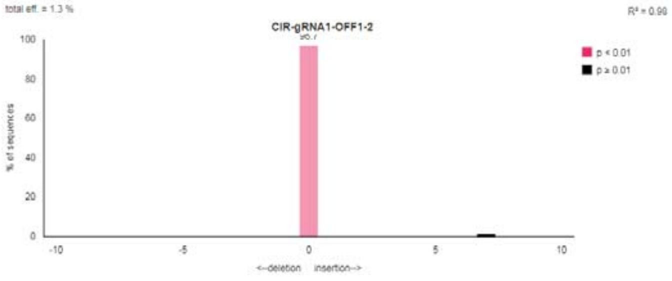
total eff. = 1.3 %
CIR-gRNA1-OFF1-2
p < 0.01
p ≥ 0.01
% of sequences
<--deletion insertion-->

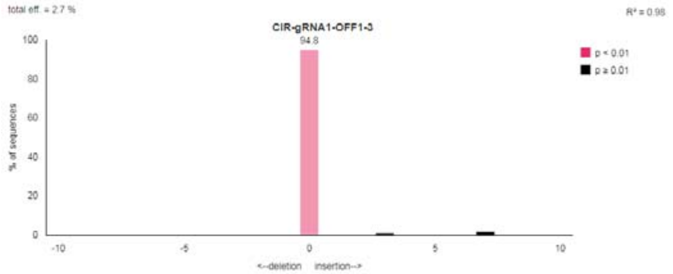
total eff. = 2.7 %
R² = 0.98
CIR-gRNA1-OFF1-3
94.8
p < 0.01
p ≥ 0.01
% of sequences
<--deletion insertion-->

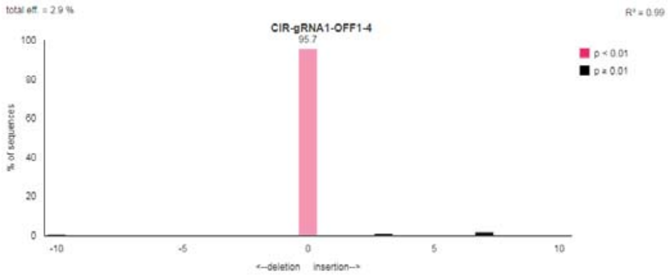
total eff. = 2.9 %
R² = 0.99
CIR-gRNA1-OFF1-4
95.7
p < 0.01
p ≥ 0.01
% of sequences
<--deletion insertion-->

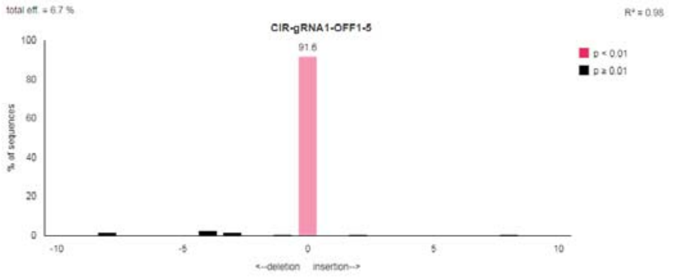
total eff. = 6.7 %
R² = 0.98
CIR-gRNA1-OFF1-5
91.6
p < 0.01
p ≥ 0.01
% of sequences
<--deletion insertion-->

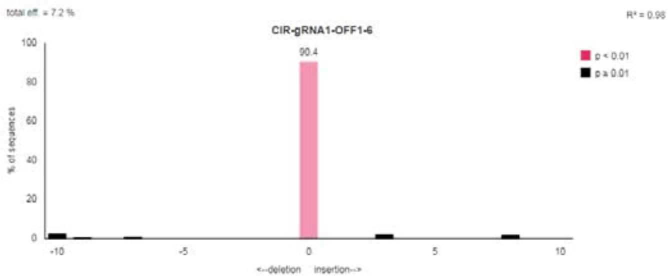
total eff. = 7.2 %
R² = 0.98
CIR-gRNA1-OFF1-6
90.4
p < 0.01
p ≥ 0.01
% of sequences
<--deletion insertion-->

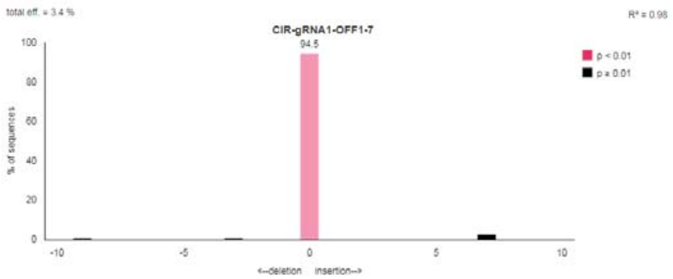
total eff. = 3.4 %
R² = 0.98
CIR-gRNA1-OFF1-7
94.5
p < 0.01
p ≥ 0.01
% of sequences
<--deletion insertion-->

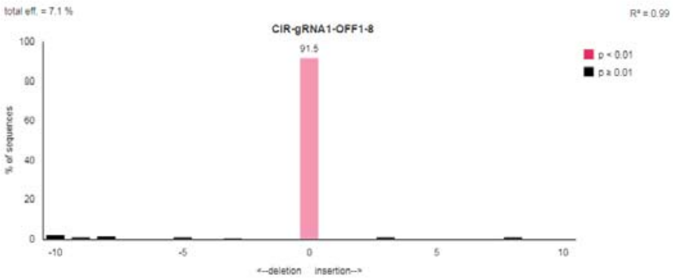
total eff. = 7.1 %
R² = 0.99
CIR-gRNA1-OFF1-8
91.5
p < 0.01
p ≥ 0.01
% of sequences
<--deletion insertion-->

gRNA1-OFF2

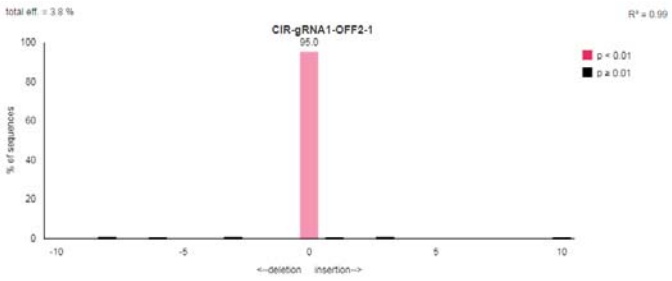

total eff. = 3.8 %
CIR-gRNA1-OFF2-1
95.0
R² = 0.99
p < 0.01
p ≥ 0.01
% of sequences
<--deletion insertion-->

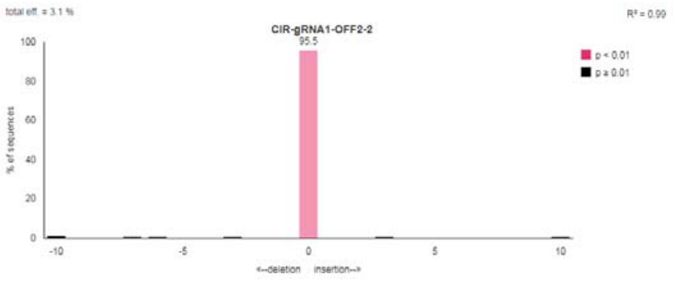

total eff. = 3.1 %
CIR-gRNA1-OFF2-2
95.5
R² = 0.99
p < 0.01
p ≥ 0.01
% of sequences
<--deletion insertion-->

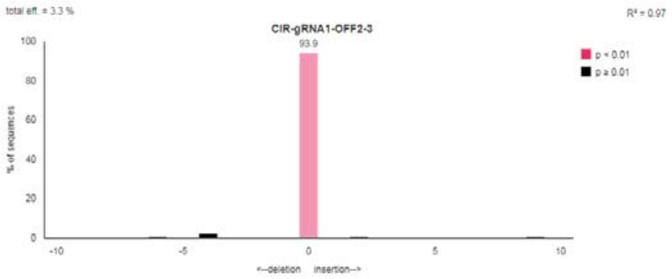

total eff. = 3.3 %
CIR-gRNA1-OFF2-3
93.9
R² = 0.97
p < 0.01
p ≥ 0.01
% of sequences
<--deletion insertion-->

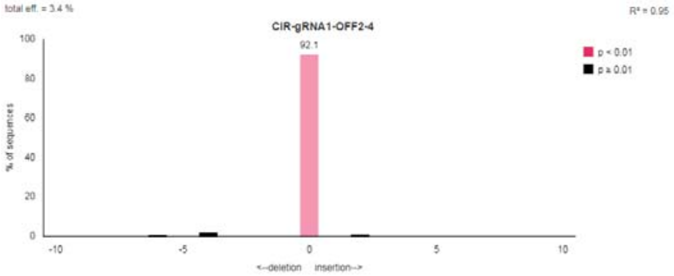

total eff. = 3.4 %
CIR-gRNA1-OFF2-4
92.1
R² = 0.95
p < 0.01
p ≥ 0.01
% of sequences
<--deletion insertion-->

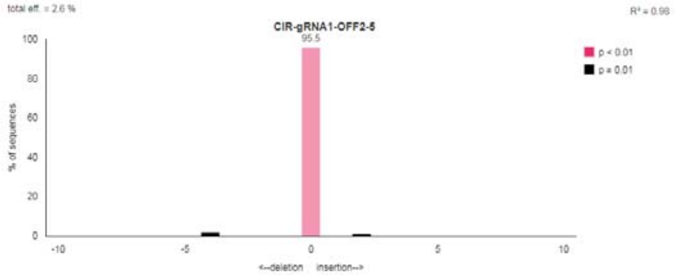

total eff. = 2.6 %
CIR-gRNA1-OFF2-5
95.5
R² = 0.98
p < 0.01
p ≥ 0.01
% of sequences
<--deletion insertion-->

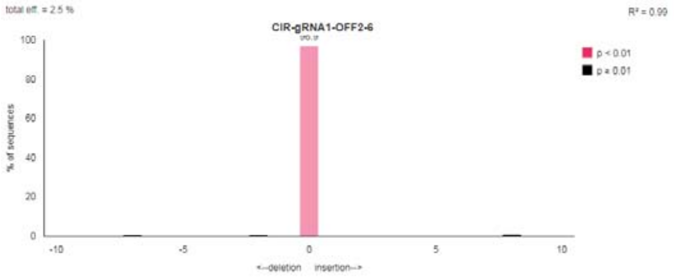

total eff. = 2.5 %
CIR-gRNA1-OFF2-6
R² = 0.99
p < 0.01
p ≥ 0.01
% of sequences
<--deletion insertion-->

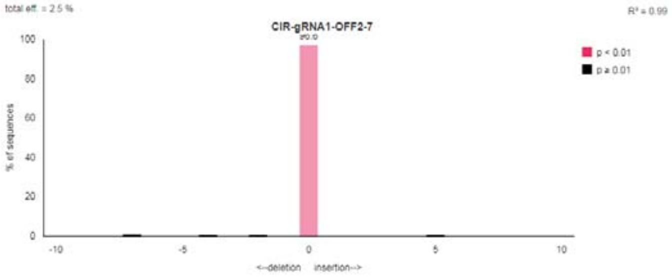

total eff. = 2.5 %
CIR-gRNA1-OFF2-7
R² = 0.99
p < 0.01
p ≥ 0.01
% of sequences
<--deletion insertion-->

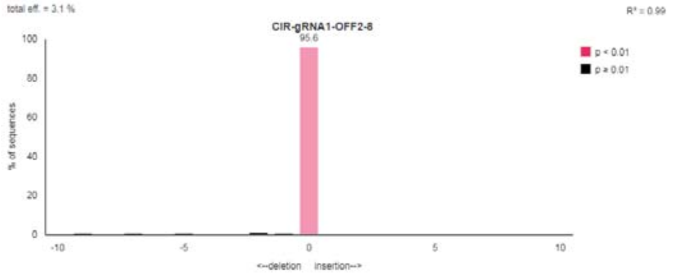

total eff. = 3.1 %
CIR-gRNA1-OFF2-8
95.6
R² = 0.99
p < 0.01
p ≥ 0.01
% of sequences
<--deletion insertion-->

gRNA1-OFF3

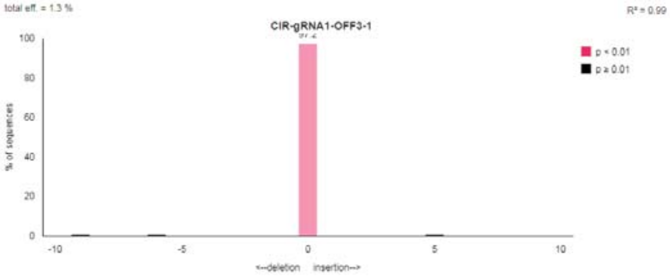
total eff. = 1.3 %
R² = 0.99
CIR-gRNA1-OFF3-1
p < 0.01
p ≥ 0.01
% of sequences
<--deletion   insertion-->

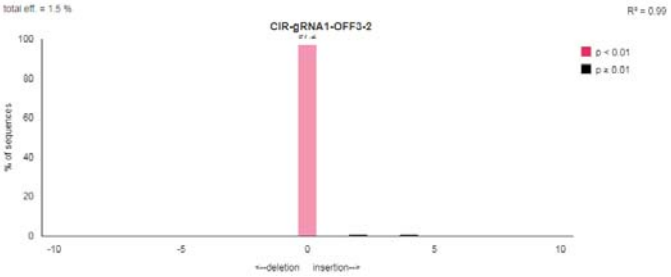
total eff. = 1.5 %
R² = 0.99
CIR-gRNA1-OFF3-2
p < 0.01
p ≥ 0.01
% of sequences
<--deletion   insertion-->

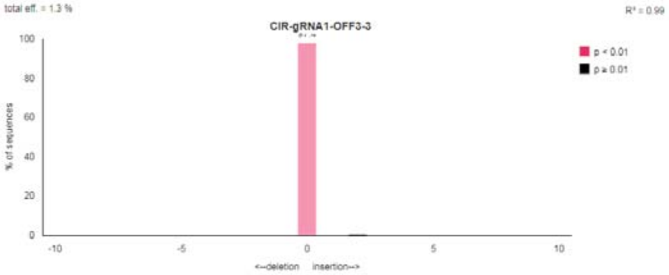
total eff. = 1.3 %
R² = 0.99
CIR-gRNA1-OFF3-3
p < 0.01
p ≥ 0.01
% of sequences
<--deletion   insertion-->

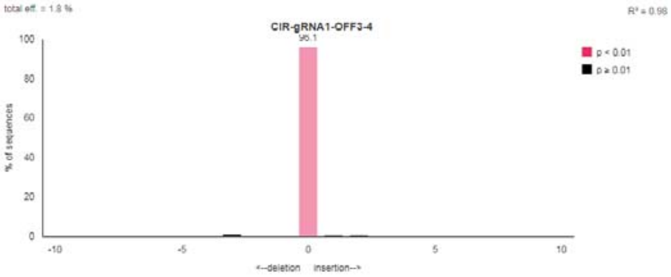
total eff. = 1.8 %
R² = 0.98
CIR-gRNA1-OFF3-4
96.1
p < 0.01
p ≥ 0.01
% of sequences
<--deletion   insertion-->

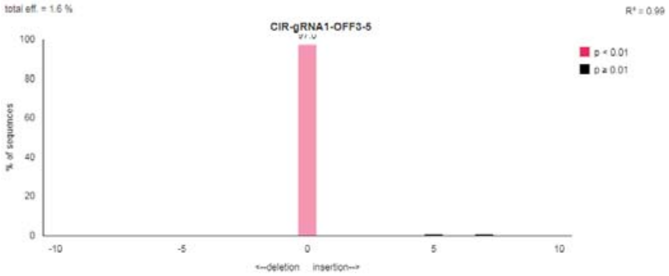
total eff. = 1.6 %
R² = 0.99
CIR-gRNA1-OFF3-5
p < 0.01
p ≥ 0.01
% of sequences
<--deletion   insertion-->

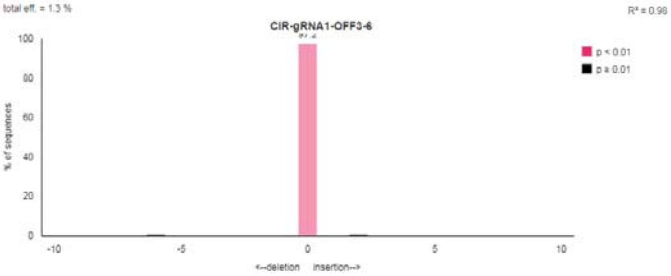
total eff. = 1.3 %
R² = 0.98
CIR-gRNA1-OFF3-6
p < 0.01
p ≥ 0.01
% of sequences
<--deletion   insertion-->

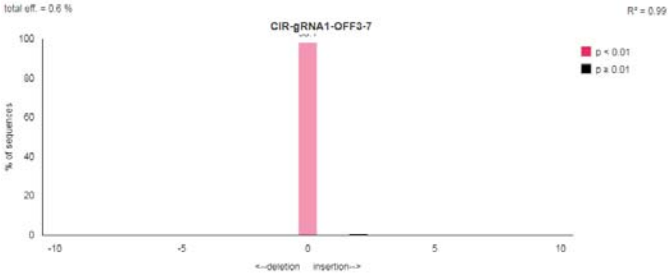
total eff. = 0.6 %
R² = 0.99
CIR-gRNA1-OFF3-7
p < 0.01
p ≥ 0.01
% of sequences
<--deletion   insertion-->

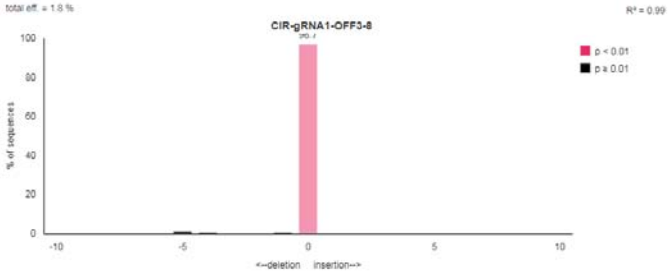
total eff. = 1.8 %
R² = 0.99
CIR-gRNA1-OFF3-8
p < 0.01
p ≥ 0.01
% of sequences
<--deletion   insertion-->

gRNA1-OFF4

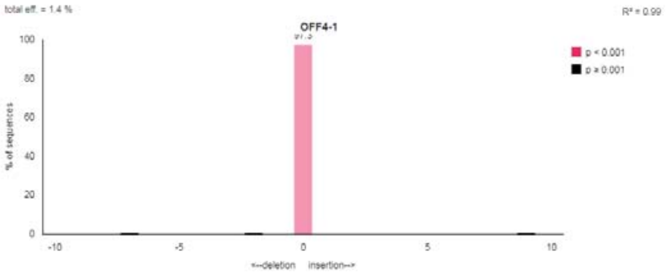
total eff. = 1.4 %
R² = 0.99
OFF4-1
% of sequences
p < 0.001
p ≥ 0.001
<--deletion insertion-->

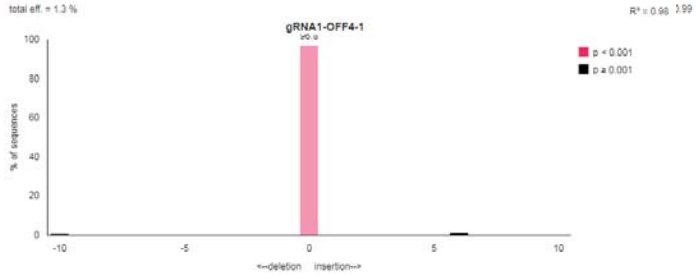
total eff. = 1.3 %
R² = 0.98
gRNA1-OFF4-1
% of sequences
p < 0.001
p ≥ 0.001
<--deletion insertion-->

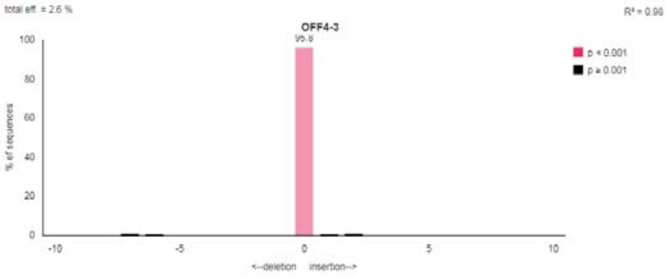
total eff. = 2.6 %
R² = 0.98
OFF4-3
% of sequences
p < 0.001
p ≥ 0.001
<--deletion insertion-->

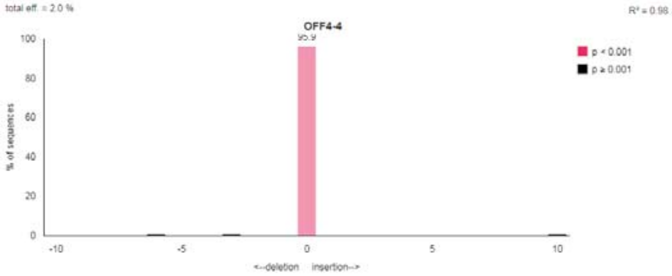
total eff. = 2.0 %
R² = 0.98
OFF4-4
% of sequences
p < 0.001
p ≥ 0.001
<--deletion insertion-->

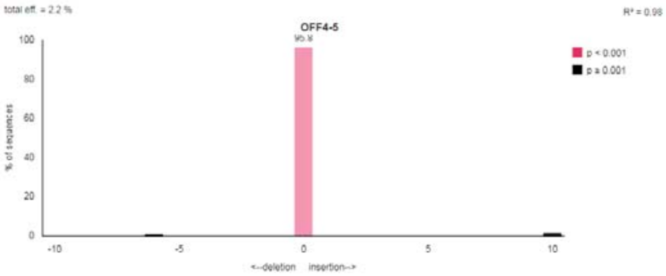
total eff. = 2.2 %
R² = 0.98
OFF4-5
% of sequences
p < 0.001
p ≥ 0.001
<--deletion insertion-->

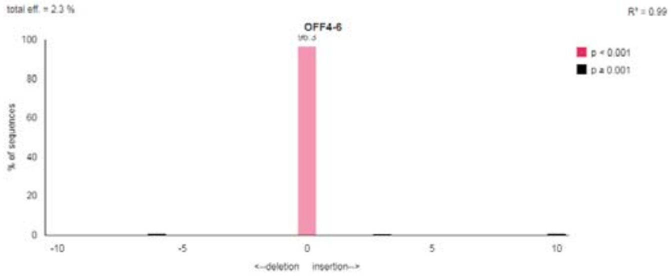
total eff. = 2.3 %
R² = 0.99
OFF4-6
% of sequences
p < 0.001
p ≥ 0.001
<--deletion insertion-->

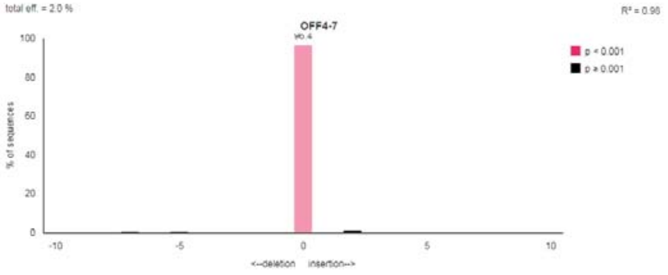
total eff. = 2.0 %
R² = 0.98
OFF4-7
% of sequences
p < 0.001
p ≥ 0.001
<--deletion insertion-->

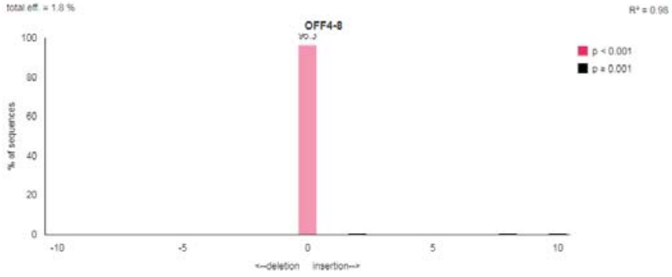
total eff. = 1.8 %
R² = 0.98
OFF4-8
% of sequences
p < 0.001
p ≥ 0.001
<--deletion insertion-->

gRNA1-OFF5

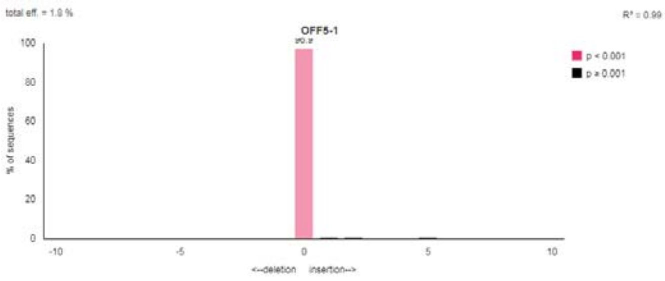
total eff. = 1.9 %
R² = 0.99
OFF5-1
p < 0.001
p ≥ 0.001
% of sequences
<--deletion insertion-->

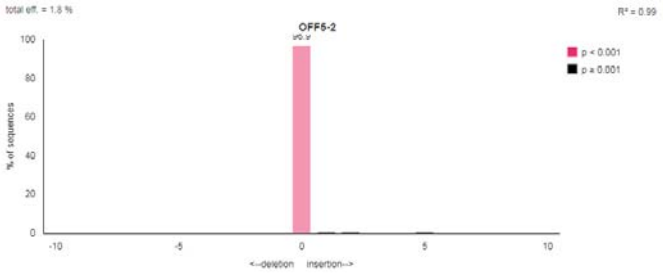
total eff. = 1.8 %
R² = 0.99
OFF5-2
p < 0.001
p ≥ 0.001
% of sequences
<--deletion insertion-->

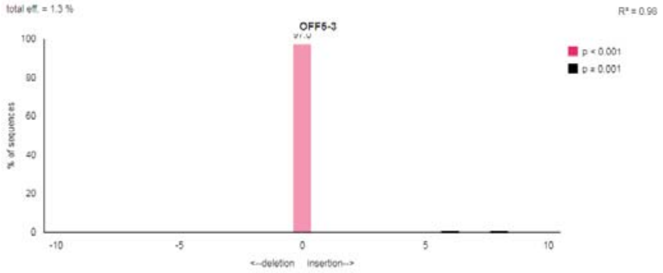
total eff. = 1.3 %
R² = 0.98
OFF5-3
p < 0.001
p ≥ 0.001
% of sequences
<--deletion insertion-->

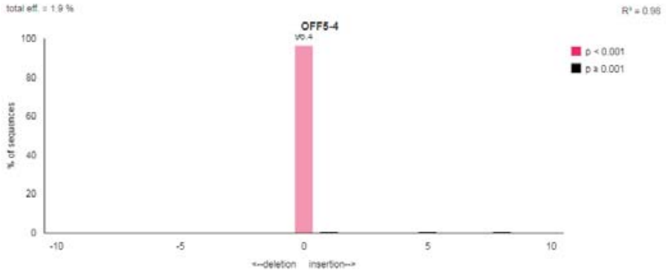
total eff. = 1.9 %
R² = 0.98
OFF5-4
p < 0.001
p ≥ 0.001
% of sequences
<--deletion insertion-->

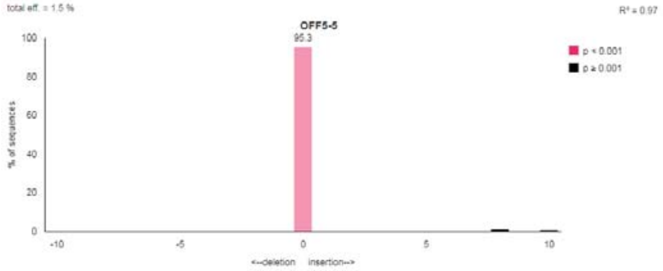
total eff. = 1.5 %
R² = 0.97
OFF5-5
95.3
p < 0.001
p ≥ 0.001
% of sequences
<--deletion insertion-->

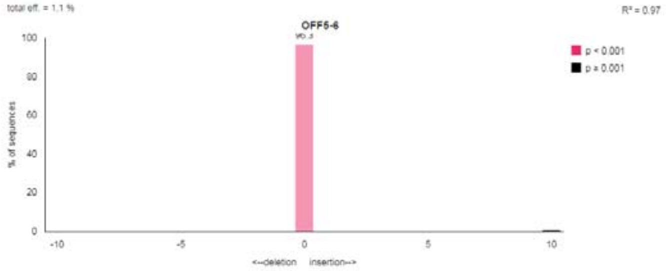
total eff. = 1.1 %
R² = 0.97
OFF5-6
p < 0.001
p ≥ 0.001
% of sequences
<--deletion insertion-->

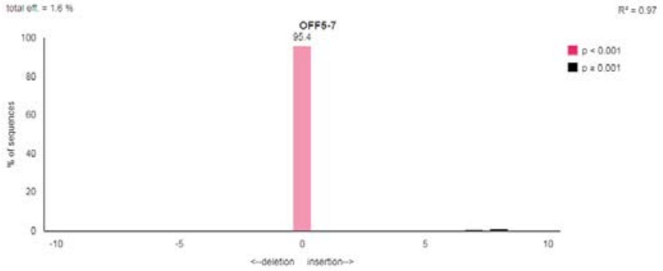
total eff. = 1.6 %
R² = 0.97
OFF5-7
95.4
p < 0.001
p ≥ 0.001
% of sequences
<--deletion insertion-->

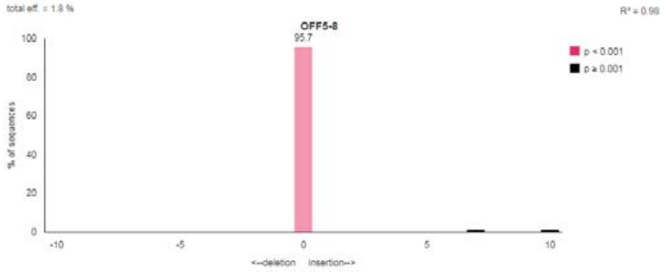
total eff. = 1.8 %
R² = 0.98
OFF5-8
95.7
p < 0.001
p ≥ 0.001
% of sequences
<--deletion insertion-->

gRNA1-OFF6

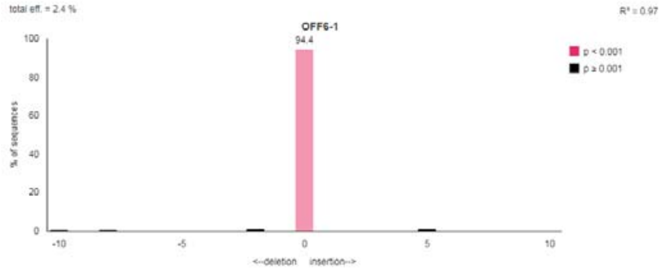

total eff. = 2.4 %
R² = 0.97
OFF6-1
94.4
p < 0.001
p ≥ 0.001
% of sequences
<--deletion insertion-->

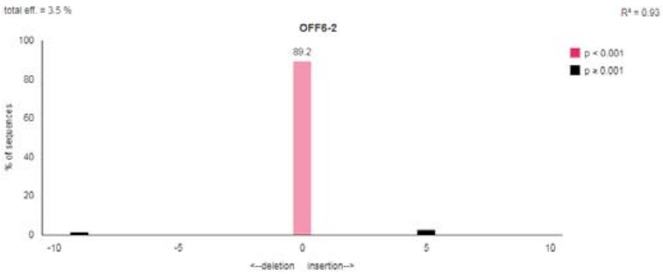

total eff. = 3.5 %
R² = 0.93
OFF6-2
89.2
p < 0.001
p ≥ 0.001
% of sequences
<--deletion insertion-->

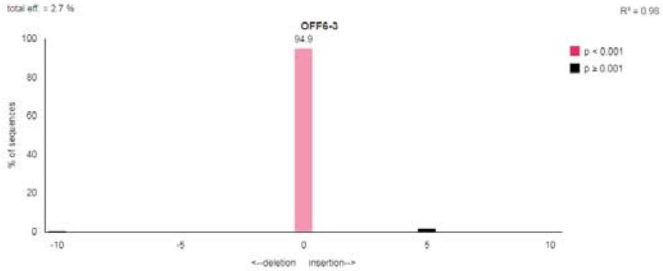

total eff. = 2.7 %
R² = 0.98
OFF6-3
94.9
p < 0.001
p ≥ 0.001
% of sequences
<--deletion insertion-->

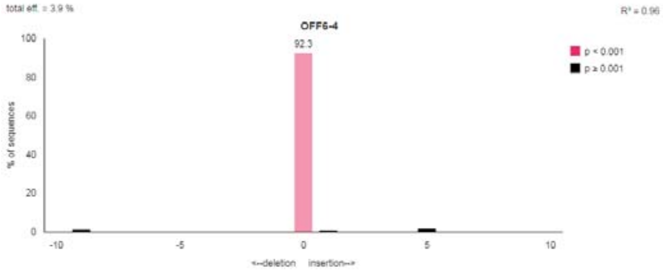

total eff. = 3.9 %
R² = 0.96
OFF6-4
92.3
p < 0.001
p ≥ 0.001
% of sequences
<--deletion insertion-->

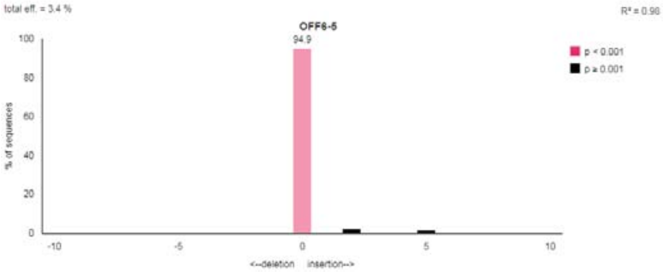

total eff. = 3.4 %
R² = 0.98
OFF6-5
94.9
p < 0.001
p ≥ 0.001
% of sequences
<--deletion insertion-->

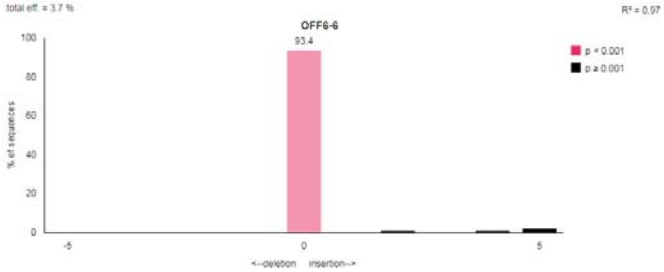

total eff. = 3.7 %
R² = 0.97
OFF6-6
93.4
p < 0.001
p ≥ 0.001
% of sequences
<--deletion insertion-->

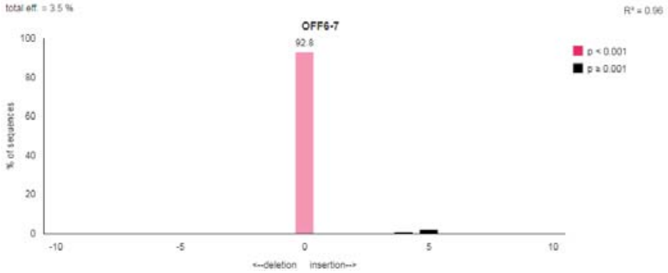

total eff. = 3.5 %
R² = 0.96
OFF6-7
92.8
p < 0.001
p ≥ 0.001
% of sequences
<--deletion insertion-->

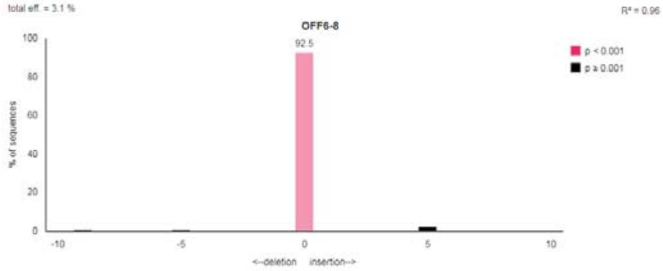

total eff. = 3.1 %
R² = 0.96
OFF6-8
92.5
p < 0.001
p ≥ 0.001
% of sequences
<--deletion insertion-->

gRNA1-OFF7

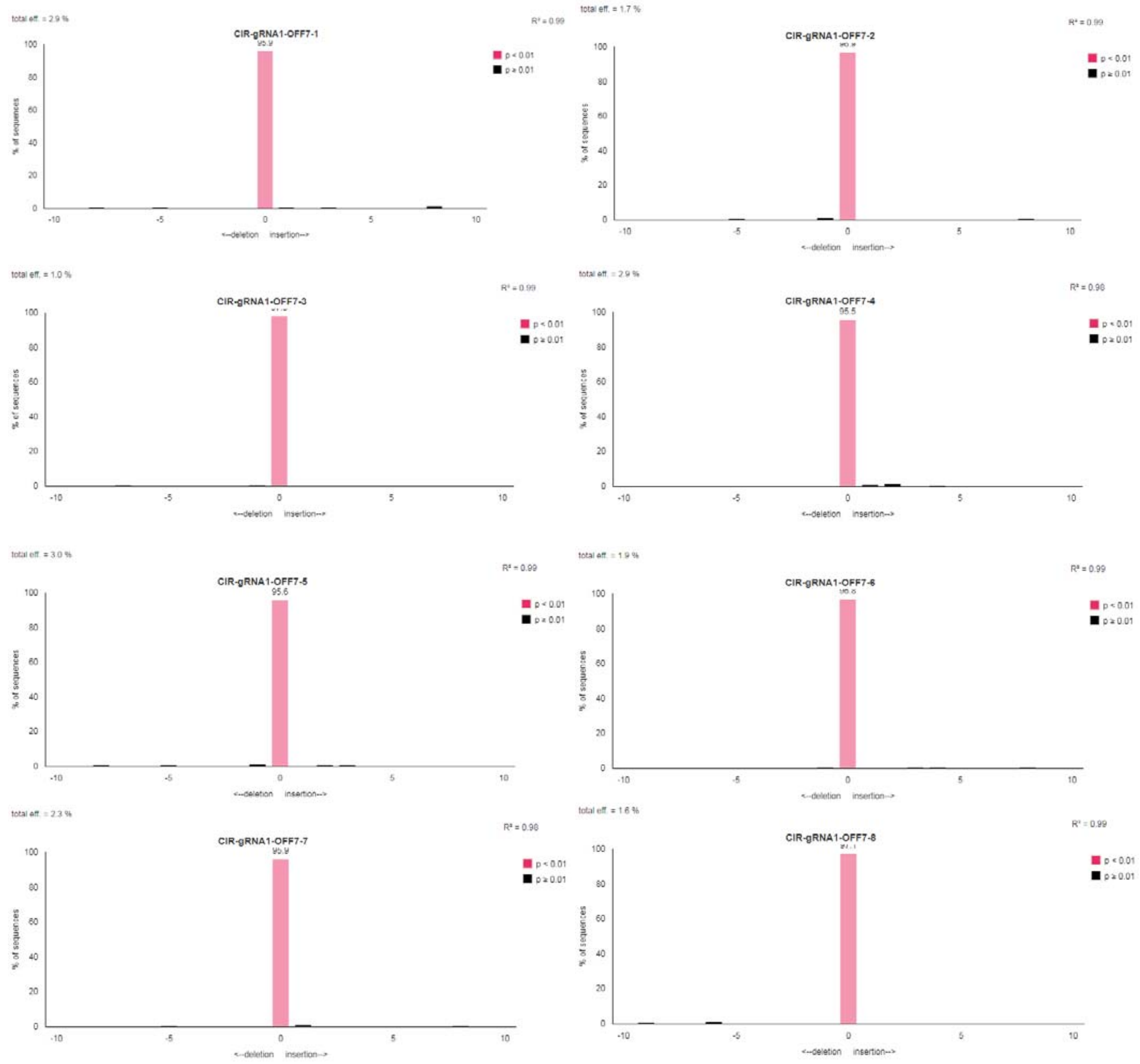
total eff. = 2.9 %
R² = 0.99
CIR-gRNA1-OFF7-1
p < 0.01
p ≥ 0.01
% of sequences
<--deletion insertion-->
total eff. = 1.7 %
R² = 0.99
CIR-gRNA1-OFF7-2
total eff. = 1.0 %
R² = 0.99
CIR-gRNA1-OFF7-3
total eff. = 2.9 %
R² = 0.98
CIR-gRNA1-OFF7-4
95.5
total eff. = 3.0 %
R² = 0.99
CIR-gRNA1-OFF7-5
95.6
total eff. = 1.9 %
R² = 0.99
CIR-gRNA1-OFF7-6
total eff. = 2.3 %
R² = 0.98
CIR-gRNA1-OFF7-7
total eff. = 1.6 %
R² = 0.99
CIR-gRNA1-OFF7-8

gRNA1-OFF8

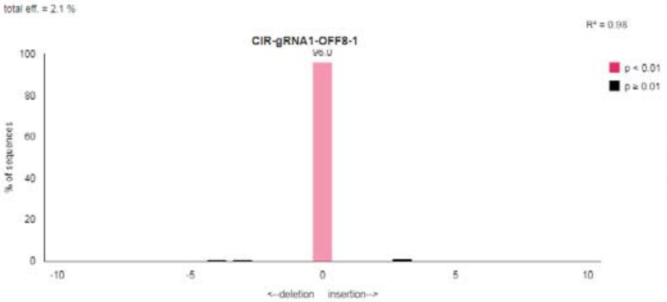
total eff. = 2.1 %
R² = 0.98
CIR-gRNA1-OFF8-1
p < 0.01
p ≥ 0.01
% of sequences
<--deletion insertion-->

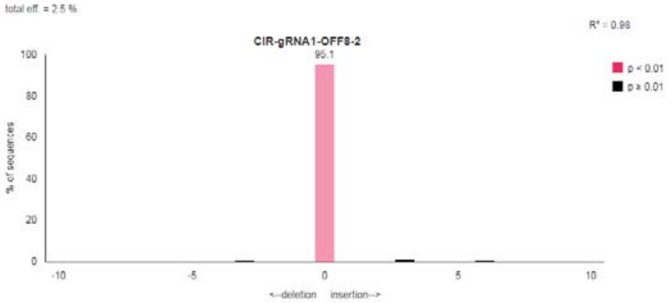
total eff. = 2.5 %
R² = 0.98
CIR-gRNA1-OFF8-2
95.1
p < 0.01
p ≥ 0.01
% of sequences
<--deletion insertion-->

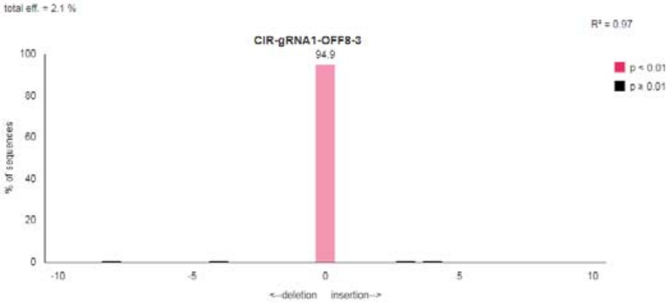
total eff. = 2.1 %
R² = 0.97
CIR-gRNA1-OFF8-3
94.9
p < 0.01
p ≥ 0.01
% of sequences
<--deletion insertion-->

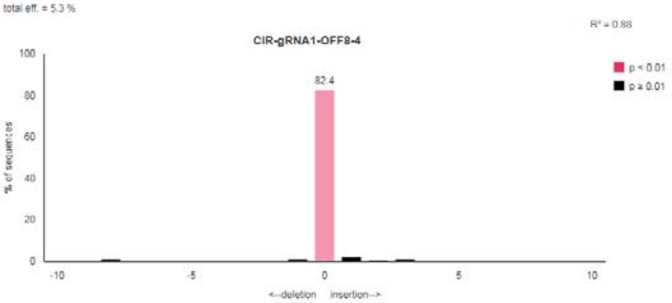
total eff. = 5.3 %
R² = 0.88
CIR-gRNA1-OFF8-4
82.4
p < 0.01
p ≥ 0.01
% of sequences
<--deletion insertion-->

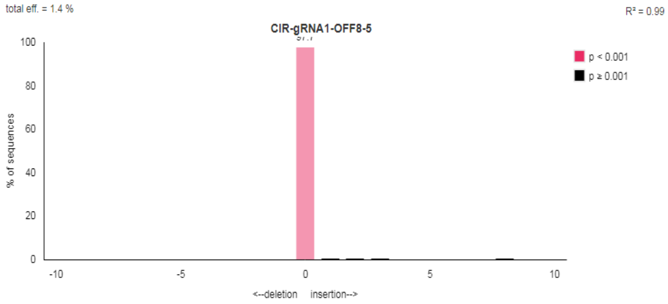
total eff. = 1.4 %
R² = 0.99
CIR-gRNA1-OFF8-5
p < 0.001
p ≥ 0.001
% of sequences
<--deletion insertion-->

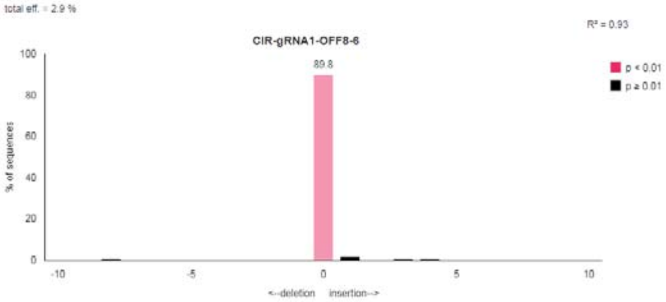
total eff. = 2.9 %
R² = 0.93
CIR-gRNA1-OFF8-6
89.8
p < 0.01
p ≥ 0.01
% of sequences
<--deletion insertion-->

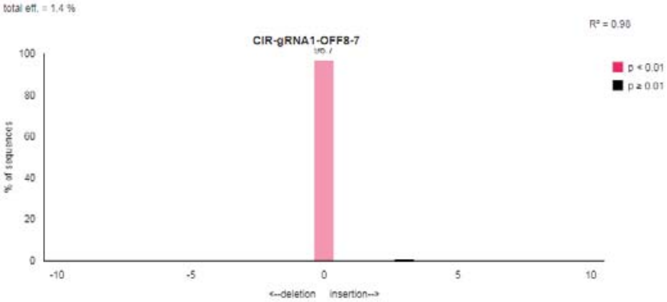
total eff. = 1.4 %
R² = 0.98
CIR-gRNA1-OFF8-7
p < 0.01
p ≥ 0.01
% of sequences
<--deletion insertion-->

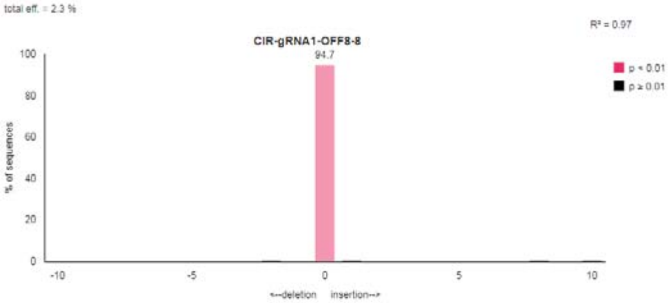
total eff. = 2.3 %
R² = 0.97
CIR-gRNA1-OFF8-8
94.7
p < 0.01
p ≥ 0.01
% of sequences
<--deletion insertion-->

gRNA1-OFF9

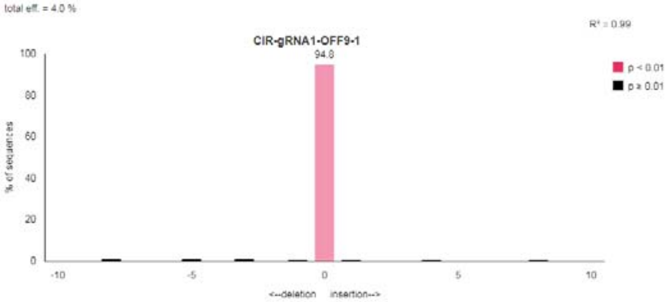

total eff. = 4.0 %
R² = 0.99
CIR-gRNA1-OFF9-1
94.8
p < 0.01
p ≥ 0.01
% of sequences
<--deletion insertion-->

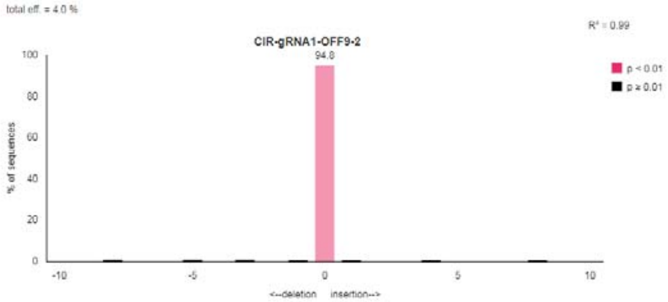

total eff. = 4.0 %
R² = 0.99
CIR-gRNA1-OFF9-2
94.8
p < 0.01
p ≥ 0.01
% of sequences
<--deletion insertion-->

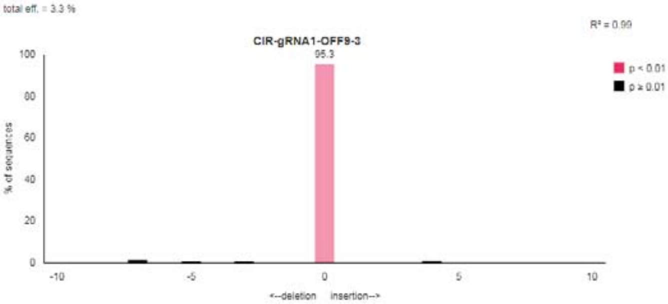

total eff. = 3.3 %
R² = 0.99
CIR-gRNA1-OFF9-3
95.3
p < 0.01
p ≥ 0.01
% of sequences
<--deletion insertion-->

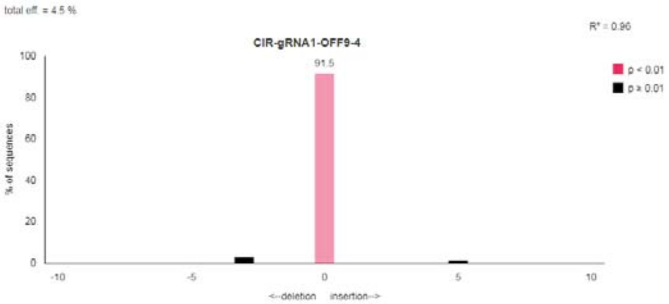

total eff. = 4.5 %
R² = 0.96
CIR-gRNA1-OFF9-4
91.5
p < 0.01
p ≥ 0.01
% of sequences
<--deletion insertion-->

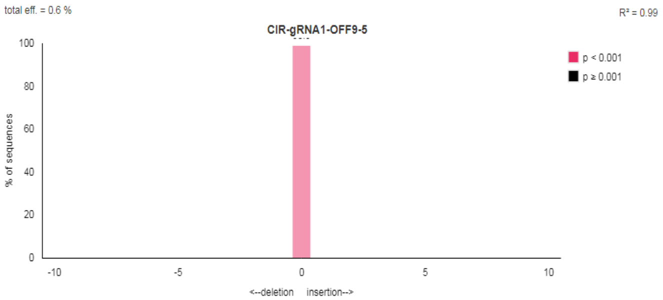

total eff. = 0.6 %
R² = 0.99
CIR-gRNA1-OFF9-5
p < 0.001
p ≥ 0.001
% of sequences
<--deletion insertion-->

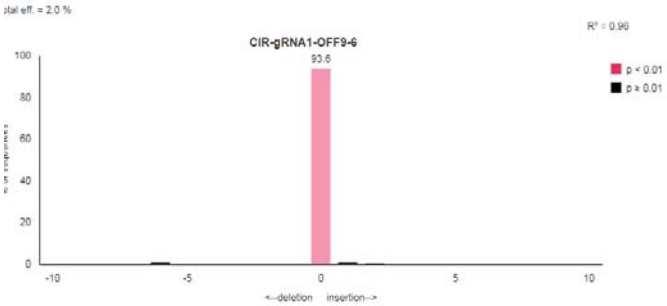

otal eff. = 2.0 %
R² = 0.96
CIR-gRNA1-OFF9-6
93.6
p < 0.01
p ≥ 0.01
<--deletion insertion-->

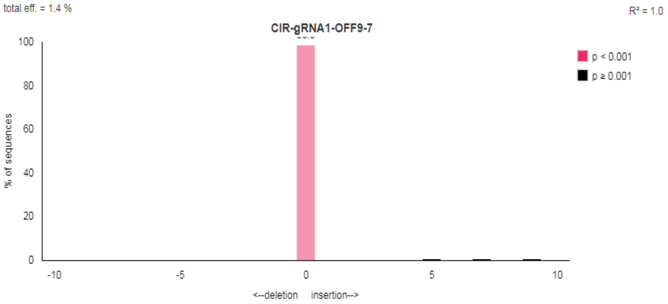

total eff. = 1.4 %
R² = 1.0
CIR-gRNA1-OFF9-7
p < 0.001
p ≥ 0.001
% of sequences
<--deletion insertion-->

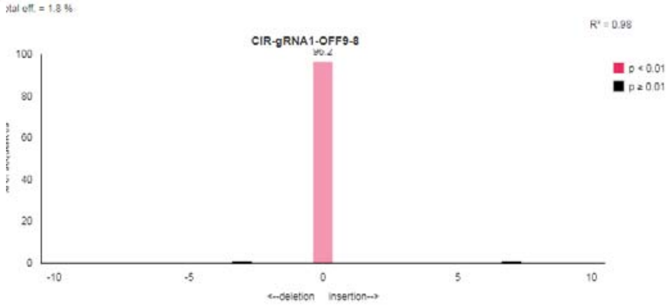

otal eff. = 1.8 %
R² = 0.98
CIR-gRNA1-OFF9-8
p < 0.01
p ≥ 0.01
<--deletion insertion-->

gRNA1-OFF10

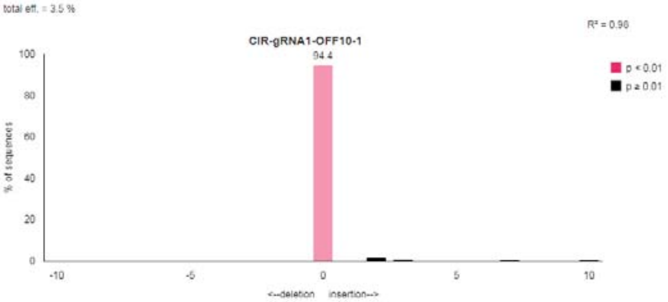

total eff. = 3.5 %
R² = 0.98
CIR-gRNA1-OFF10-1
94.4
p < 0.01
p ≥ 0.01
% of sequences
<--deletion insertion-->

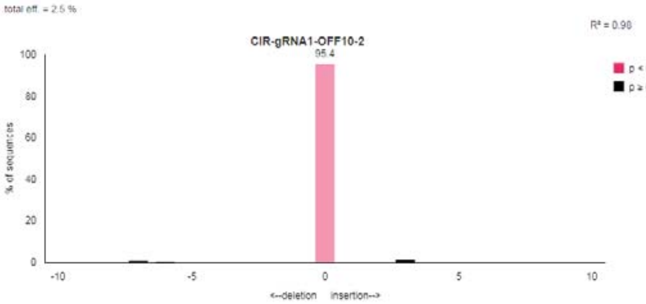

total eff. = 2.5 %
R² = 0.98
CIR-gRNA1-OFF10-2
95.4
p < 0.01
p ≥ 0.01
% of sequences
<--deletion insertion-->

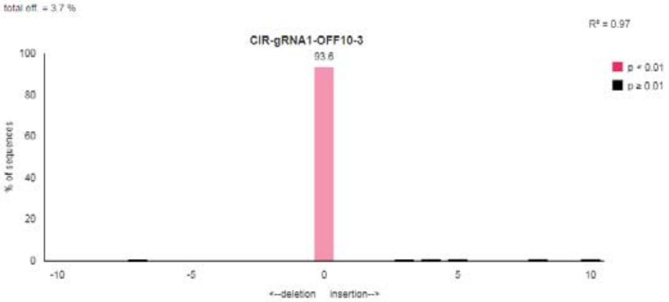

total eff. = 3.7 %
R² = 0.97
CIR-gRNA1-OFF10-3
93.6
p < 0.01
p ≥ 0.01
% of sequences
<--deletion insertion-->

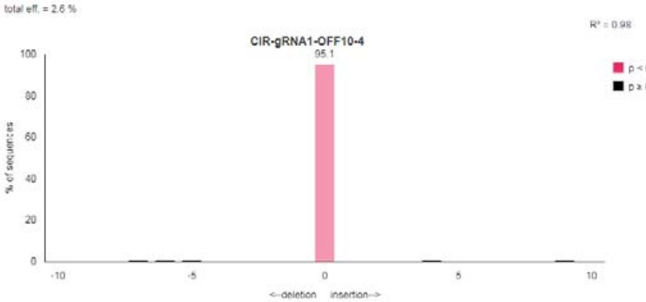

total eff. = 2.6 %
R² = 0.98
CIR-gRNA1-OFF10-4
95.1
p < 0.01
p ≥ 0.01
% of sequences
<--deletion insertion-->

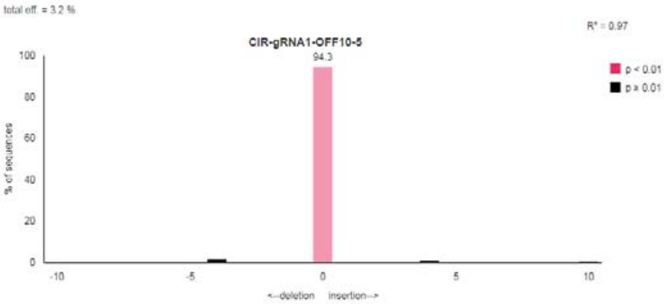

total eff. = 3.2 %
R² = 0.97
CIR-gRNA1-OFF10-5
94.3
p < 0.01
p ≥ 0.01
% of sequences
<--deletion insertion-->

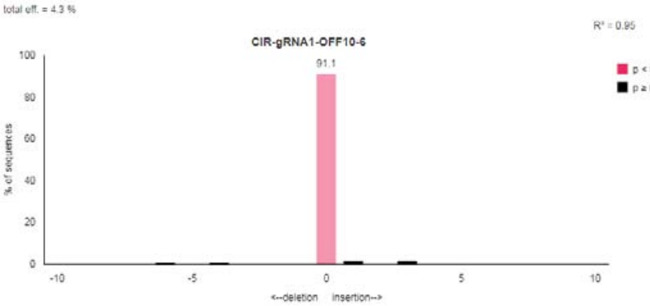

total eff. = 4.3 %
R² = 0.95
CIR-gRNA1-OFF10-6
91.1
p < 0.01
p ≥ 0.01
% of sequences
<--deletion insertion-->

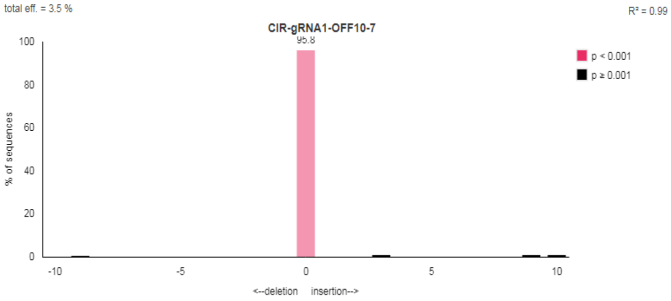

total eff. = 3.5 %
R² = 0.99
CIR-gRNA1-OFF10-7
95.8
p < 0.001
p ≥ 0.001
% of sequences
<--deletion insertion-->

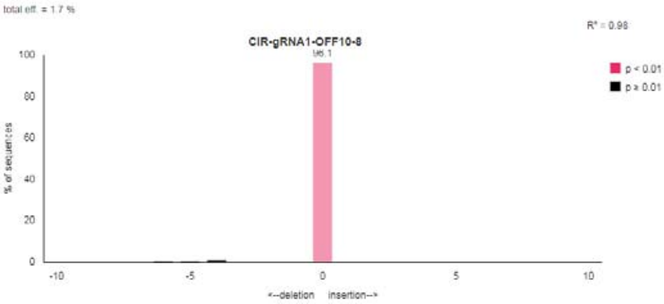

total eff. = 1.7 %
R² = 0.98
CIR-gRNA1-OFF10-8
p < 0.01
p ≥ 0.01
% of sequences
<--deletion insertion-->

gRNA1-OFF11

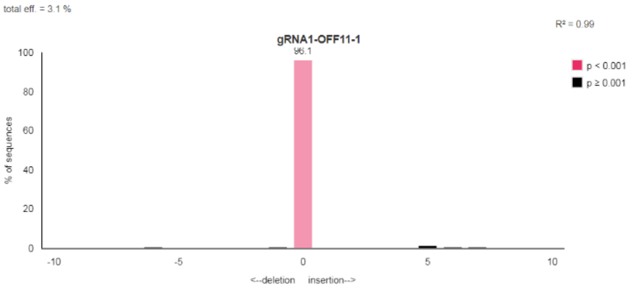
total eff. = 3.1 %
R² = 0.99
gRNA1-OFF11-1
96.1
p < 0.001
p ≥ 0.001
% of sequences
<--deletion insertion-->

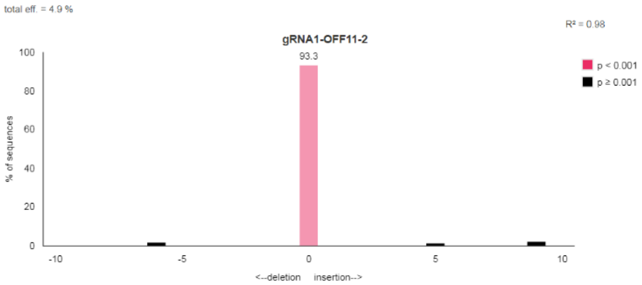
total eff. = 4.9 %
R² = 0.98
gRNA1-OFF11-2
93.3
p < 0.001
p ≥ 0.001
% of sequences
<--deletion insertion-->

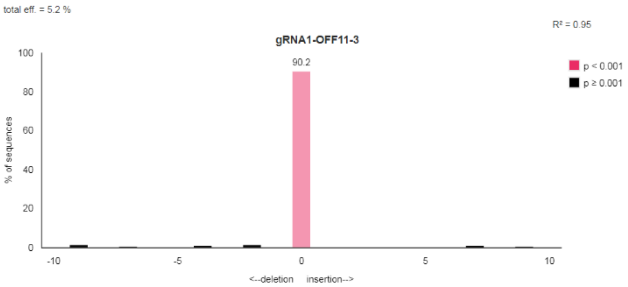
total eff. = 5.2 %
R² = 0.95
gRNA1-OFF11-3
90.2
p < 0.001
p ≥ 0.001
% of sequences
<--deletion insertion-->

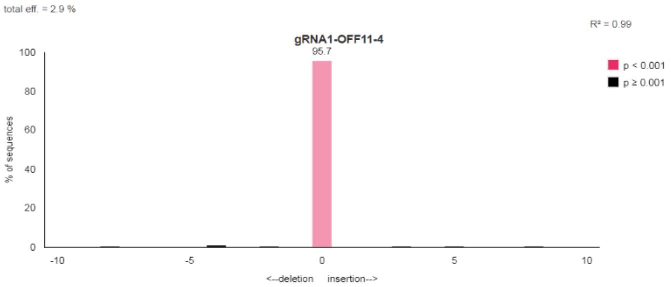
total eff. = 2.9 %
R² = 0.99
gRNA1-OFF11-4
95.7
p < 0.001
p ≥ 0.001
% of sequences
<--deletion insertion-->

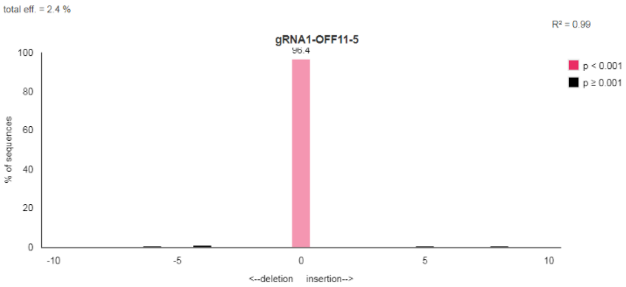
total eff. = 2.4 %
R² = 0.99
gRNA1-OFF11-5
96.4
p < 0.001
p ≥ 0.001
% of sequences
<--deletion insertion-->

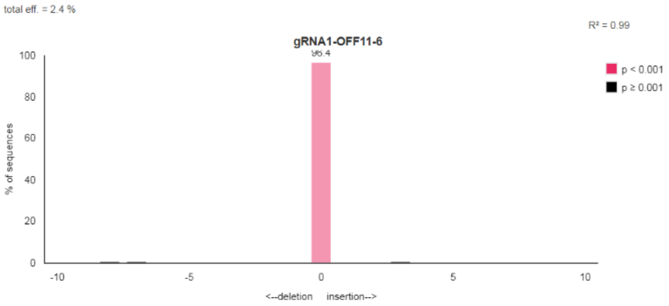
total eff. = 2.4 %
R² = 0.99
gRNA1-OFF11-6
96.4
p < 0.001
p ≥ 0.001
% of sequences
<--deletion insertion-->

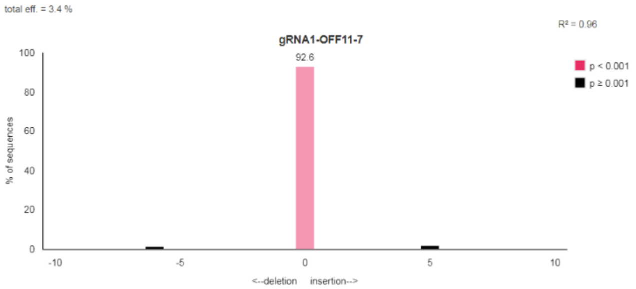
total eff. = 3.4 %
R² = 0.96
gRNA1-OFF11-7
92.6
p < 0.001
p ≥ 0.001
% of sequences
<--deletion insertion-->

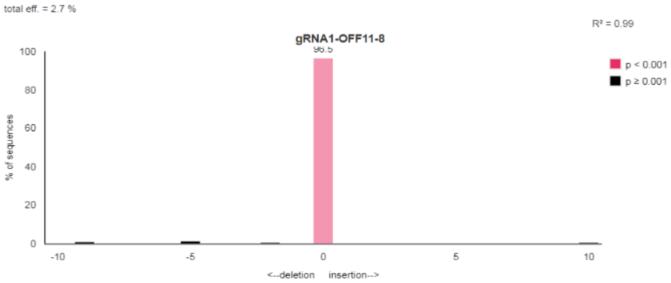
total eff. = 2.7 %
R² = 0.99
gRNA1-OFF11-8
p < 0.001
p ≥ 0.001
% of sequences
<--deletion insertion-->

gRNA1-OFF12

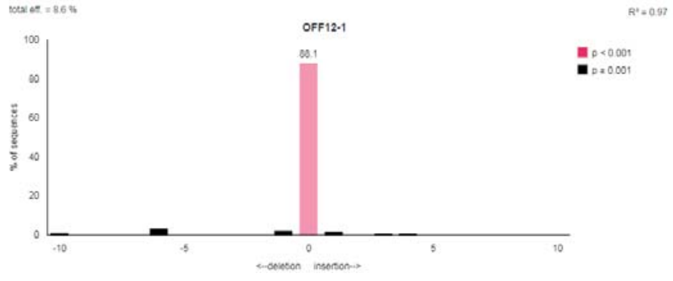
total eff. = 8.6 %
R² = 0.97
OFF12-1
88.1
p < 0.001
p ≥ 0.001
% of sequences
<--deletion insertion-->

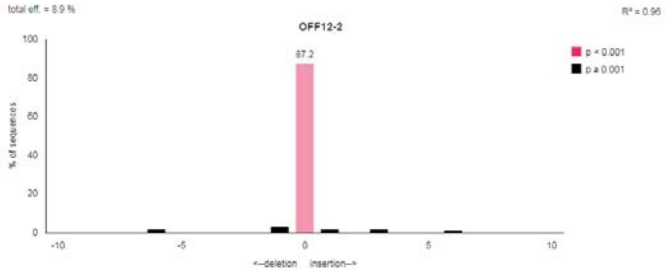
total eff. = 8.9 %
R² = 0.96
OFF12-2
87.2
p < 0.001
p ≥ 0.001
% of sequences
<--deletion insertion-->

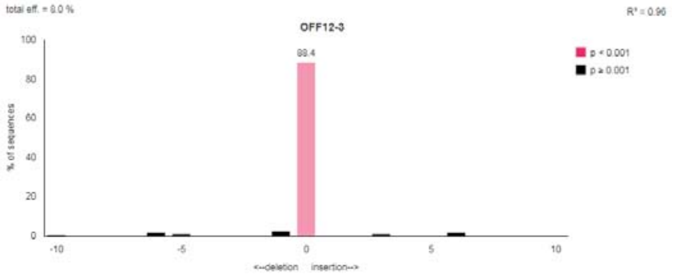
total eff. = 8.0 %
R² = 0.96
OFF12-3
88.4
p < 0.001
p ≥ 0.001
% of sequences
<--deletion insertion-->

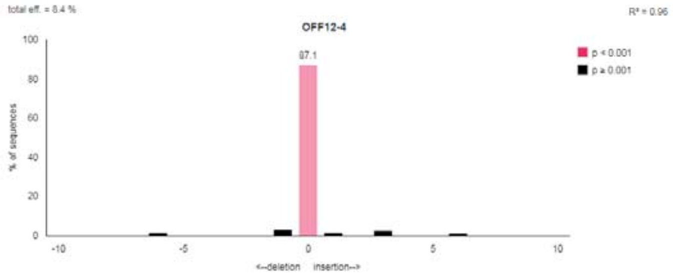
total eff. = 8.4 %
R² = 0.96
OFF12-4
87.1
p < 0.001
p ≥ 0.001
% of sequences
<--deletion insertion-->

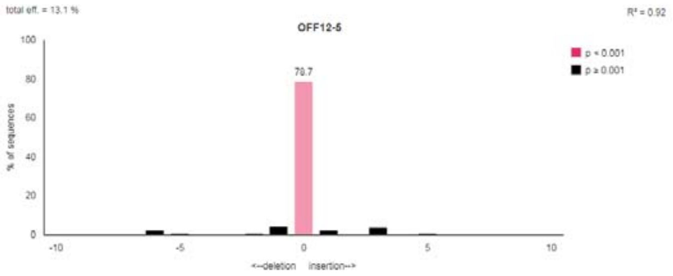
total eff. = 13.1 %
R² = 0.92
OFF12-5
78.7
p < 0.001
p ≥ 0.001
% of sequences
<--deletion insertion-->

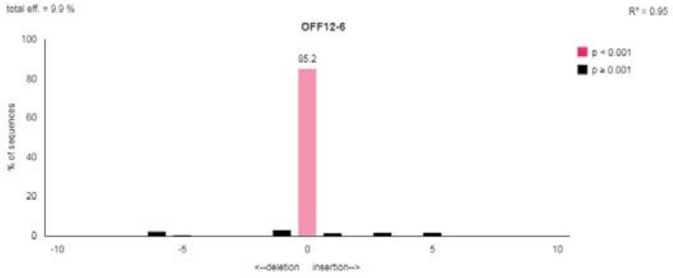
total eff. = 9.9 %
R² = 0.95
OFF12-6
85.2
p < 0.001
p ≥ 0.001
% of sequences
<--deletion insertion-->

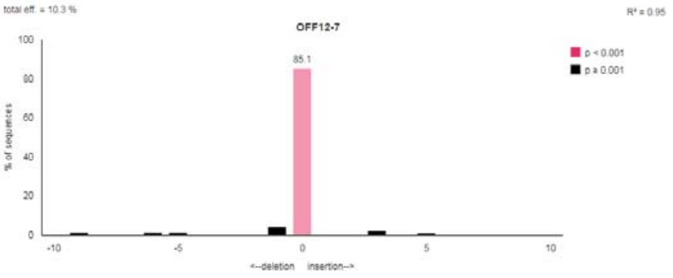
total eff. = 10.3 %
R² = 0.95
OFF12-7
85.1
p < 0.001
p ≥ 0.001
% of sequences
<--deletion insertion-->

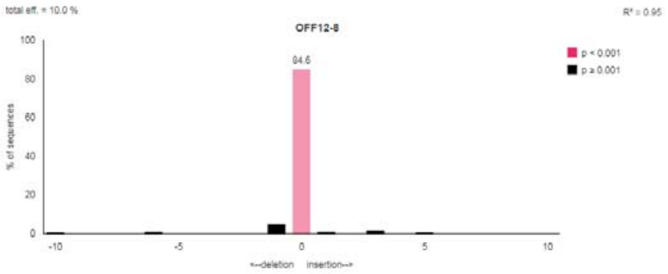
total eff. = 10.0 %
R² = 0.95
OFF12-8
84.6
p < 0.001
p ≥ 0.001
% of sequences
<--deletion insertion-->

gRNA1-OFF13

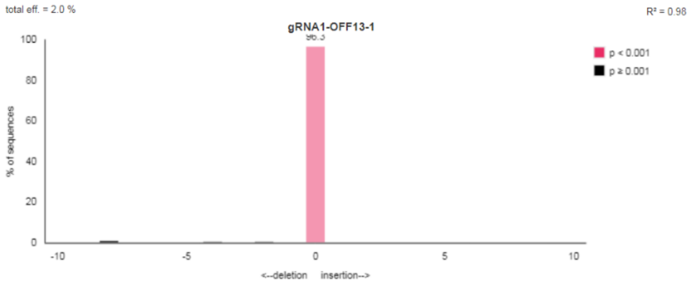
total eff. = 2.0 %
R² = 0.98
gRNA1-OFF13-1
p < 0.001
p ≥ 0.001
% of sequences
<--deletion insertion-->

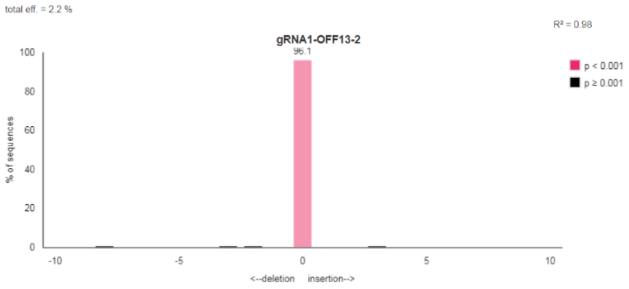
total eff. = 2.2 %
R² = 0.98
gRNA1-OFF13-2
96.1
p < 0.001
p ≥ 0.001
% of sequences
<--deletion insertion-->

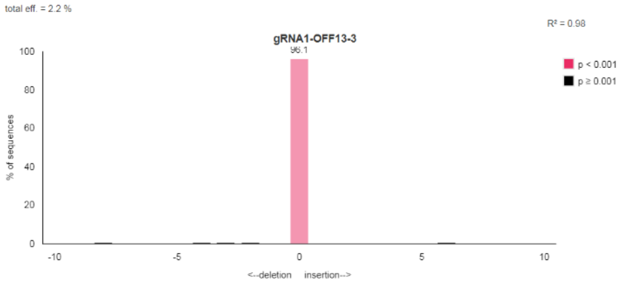
total eff. = 2.2 %
R² = 0.98
gRNA1-OFF13-3
96.1
p < 0.001
p ≥ 0.001
% of sequences
<--deletion insertion-->

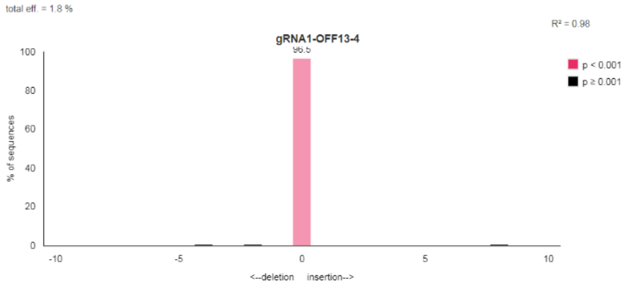
total eff. = 1.8 %
R² = 0.98
gRNA1-OFF13-4
p < 0.001
p ≥ 0.001
% of sequences
<--deletion insertion-->

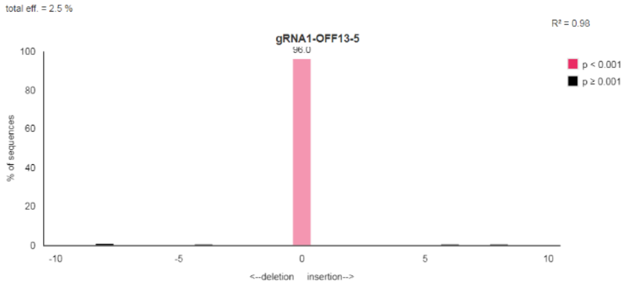
total eff. = 2.5 %
R² = 0.98
gRNA1-OFF13-5
96.0
p < 0.001
p ≥ 0.001
% of sequences
<--deletion insertion-->

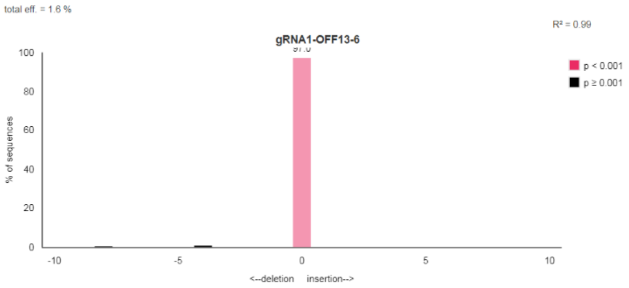
total eff. = 1.6 %
R² = 0.99
gRNA1-OFF13-6
p < 0.001
p ≥ 0.001
% of sequences
<--deletion insertion-->

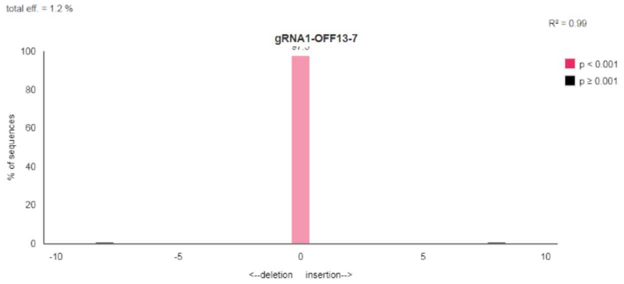
total eff. = 1.2 %
R² = 0.99
gRNA1-OFF13-7
p < 0.001
p ≥ 0.001
% of sequences
<--deletion insertion-->

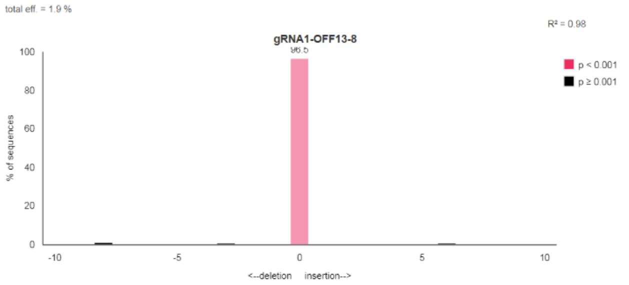
total eff. = 1.9 %
R² = 0.98
gRNA1-OFF13-8
p < 0.001
p ≥ 0.001
% of sequences
<--deletion insertion-->

gRNA1-OFF14

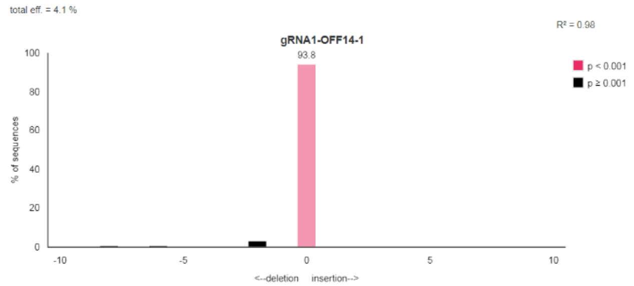
total eff. = 4.1 %
R² = 0.98
gRNA1-OFF14-1
93.8
p < 0.001
p ≥ 0.001
% of sequences
<--deletion insertion-->

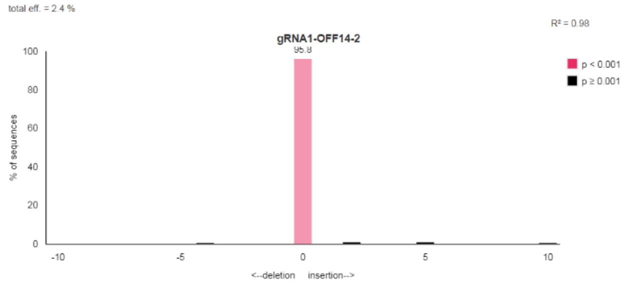
total eff. = 2.4 %
R² = 0.98
gRNA1-OFF14-2
p < 0.001
p ≥ 0.001
% of sequences
<--deletion insertion-->

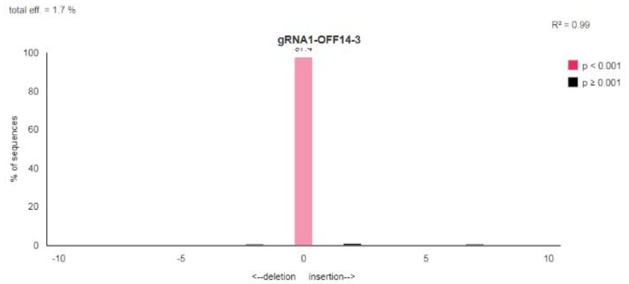
total eff. = 1.7 %
R² = 0.99
gRNA1-OFF14-3
p < 0.001
p ≥ 0.001
% of sequences
<--deletion insertion-->

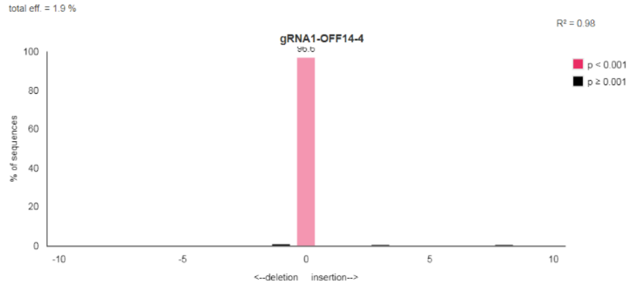
total eff. = 1.9 %
R² = 0.98
gRNA1-OFF14-4
p < 0.001
p ≥ 0.001
% of sequences
<--deletion insertion-->

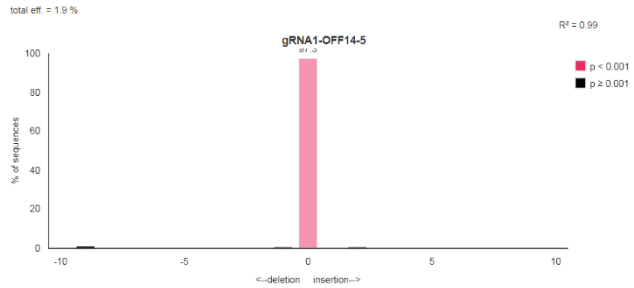
total eff. = 1.9 %
R² = 0.99
gRNA1-OFF14-5
p < 0.001
p ≥ 0.001
% of sequences
<--deletion insertion-->

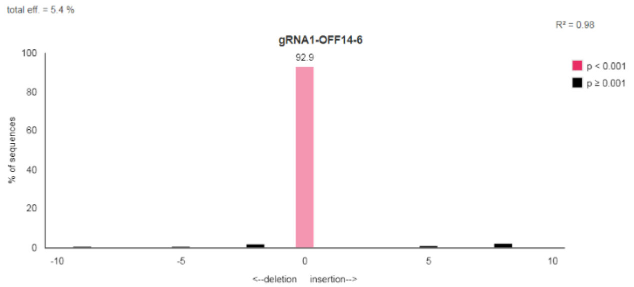
total eff. = 5.4 %
R² = 0.98
gRNA1-OFF14-6
92.9
p < 0.001
p ≥ 0.001
% of sequences
<--deletion insertion-->

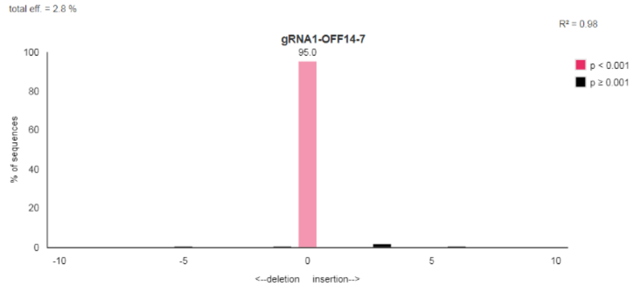
total eff. = 2.8 %
R² = 0.98
gRNA1-OFF14-7
95.0
p < 0.001
p ≥ 0.001
% of sequences
<--deletion insertion-->

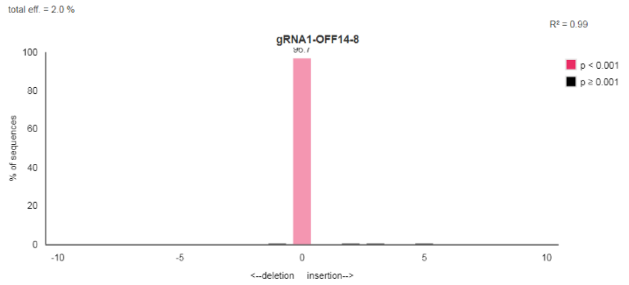
total eff. = 2.0 %
R² = 0.99
gRNA1-OFF14-8
p < 0.001
p ≥ 0.001
% of sequences
<--deletion insertion-->

gRNA1-OFF15

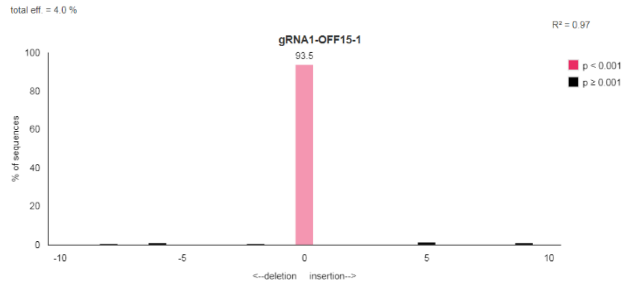
total eff. = 4.0 %
R² = 0.97
gRNA1-OFF15-1
93.5
p < 0.001
p ≥ 0.001
% of sequences
<--deletion insertion-->

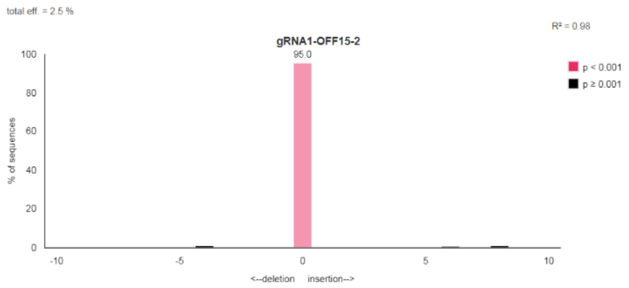
total eff. = 2.5 %
R² = 0.98
gRNA1-OFF15-2
95.0
p < 0.001
p ≥ 0.001
% of sequences
<--deletion insertion-->

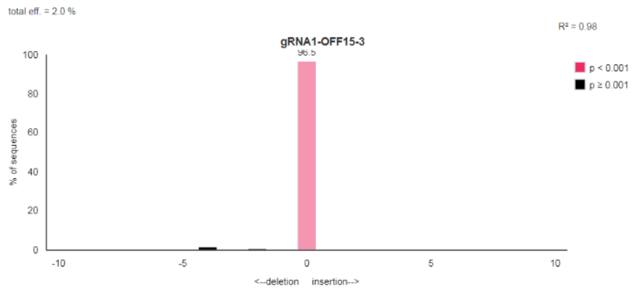
total eff. = 2.0 %
R² = 0.98
gRNA1-OFF15-3
p < 0.001
p ≥ 0.001
% of sequences
<--deletion insertion-->

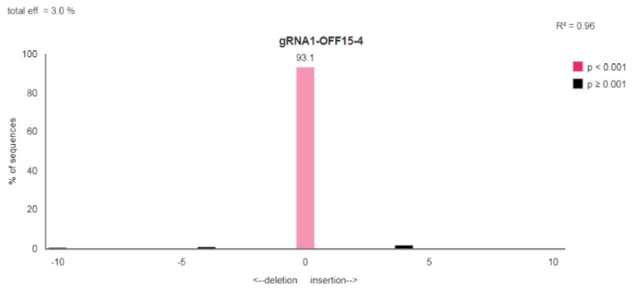
total eff. = 3.0 %
R² = 0.96
gRNA1-OFF15-4
93.1
p < 0.001
p ≥ 0.001
% of sequences
<--deletion insertion-->

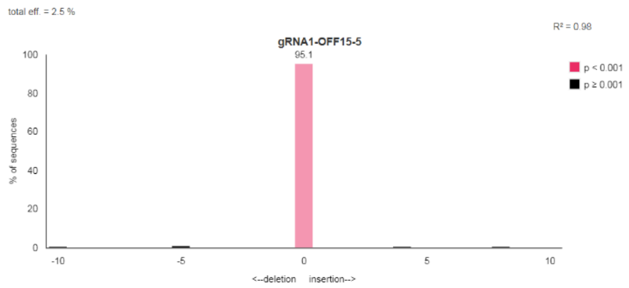
total eff. = 2.5 %
R² = 0.98
gRNA1-OFF15-5
95.1
p < 0.001
p ≥ 0.001
% of sequences
<--deletion insertion-->

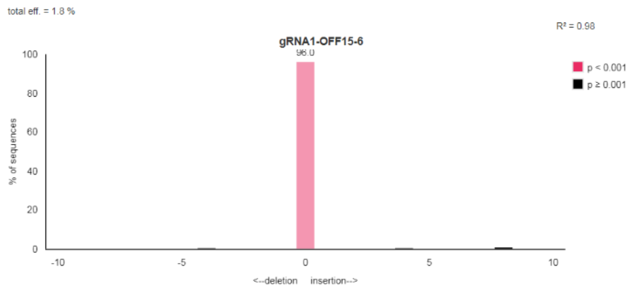
total eff. = 1.8 %
R² = 0.98
gRNA1-OFF15-6
p < 0.001
p ≥ 0.001
% of sequences
<--deletion insertion-->

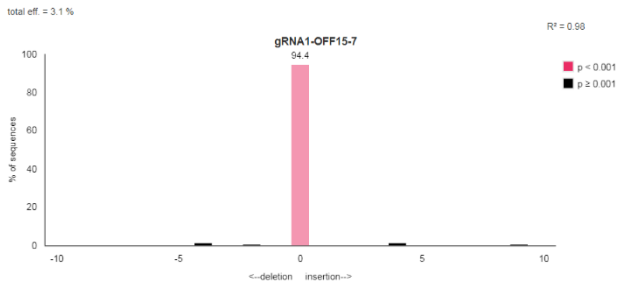
total eff. = 3.1 %
R² = 0.98
gRNA1-OFF15-7
94.4
p < 0.001
p ≥ 0.001
% of sequences
<--deletion insertion-->

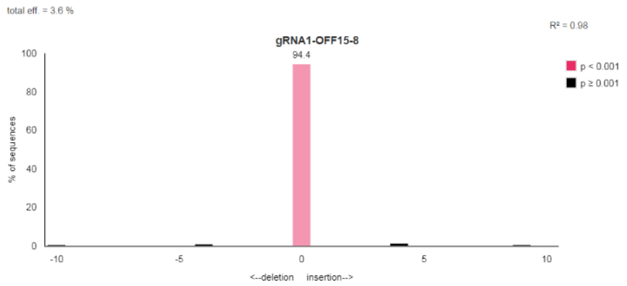
total eff. = 3.6 %
R² = 0.98
gRNA1-OFF15-8
94.4
p < 0.001
p ≥ 0.001
% of sequences
<--deletion insertion-->

gRNA1-OFF16

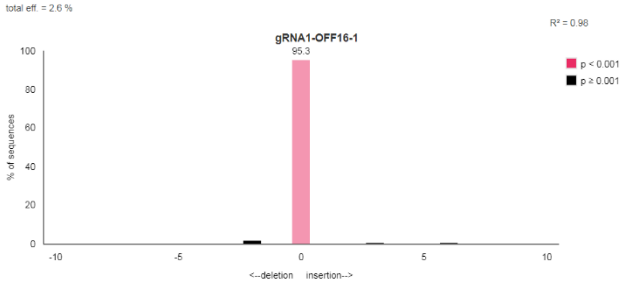
total eff. = 2.6 %
R² = 0.98
gRNA1-OFF16-1
95.3
p < 0.001
p ≥ 0.001
% of sequences
<--deletion insertion-->

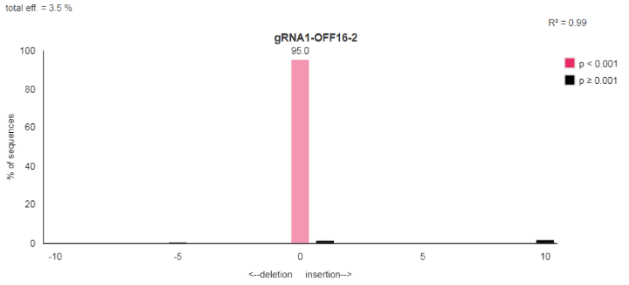
total eff. = 3.5 %
R² = 0.99
gRNA1-OFF16-2
95.0
p < 0.001
p ≥ 0.001
% of sequences
<--deletion insertion-->

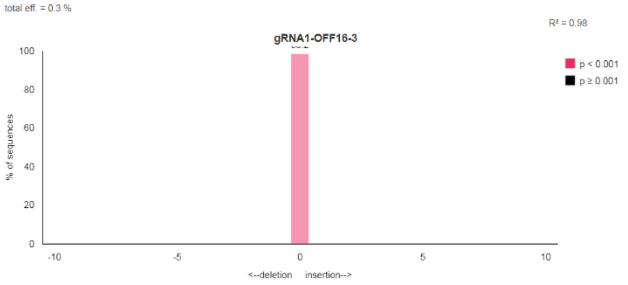
total eff. = 0.3 %
R² = 0.98
gRNA1-OFF16-3
p < 0.001
p ≥ 0.001
% of sequences
<--deletion insertion-->

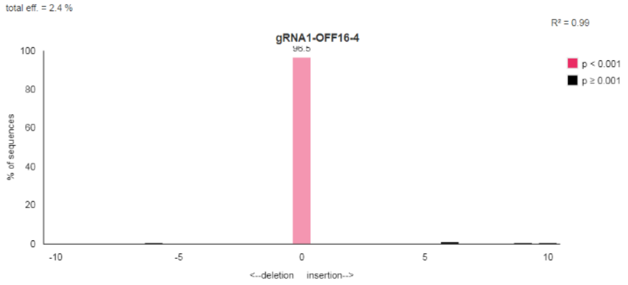
total eff. = 2.4 %
R² = 0.99
gRNA1-OFF16-4
p < 0.001
p ≥ 0.001
% of sequences
<--deletion insertion-->

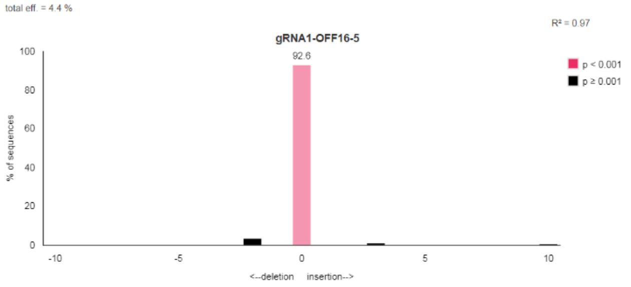
total eff. = 4.4 %
R² = 0.97
gRNA1-OFF16-5
92.6
p < 0.001
p ≥ 0.001
% of sequences
<--deletion insertion-->

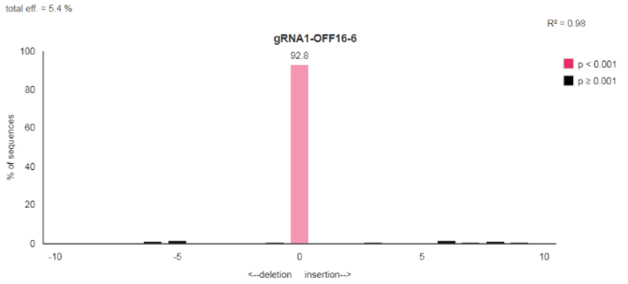
total eff. = 5.4 %
R² = 0.98
gRNA1-OFF16-6
92.8
p < 0.001
p ≥ 0.001
% of sequences
<--deletion insertion-->

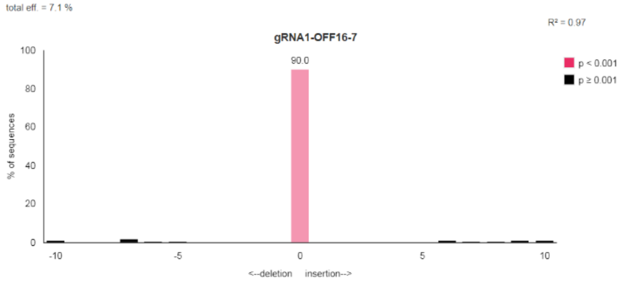
total eff. = 7.1 %
R² = 0.97
gRNA1-OFF16-7
90.0
p < 0.001
p ≥ 0.001
% of sequences
<--deletion insertion-->

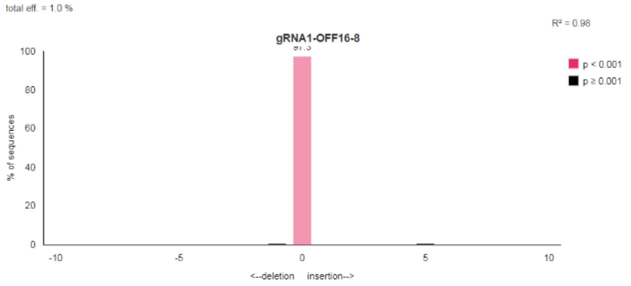
total eff. = 1.0 %
R² = 0.98
gRNA1-OFF16-8
p < 0.001
p ≥ 0.001
% of sequences
<--deletion insertion-->

gRNA1-OFF17

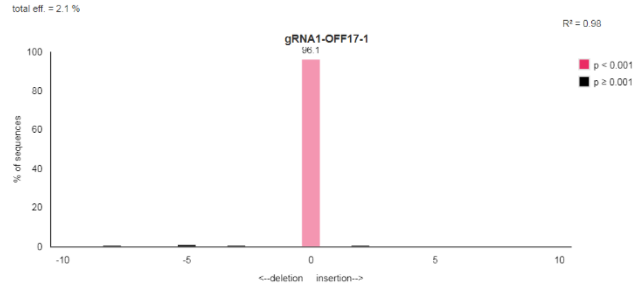
total eff. = 2.1 %
R² = 0.98
gRNA1-OFF17-1
p < 0.001
p ≥ 0.001
% of sequences
<--deletion insertion-->

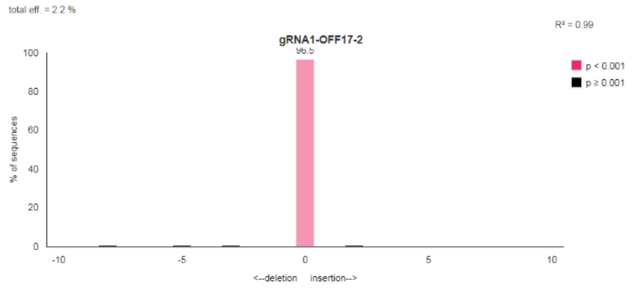
total eff. = 2.2 %
R² = 0.99
gRNA1-OFF17-2
p < 0.001
p ≥ 0.001
% of sequences
<--deletion insertion-->

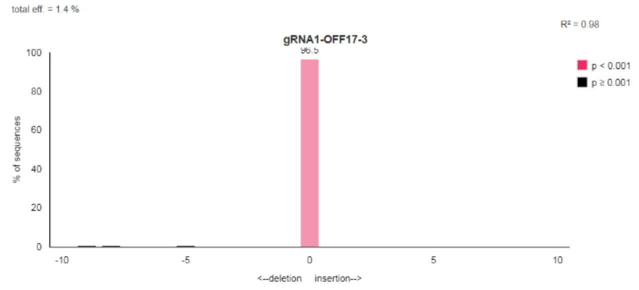
total eff. = 1.4 %
R² = 0.98
gRNA1-OFF17-3
p < 0.001
p ≥ 0.001
% of sequences
<--deletion insertion-->

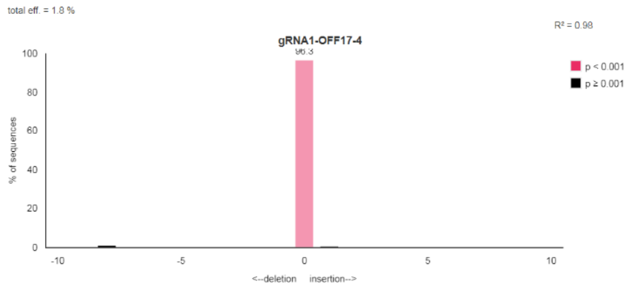
total eff. = 1.8 %
R² = 0.98
gRNA1-OFF17-4
p < 0.001
p ≥ 0.001
% of sequences
<--deletion insertion-->

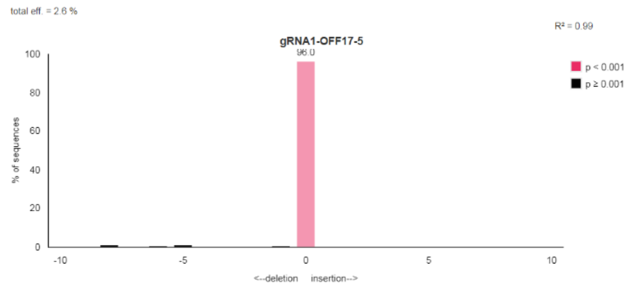
total eff. = 2.6 %
R² = 0.99
gRNA1-OFF17-5
96.0
p < 0.001
p ≥ 0.001
% of sequences
<--deletion insertion-->

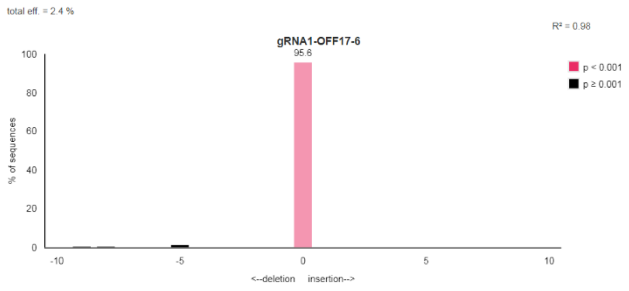
total eff. = 2.4 %
R² = 0.98
gRNA1-OFF17-6
95.6
p < 0.001
p ≥ 0.001
% of sequences
<--deletion insertion-->

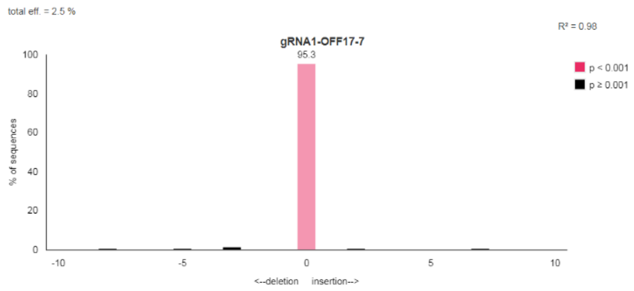
total eff. = 2.5 %
R² = 0.98
gRNA1-OFF17-7
95.3
p < 0.001
p ≥ 0.001
% of sequences
<--deletion insertion-->

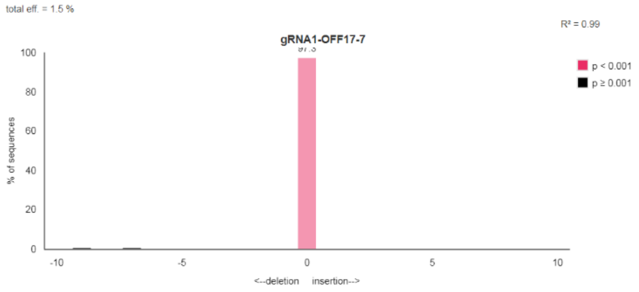
total eff. = 1.5 %
R² = 0.99
gRNA1-OFF17-7
p < 0.001
p ≥ 0.001
% of sequences
<--deletion insertion-->

A845B (gRNA2) T1 sample information

A total of 24 T1 plants derived from 10 different T0 lines were screened for off-target mutagenesis analysis. Seventeen plants were T-DNA positive (+), which constitutively expresses Cas9 nuclease, whereas seven were negative segregants (-) without Cas9 gene. PCR was carried for each gDNA sample, and then four reactions were pooled (Sample pool) for Sanger sequencing. Thus, six sanger sequencing reactions were carried out per off-target. Eighteen CIRCLE-seq found (gRNA2-OFF1-16, 19, 20) and two Cas-OFFinder predicted off-targets (gRNA2-OFF17,18) have been screened, but no evident off-target mutations were detected in the 24 T1 lines. HM, homozygous; BI, biallelic; HT, heterozygous; MO, mosaic; WT, wild type; MT, *gl2* mutant.

T0 genotype		A845B	T-DNA	T1 phenotype	Sample pool	Off-targets with mutation
(-6, -4+1)	BI	2-4-5	-	WT		
(-6, -4+1)	BI	2-4-12	-	WT	**Pool 1**	**None**
(-2, -5)	BI	3-3-6	+	MT		
(-2, -5)	BI	3-3-7	+	MT		
(-2, -5)	BI	3-3-10	+	MT		
(-2, -5)	BI	3-4-14	+	MT	**Pool 2**	**None**
(-2, -4)	BI	4-1-1	-	MT		
(-2, -4)	BI	4-1-12	-	MT		
(-2, -4)	BI	4-3-12	+	MT		
(-2, -4)	BI	4-3-13	+	MT	**Pool 3**	**None**
(-2, -3)	BI	5-3-2	+	WT		
(-2, -3)	BI	5-3-5	+	WT		
(-2, -3)	BI	5-3-11	+	WT		
(-2, -3)	BI	5-3-13	+	WT	**Pool 4**	**None**
(-6, -6)	HM	6-4-1	+	MT		
(-6, -6)	HM	6-4-2	+	MT		
(-6, -6)	HM	6-4-9	+	MT		
(-6, -6)	HM	6-4-13	-	WT	**Pool 5**	**None**
(-1, -6)	BI	7-3-7	+	MT		
(-1, -6)	BI	7-3-10	+	MT		
(-1, -6)	BI	7-3-15	+	MT		
(0, 0)	WT	8-3-5	-	WT	**Pool 6**	**None**
(0, -6)	HT	10-2-2	+	WT		
(0, -6)	HT	10-2-10	-	WT		

gRNA2-OFF1

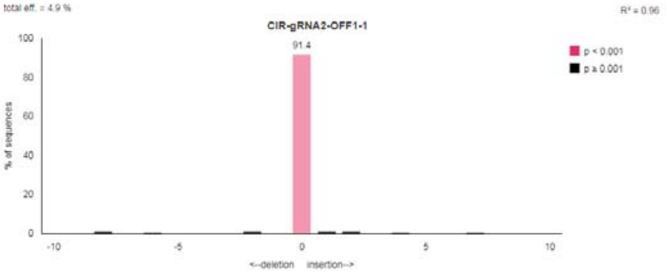
total eff. = 4.9 %
R² = 0.96
CIR-gRNA2-OFF1-1
91.4
p < 0.001
p ≥ 0.001
% of sequences
<--deletion insertion-->

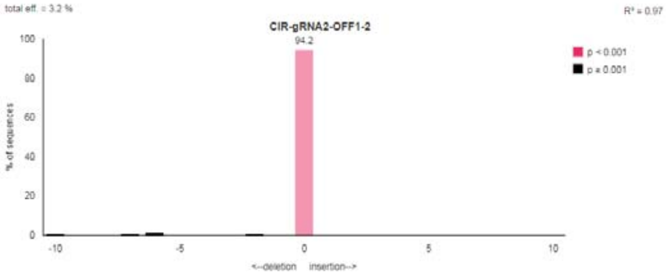
total eff. = 3.2 %
R² = 0.97
CIR-gRNA2-OFF1-2
94.2
p < 0.001
p ≥ 0.001
% of sequences
<--deletion insertion-->

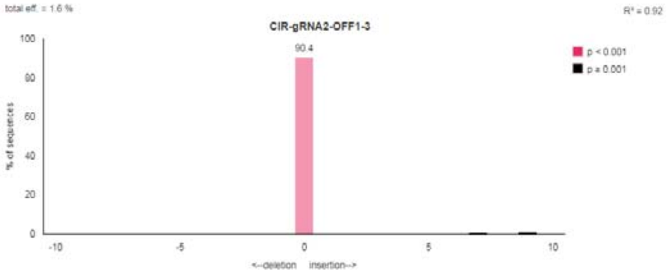
total eff. = 1.6 %
R² = 0.92
CIR-gRNA2-OFF1-3
90.4
p < 0.001
p ≥ 0.001
% of sequences
<--deletion insertion-->

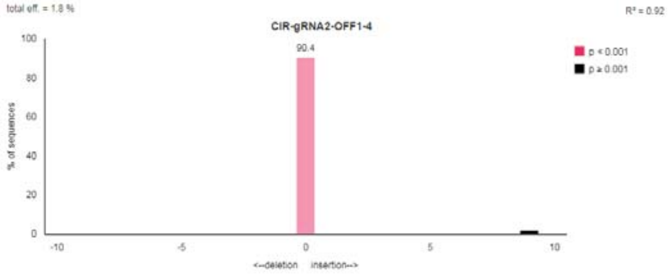
total eff. = 1.8 %
R² = 0.92
CIR-gRNA2-OFF1-4
90.4
p < 0.001
p ≥ 0.001
% of sequences
<--deletion insertion-->

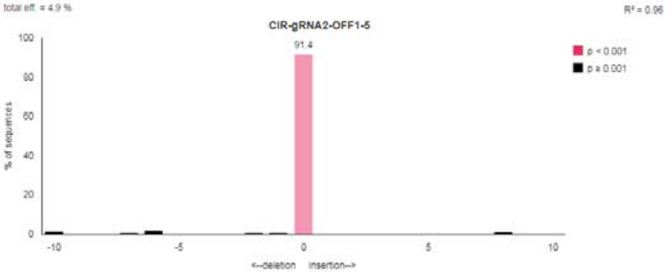
total eff. = 4.9 %
R² = 0.96
CIR-gRNA2-OFF1-5
91.4
p < 0.001
p ≥ 0.001
% of sequences
<--deletion insertion-->

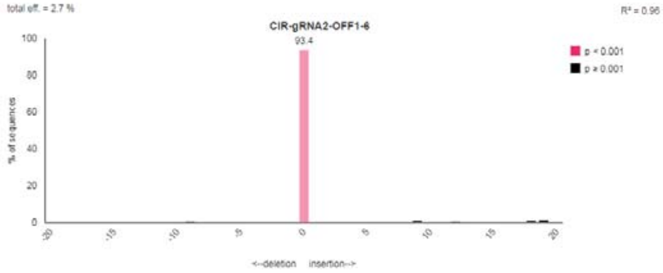
total eff. = 2.7 %
R² = 0.96
CIR-gRNA2-OFF1-6
93.4
p < 0.001
p ≥ 0.001
% of sequences
<--deletion insertion-->

gRNA2-OFF2

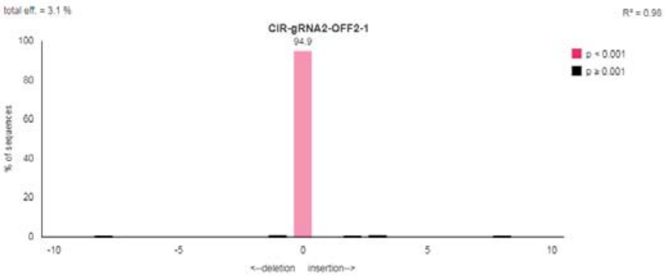

total eff. = 3.1 %
R² = 0.96
CIR-gRNA2-OFF2-1
94.9
p < 0.001
p ≥ 0.001
% of sequences
<--deletion insertion-->

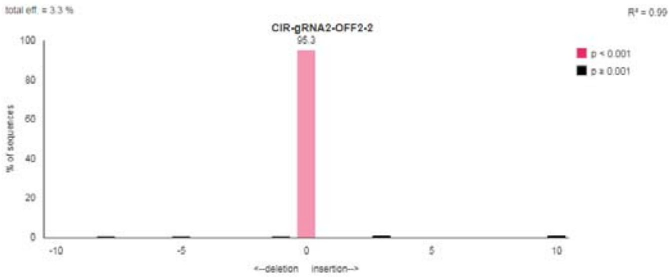

total eff. = 3.3 %
R² = 0.99
CIR-gRNA2-OFF2-2
95.3
p < 0.001
p ≥ 0.001
% of sequences
<--deletion insertion-->

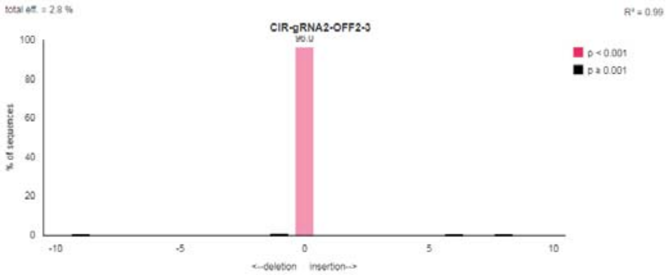

total eff. = 2.9 %
R² = 0.99
CIR-gRNA2-OFF2-3
p < 0.001
p ≥ 0.001
% of sequences
<--deletion insertion-->

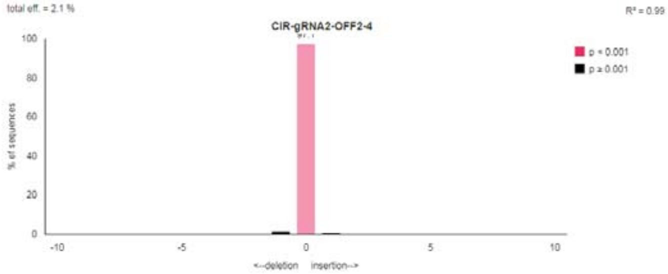

total eff. = 2.1 %
R² = 0.99
CIR-gRNA2-OFF2-4
p < 0.001
p ≥ 0.001
% of sequences
<--deletion insertion-->

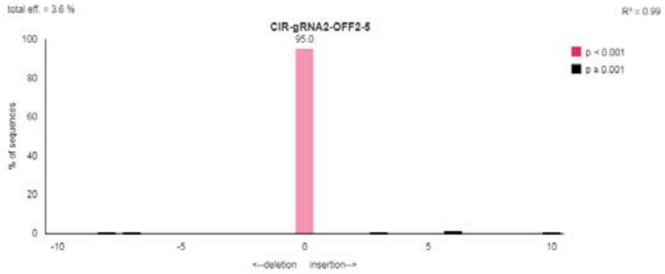

total eff. = 3.6 %
R² = 0.99
CIR-gRNA2-OFF2-5
95.0
p < 0.001
p ≥ 0.001
% of sequences
<--deletion insertion-->

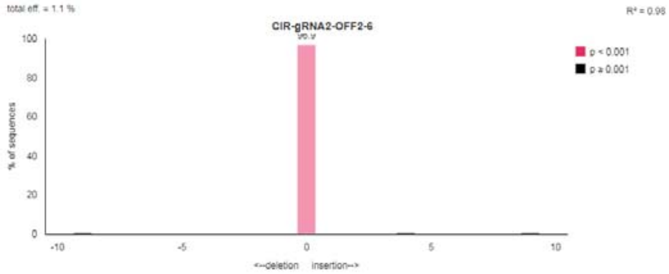

total eff. = 1.1 %
R² = 0.98
CIR-gRNA2-OFF2-6
p < 0.001
p ≥ 0.001
% of sequences
<--deletion insertion-->

gRNA2-OFF3

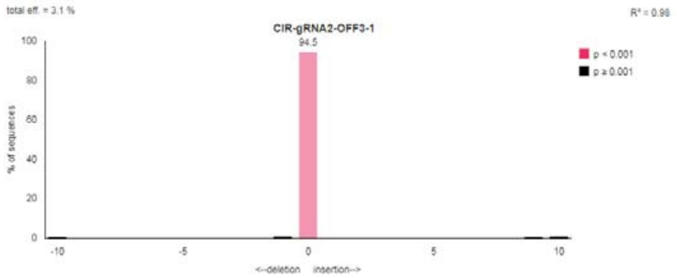

total eff. = 3.1 %
R² = 0.98
CIR-gRNA2-OFF3-1
94.5
p < 0.001
p ≥ 0.001
% of sequences
<--deletion insertion-->

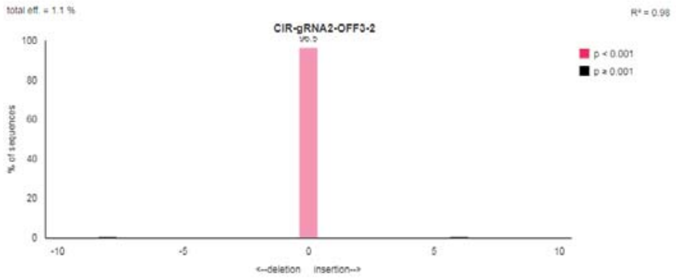

total eff. = 1.1 %
R² = 0.98
CIR-gRNA2-OFF3-2
p < 0.001
p ≥ 0.001
% of sequences
<--deletion insertion-->

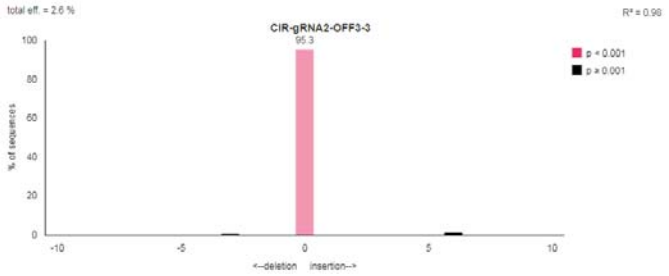

total eff. = 2.6 %
R² = 0.98
CIR-gRNA2-OFF3-3
95.3
p < 0.001
p ≥ 0.001
% of sequences
<--deletion insertion-->

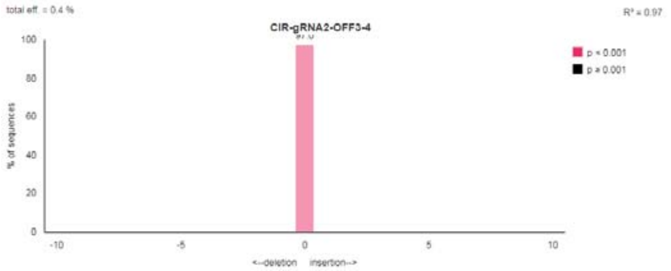

total eff. = 0.4 %
R² = 0.97
CIR-gRNA2-OFF3-4
p < 0.001
p ≥ 0.001
% of sequences
<--deletion insertion-->

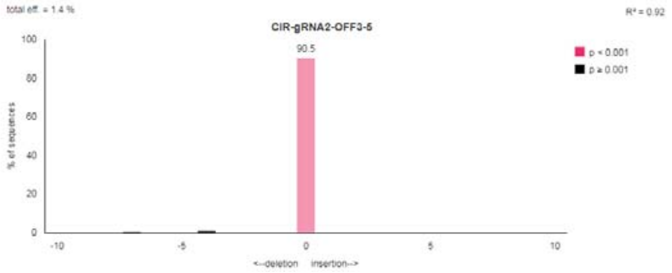

total eff. = 1.4 %
R² = 0.92
CIR-gRNA2-OFF3-5
90.5
p < 0.001
p ≥ 0.001
% of sequences
<--deletion insertion-->

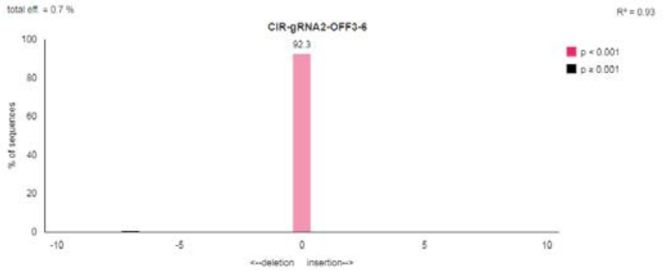

total eff. = 0.7 %
R² = 0.93
CIR-gRNA2-OFF3-6
92.3
p < 0.001
p ≥ 0.001
% of sequences
<--deletion insertion-->

gRNA2-OFF4

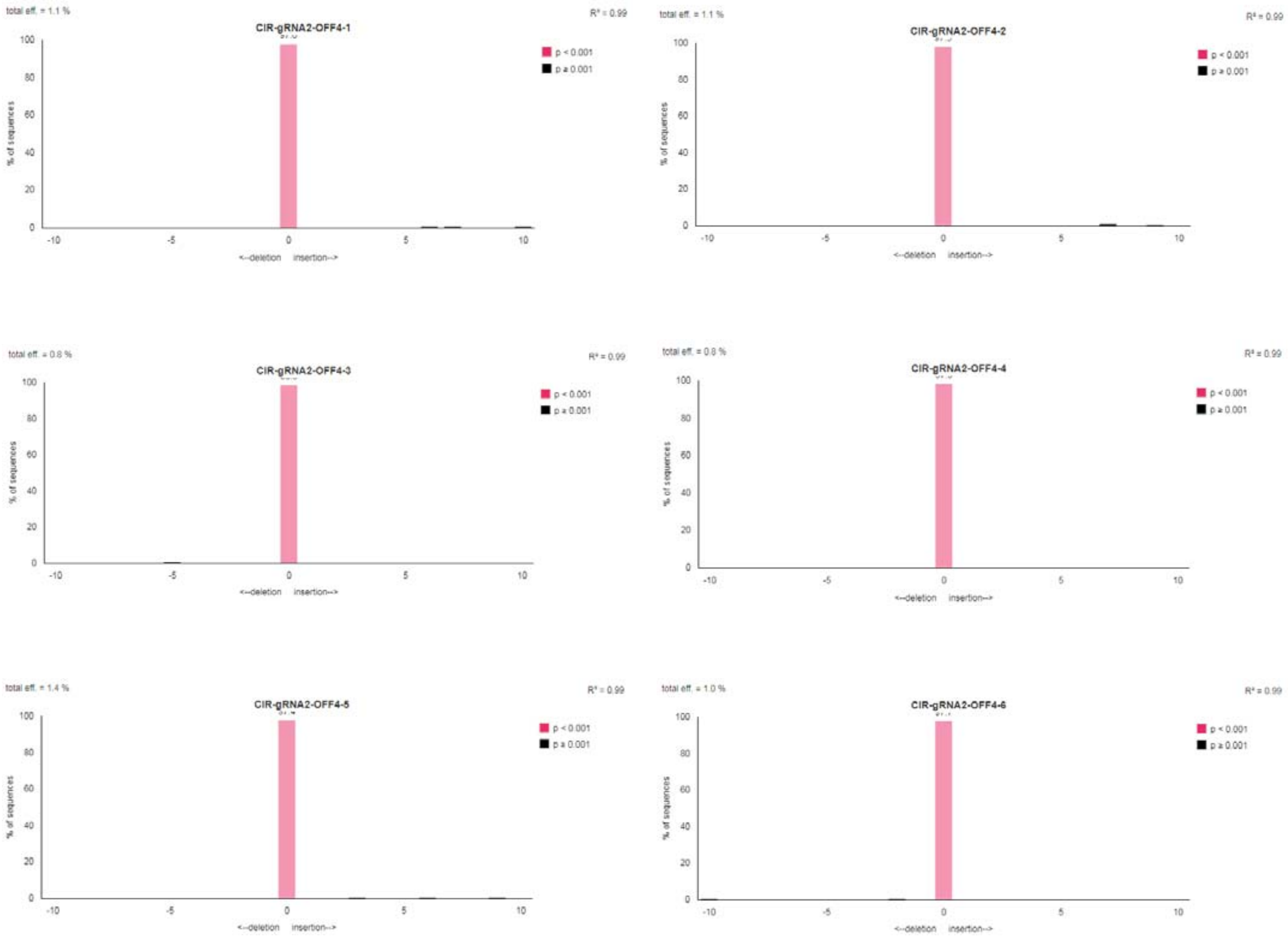

total eff. = 1.1 %
R² = 0.99
CIR-gRNA2-OFF4-1
% of sequences
p < 0.001
p ≥ 0.001
<--deletion insertion-->
total eff. = 1.1 %
R² = 0.99
CIR-gRNA2-OFF4-2
total eff. = 0.8 %
R² = 0.99
CIR-gRNA2-OFF4-3
total eff. = 0.8 %
R² = 0.99
CIR-gRNA2-OFF4-4
total eff. = 1.4 %
R² = 0.99
CIR-gRNA2-OFF4-5
total eff. = 1.0 %
R² = 0.99
CIR-gRNA2-OFF4-6

gRNA2-OFF5

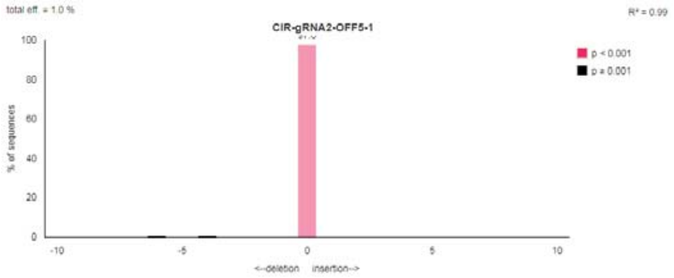
total eff. = 1.0 %
R² = 0.99
CIR-gRNA2-OFF5-1
p < 0.001
p ≥ 0.001
% of sequences
<--deletion insertion-->

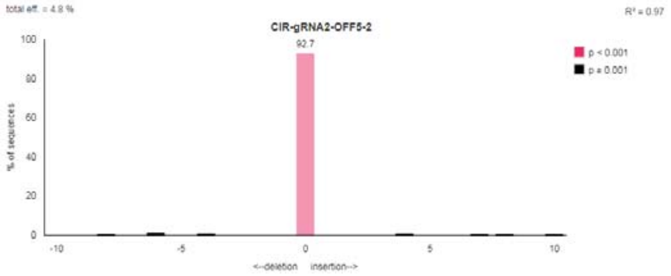
total eff. = 4.8 %
R² = 0.97
CIR-gRNA2-OFF5-2
92.7
p < 0.001
p ≥ 0.001
% of sequences
<--deletion insertion-->

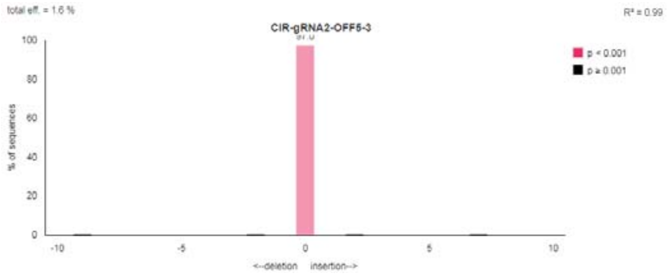
total eff. = 1.6 %
R² = 0.99
CIR-gRNA2-OFF5-3
p < 0.001
p ≥ 0.001
% of sequences
<--deletion insertion-->

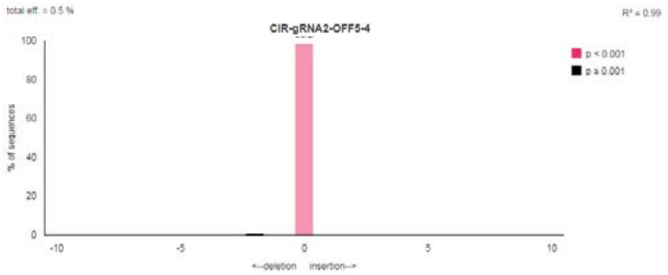
total eff. = 0.5 %
R² = 0.99
CIR-gRNA2-OFF5-4
p < 0.001
p ≥ 0.001
% of sequences
<--deletion insertion-->

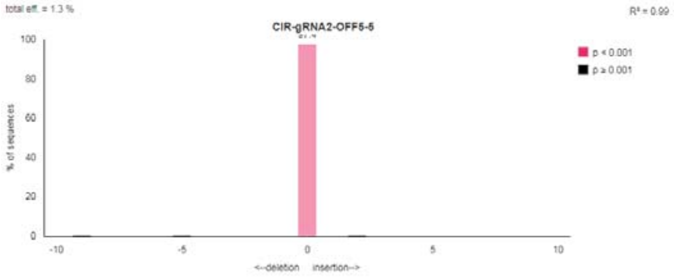
total eff. = 1.3 %
R² = 0.99
CIR-gRNA2-OFF5-5
p < 0.001
p ≥ 0.001
% of sequences
<--deletion insertion-->

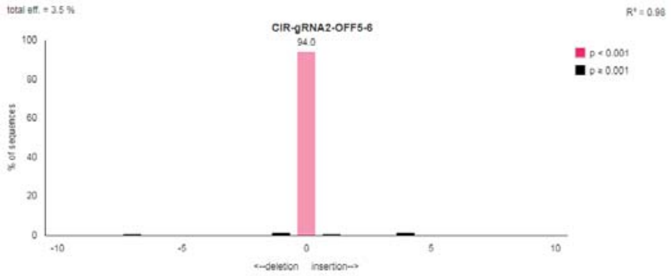
total eff. = 3.5 %
R² = 0.98
CIR-gRNA2-OFF5-6
94.0
p < 0.001
p ≥ 0.001
% of sequences
<--deletion insertion-->

gRNA2-OFF6

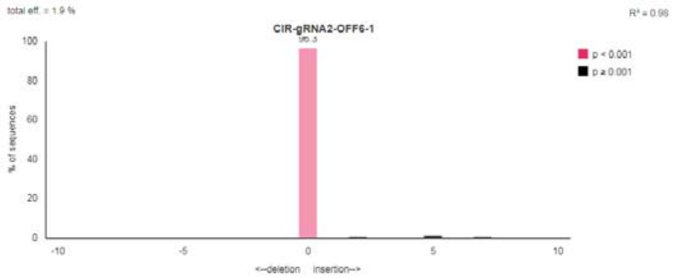

total eff. = 1.9 %
R² = 0.98
CIR-gRNA2-OFF6-1
p < 0.001
p ≥ 0.001
% of sequences
<--deletion insertion-->

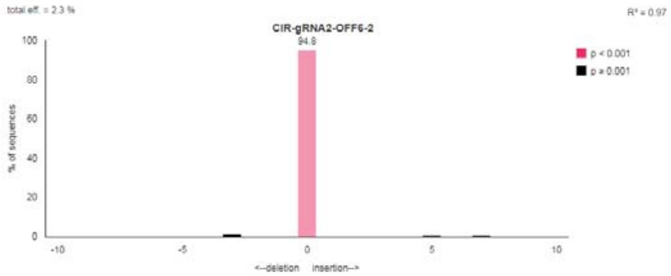

total eff. = 2.3 %
R² = 0.97
CIR-gRNA2-OFF6-2
94.8
p < 0.001
p ≥ 0.001
% of sequences
<--deletion insertion-->

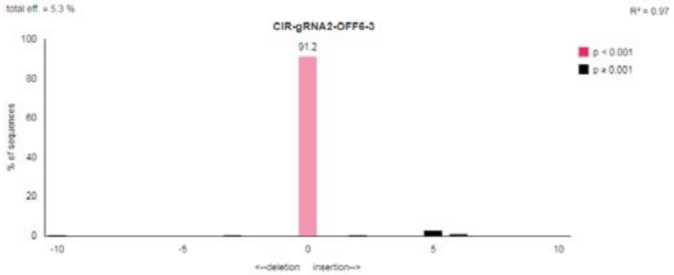

total eff. = 5.3 %
R² = 0.97
CIR-gRNA2-OFF6-3
91.2
p < 0.001
p ≥ 0.001
% of sequences
<--deletion insertion-->

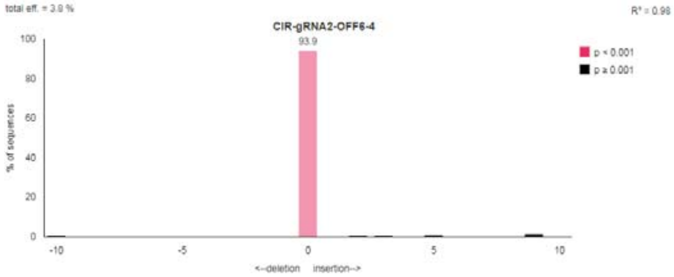

total eff. = 3.8 %
R² = 0.98
CIR-gRNA2-OFF6-4
93.9
p < 0.001
p ≥ 0.001
% of sequences
<--deletion insertion-->

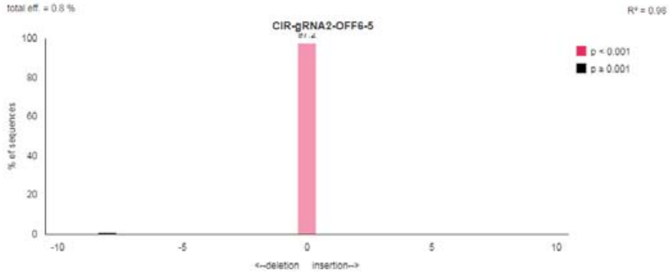

total eff. = 0.8 %
R² = 0.98
CIR-gRNA2-OFF6-5
p < 0.001
p ≥ 0.001
% of sequences
<--deletion insertion-->

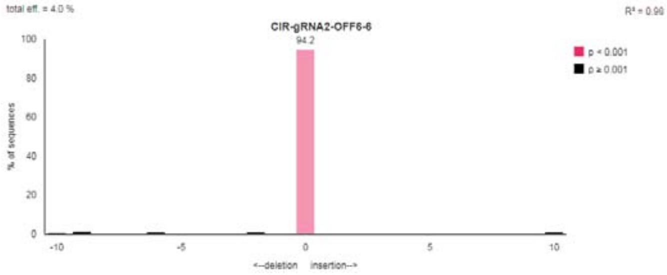

total eff. = 4.0 %
R² = 0.98
CIR-gRNA2-OFF6-6
94.2
p < 0.001
p ≥ 0.001
% of sequences
<--deletion insertion-->

gRNA2-OFF7

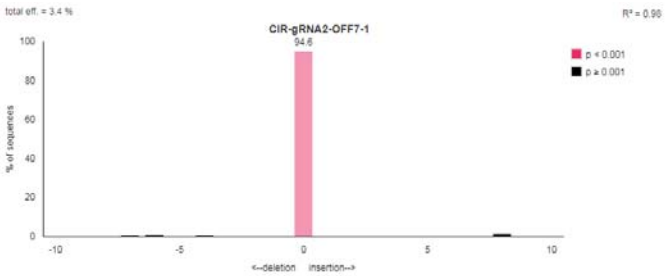
total eff. = 3.4 %
R² = 0.98
CIR-gRNA2-OFF7-1
94.6
p < 0.001
p ≥ 0.001
% of sequences
<--deletion insertion-->

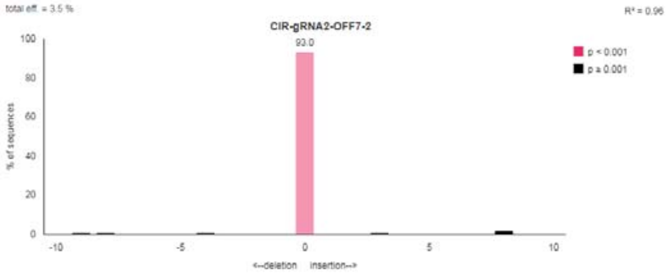
total eff. = 3.5 %
R² = 0.96
CIR-gRNA2-OFF7-2
93.0
p < 0.001
p ≥ 0.001
% of sequences
<--deletion insertion-->

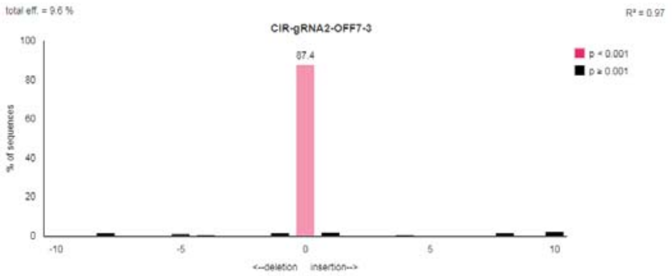
total eff. = 9.6 %
R² = 0.97
CIR-gRNA2-OFF7-3
87.4
p < 0.001
p ≥ 0.001
% of sequences
<--deletion insertion-->

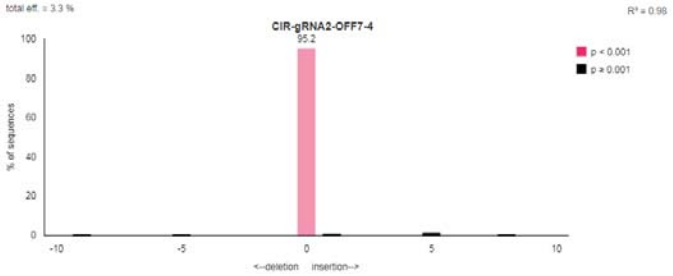
total eff. = 3.3 %
R² = 0.98
CIR-gRNA2-OFF7-4
95.2
p < 0.001
p ≥ 0.001
% of sequences
<--deletion insertion-->

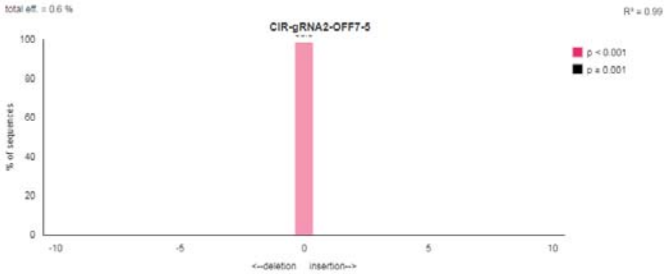
total eff. = 0.6 %
R² = 0.99
CIR-gRNA2-OFF7-5
p < 0.001
p ≥ 0.001
% of sequences
<--deletion insertion-->

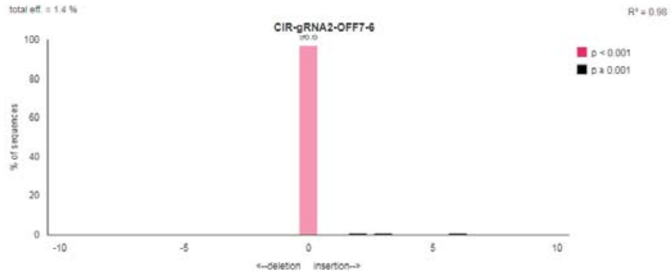
total eff. = 1.4 %
R² = 0.98
CIR-gRNA2-OFF7-6
p < 0.001
p ≥ 0.001
% of sequences
<--deletion insertion-->

gRNA2-OFF8

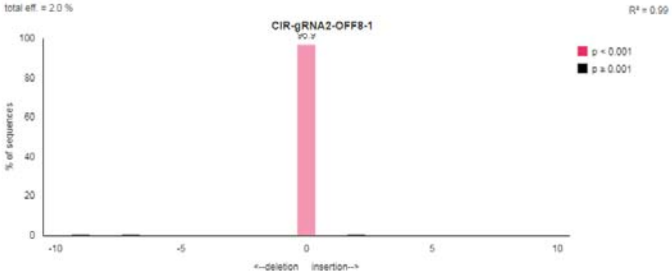
total eff. = 2.0 %
R² = 0.99
CIR-gRNA2-OFF8-1
p < 0.001
p ≥ 0.001
% of sequences
<--deletion insertion-->

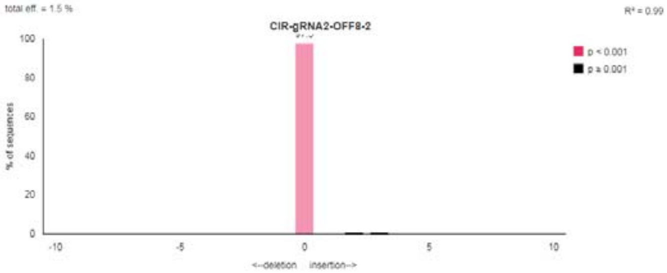
total eff. = 1.5 %
R² = 0.99
CIR-gRNA2-OFF8-2
p < 0.001
p ≥ 0.001
% of sequences
<--deletion insertion-->

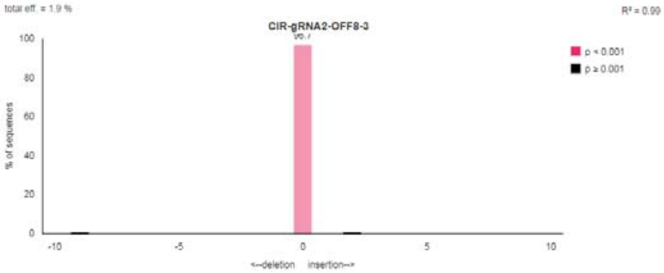
total eff. = 1.9 %
R² = 0.99
CIR-gRNA2-OFF8-3
p < 0.001
p ≥ 0.001
% of sequences
<--deletion insertion-->

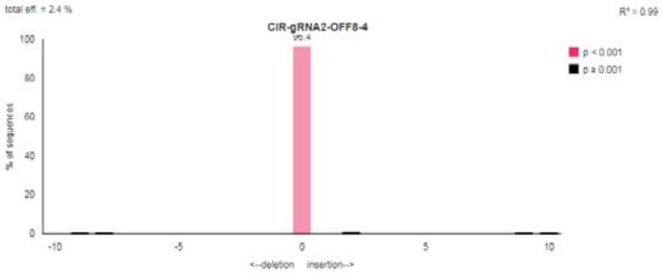
total eff. = 2.4 %
R² = 0.99
CIR-gRNA2-OFF8-4
p < 0.001
p ≥ 0.001
% of sequences
<--deletion insertion-->

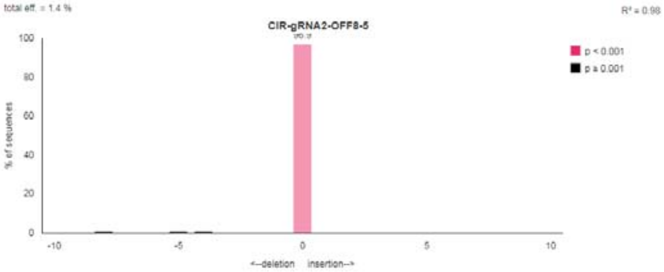
total eff. = 1.4 %
R² = 0.98
CIR-gRNA2-OFF8-5
p < 0.001
p ≥ 0.001
% of sequences
<--deletion insertion-->

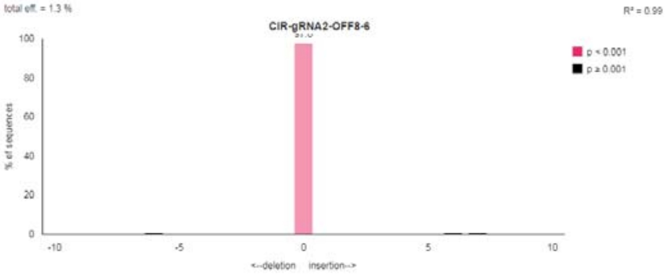
total eff. = 1.3 %
R² = 0.99
CIR-gRNA2-OFF8-6
p < 0.001
p ≥ 0.001
% of sequences
<--deletion insertion-->

gRNA2-OFF9

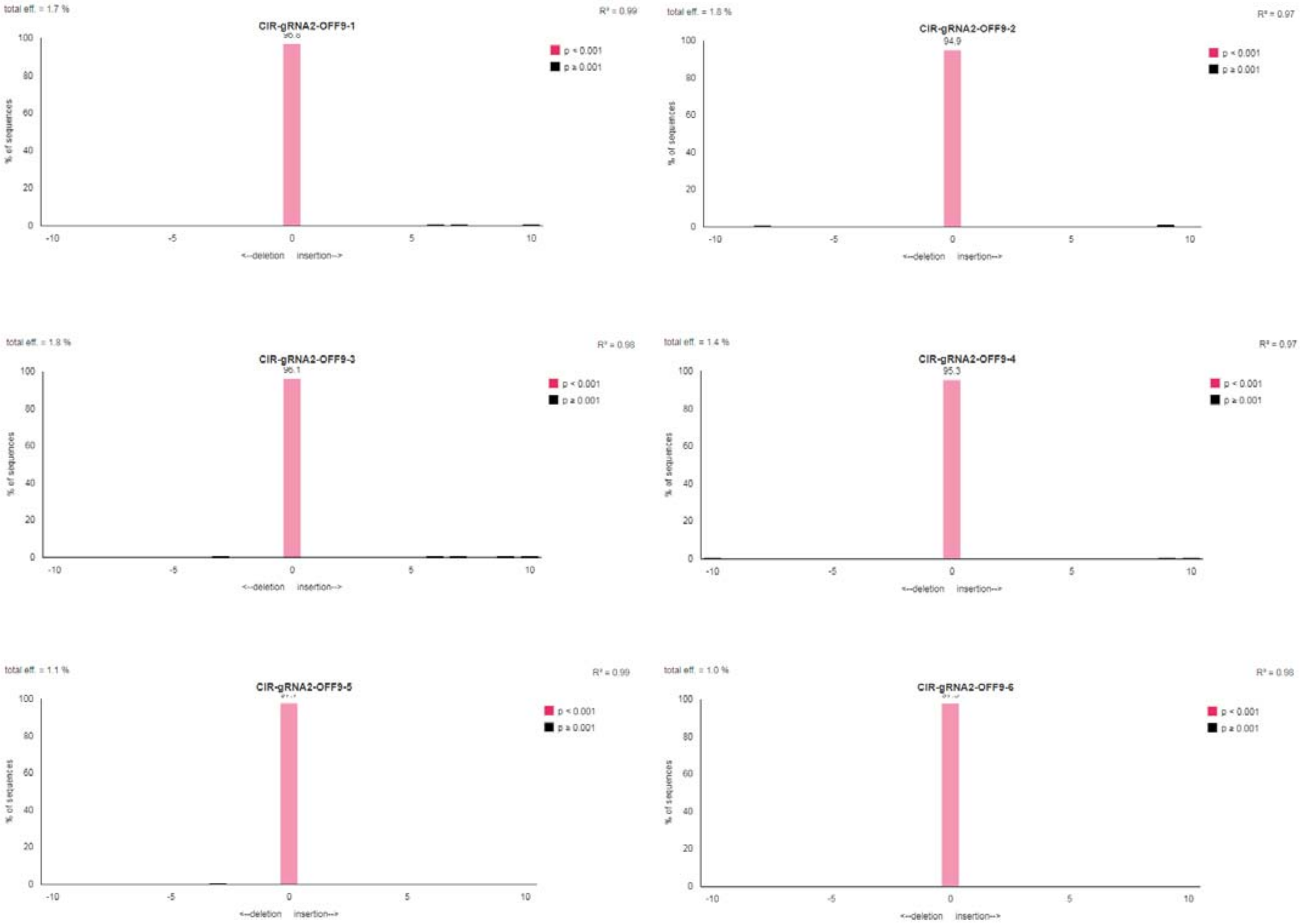

total eff. = 1.7 %
R² = 0.99
CIR-gRNA2-OFF9-1
p < 0.001
p ≥ 0.001
% of sequences
<--deletion insertion-->
total eff. = 1.8 %
R² = 0.97
CIR-gRNA2-OFF9-2
94.9
p < 0.001
p ≥ 0.001
% of sequences
<--deletion insertion-->
total eff. = 1.8 %
R² = 0.98
CIR-gRNA2-OFF9-3
96.1
p < 0.001
p ≥ 0.001
% of sequences
<--deletion insertion-->
total eff. = 1.4 %
R² = 0.97
CIR-gRNA2-OFF9-4
95.3
p < 0.001
p ≥ 0.001
% of sequences
<--deletion insertion-->
total eff. = 1.1 %
R² = 0.99
CIR-gRNA2-OFF9-5
p < 0.001
p ≥ 0.001
% of sequences
<--deletion insertion-->
total eff. = 1.0 %
R² = 0.98
CIR-gRNA2-OFF9-6
p < 0.001
p ≥ 0.001
% of sequences
<--deletion insertion-->

gRNA2-OFF10

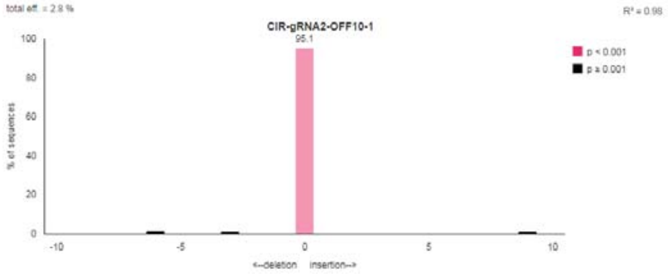
total eff. = 2.8 %
R² = 0.98
CIR-gRNA2-OFF10-1
95.1
p < 0.001
p ≥ 0.001
% of sequences
<--deletion insertion-->

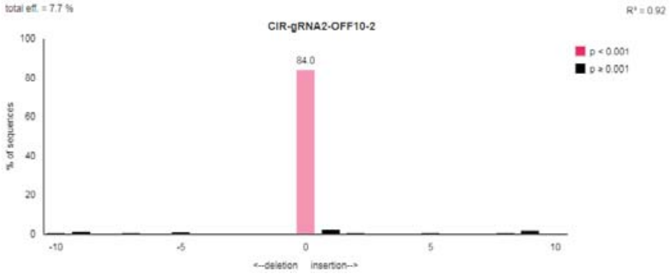
total eff. = 7.7 %
R² = 0.92
CIR-gRNA2-OFF10-2
84.0
p < 0.001
p ≥ 0.001
% of sequences
<--deletion insertion-->

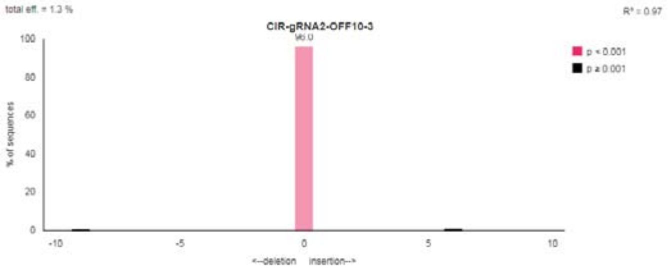
total eff. = 1.3 %
R² = 0.97
CIR-gRNA2-OFF10-3
96.0
p < 0.001
p ≥ 0.001
% of sequences
<--deletion insertion-->

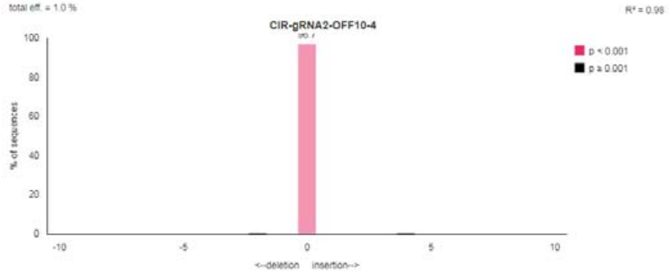
total eff. = 1.0 %
R² = 0.96
CIR-gRNA2-OFF10-4
p < 0.001
p ≥ 0.001
% of sequences
<--deletion insertion-->

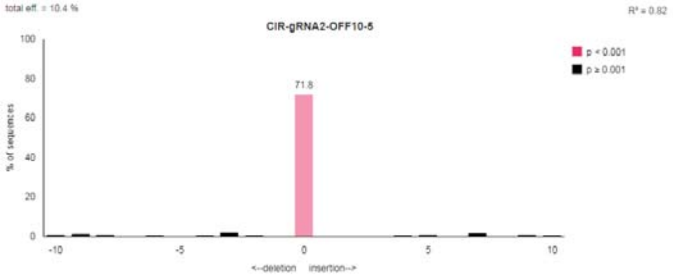
total eff. = 10.4 %
R² = 0.82
CIR-gRNA2-OFF10-5
71.8
p < 0.001
p ≥ 0.001
% of sequences
<--deletion insertion-->

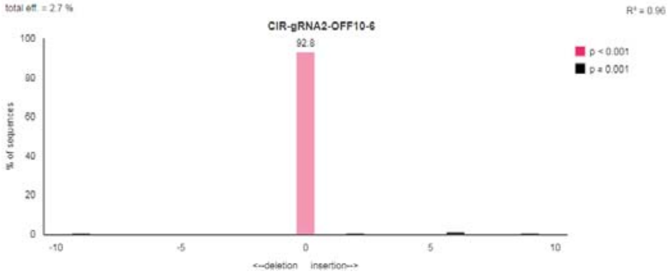
total eff. = 2.7 %
R² = 0.96
CIR-gRNA2-OFF10-6
92.8
p < 0.001
p ≥ 0.001
% of sequences
<--deletion insertion-->

gRNA2-OFF11

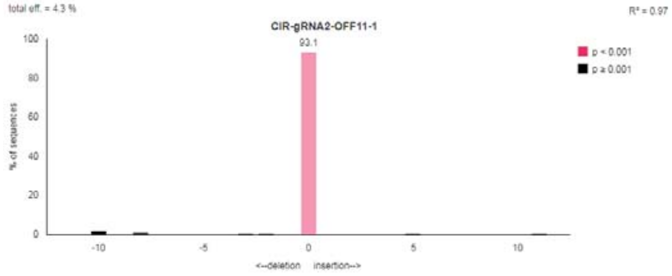
total eff. = 4.3 %
R² = 0.97
CIR-gRNA2-OFF11-1
93.1
p < 0.001
p ≥ 0.001
% of sequences
<--deletion insertion-->

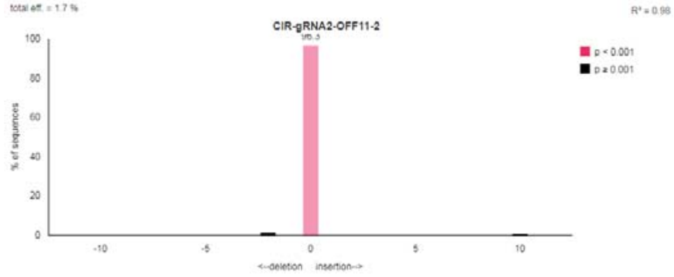
total eff. = 1.7 %
R² = 0.98
CIR-gRNA2-OFF11-2
p < 0.001
p ≥ 0.001
% of sequences
<--deletion insertion-->

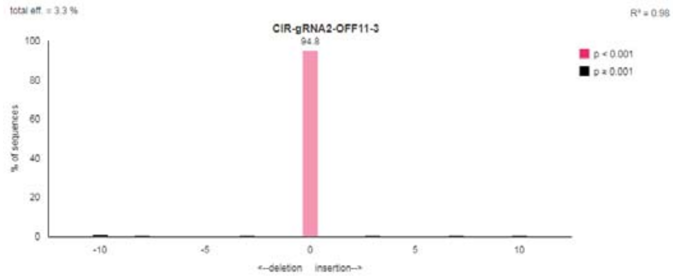
total eff. = 3.3 %
R² = 0.98
CIR-gRNA2-OFF11-3
94.8
p < 0.001
p ≥ 0.001
% of sequences
<--deletion insertion-->

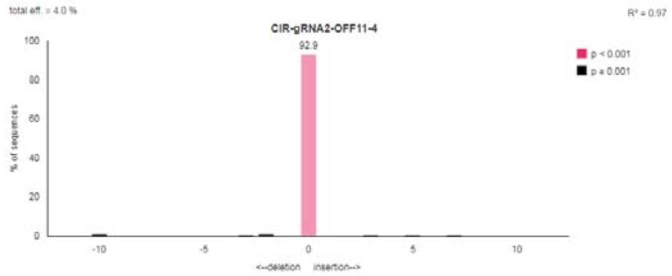
total eff. = 4.0 %
R² = 0.97
CIR-gRNA2-OFF11-4
92.9
p < 0.001
p ≥ 0.001
% of sequences
<--deletion insertion-->

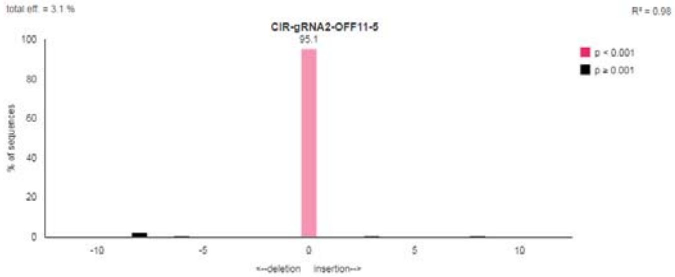
total eff. = 3.1 %
R² = 0.98
CIR-gRNA2-OFF11-5
95.1
p < 0.001
p ≥ 0.001
% of sequences
<--deletion insertion-->

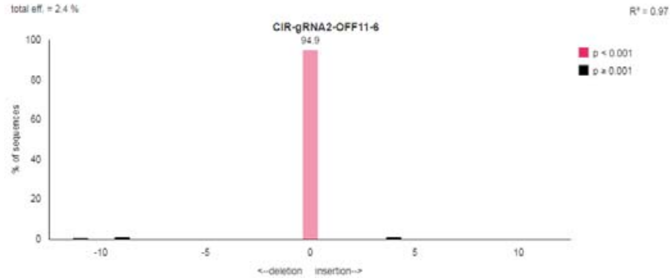
total eff. = 2.4 %
R² = 0.97
CIR-gRNA2-OFF11-6
94.9
p < 0.001
p ≥ 0.001
% of sequences
<--deletion insertion-->

gRNA2-OFF12

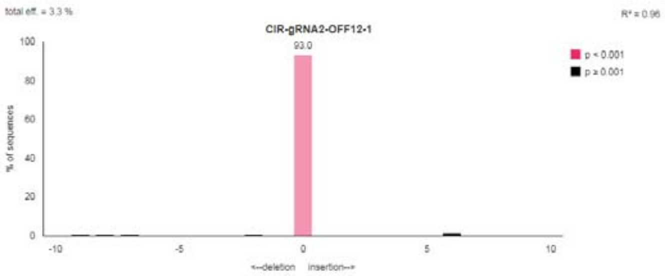

total eff. = 3.3 %
R² = 0.96
CIR-gRNA2-OFF12-1
93.0
p < 0.001
p ≥ 0.001
% of sequences
<--deletion insertion-->

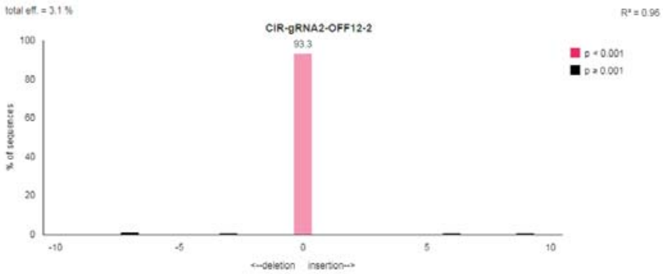

total eff. = 3.1 %
R² = 0.96
CIR-gRNA2-OFF12-2
93.3
p < 0.001
p ≥ 0.001
% of sequences
<--deletion insertion-->

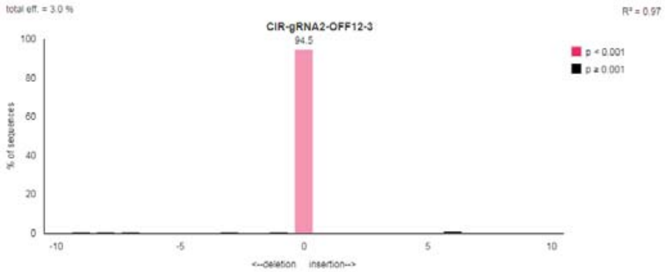

total eff. = 3.0 %
R² = 0.97
CIR-gRNA2-OFF12-3
94.5
p < 0.001
p ≥ 0.001
% of sequences
<--deletion insertion-->

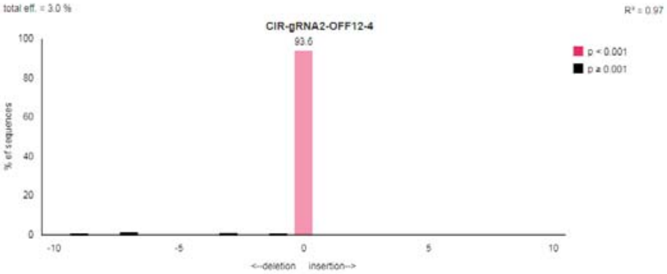

total eff. = 3.0 %
R² = 0.97
CIR-gRNA2-OFF12-4
93.6
p < 0.001
p ≥ 0.001
% of sequences
<--deletion insertion-->

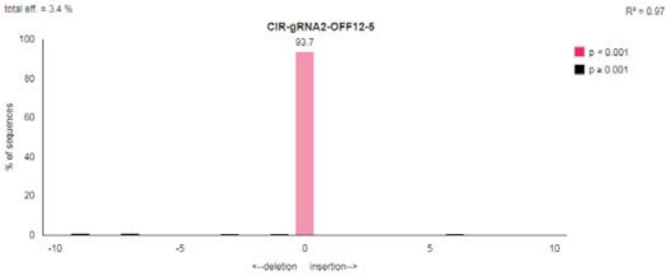

total eff. = 3.4 %
R² = 0.97
CIR-gRNA2-OFF12-5
93.7
p < 0.001
p ≥ 0.001
% of sequences
<--deletion insertion-->

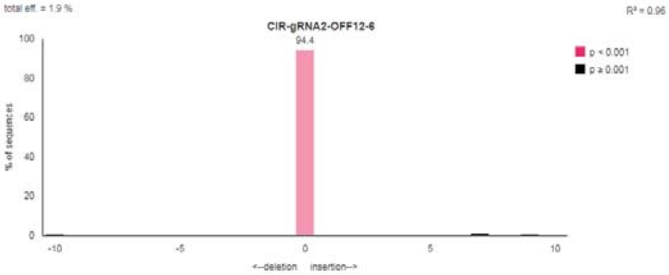

total eff. = 1.9 %
R² = 0.96
CIR-gRNA2-OFF12-6
94.4
p < 0.001
p ≥ 0.001
% of sequences
<--deletion insertion-->

gRNA2-OFF13

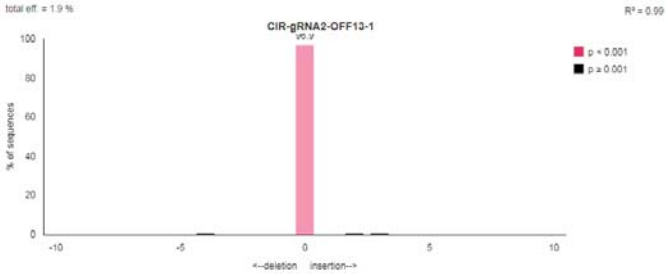
total eff. = 1.9 %
R² = 0.99
CIR-gRNA2-OFF13-1
p < 0.001
p ≥ 0.001
% of sequences
<--deletion insertion-->

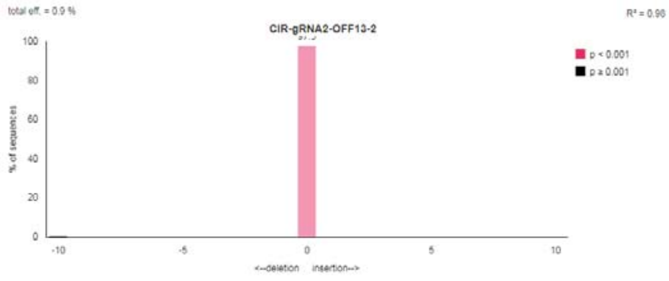
total eff. = 0.9 %
R² = 0.98
CIR-gRNA2-OFF13-2
p < 0.001
p ≥ 0.001
% of sequences
<--deletion insertion-->

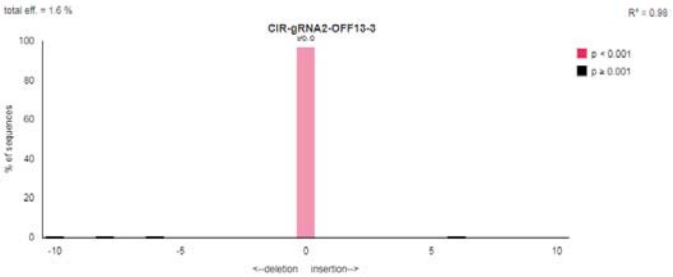
total eff. = 1.6 %
R² = 0.98
CIR-gRNA2-OFF13-3
p < 0.001
p ≥ 0.001
% of sequences
<--deletion insertion-->

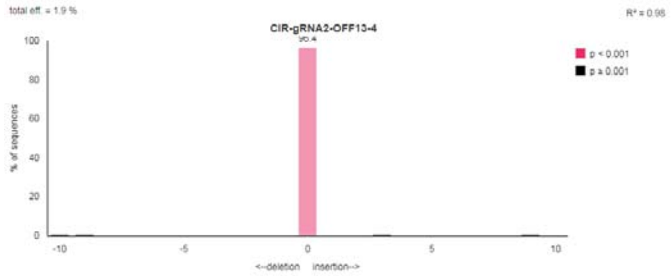
total eff. = 1.9 %
R² = 0.98
CIR-gRNA2-OFF13-4
p < 0.001
p ≥ 0.001
% of sequences
<--deletion insertion-->

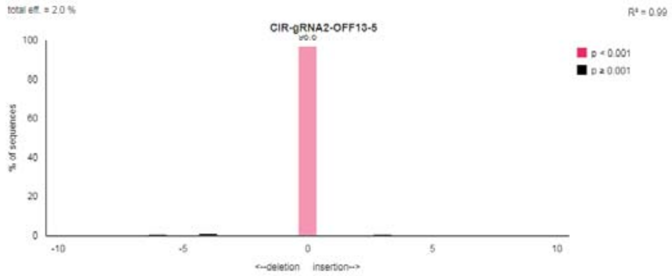
total eff. = 2.0 %
R² = 0.99
CIR-gRNA2-OFF13-5
p < 0.001
p ≥ 0.001
% of sequences
<--deletion insertion-->

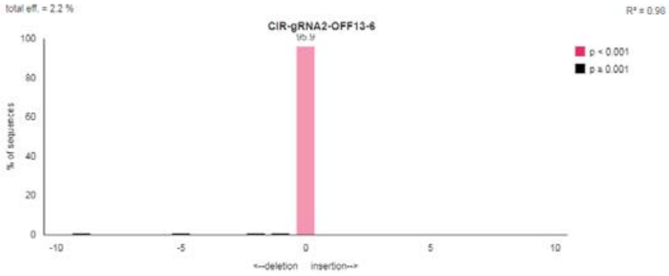
total eff. = 2.2 %
R² = 0.98
CIR-gRNA2-OFF13-6
p < 0.001
p ≥ 0.001
% of sequences
<--deletion insertion-->

gRNA2-OFF14

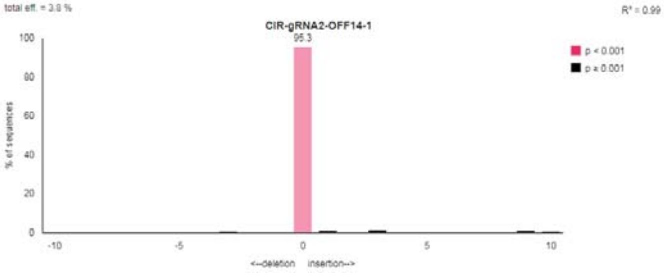
total eff. = 3.8 %
R^2 = 0.99
CIR-gRNA2-OFF14-1
95.3
p < 0.001
p ≥ 0.001
% of sequences
<--deletion insertion-->

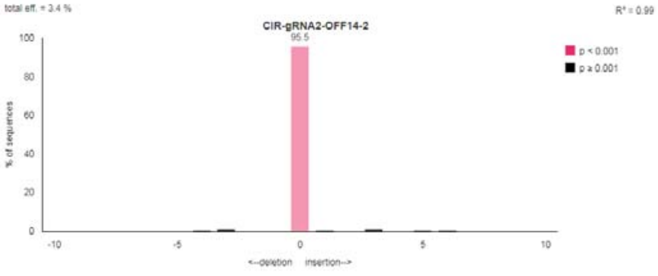
total eff. = 3.4 %
R^2 = 0.99
CIR-gRNA2-OFF14-2
95.5
p < 0.001
p ≥ 0.001
% of sequences
<--deletion insertion-->

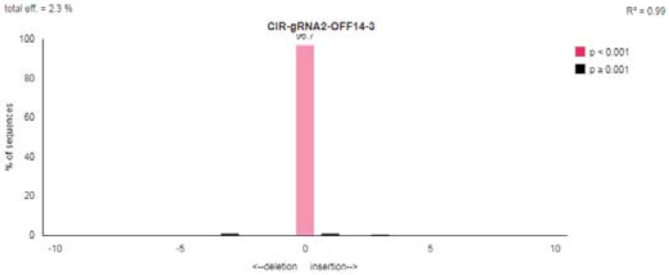
total eff. = 2.3 %
R^2 = 0.99
CIR-gRNA2-OFF14-3
p < 0.001
p ≥ 0.001
% of sequences
<--deletion insertion-->

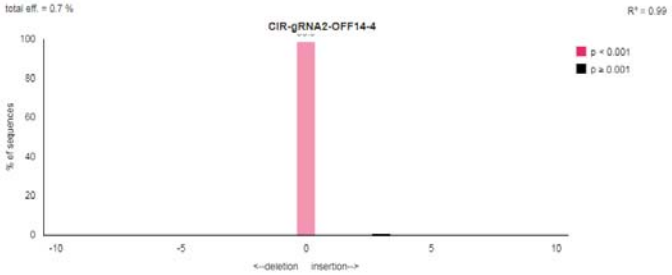
total eff. = 0.7 %
R^2 = 0.99
CIR-gRNA2-OFF14-4
p < 0.001
p ≥ 0.001
% of sequences
<--deletion insertion-->

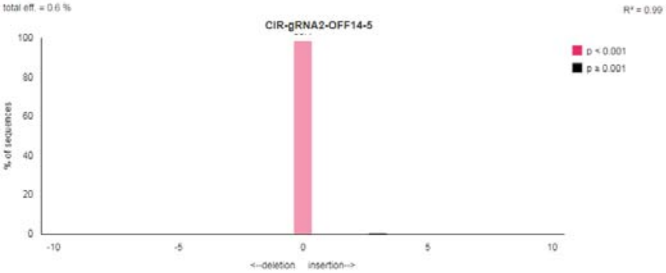
total eff. = 0.6 %
R^2 = 0.99
CIR-gRNA2-OFF14-5
p < 0.001
p ≥ 0.001
% of sequences
<--deletion insertion-->

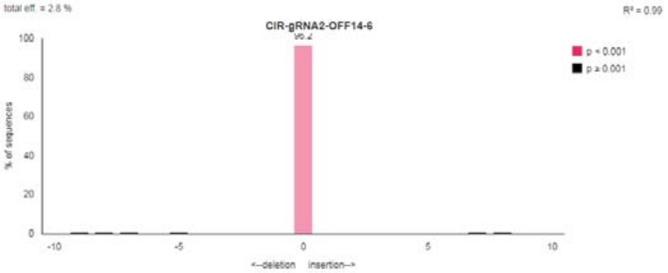
total eff. = 2.8 %
R^2 = 0.99
CIR-gRNA2-OFF14-6
p < 0.001
p ≥ 0.001
% of sequences
<--deletion insertion-->

gRNA2-OFF15

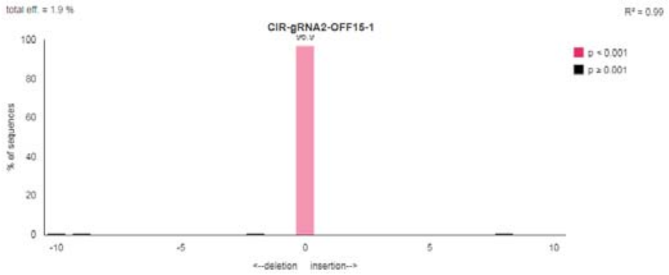
total eff. = 1.9 %
R^2 = 0.99
CIR-gRNA2-OFF15-1
p < 0.001
p ≥ 0.001
% of sequences
<--deletion insertion-->

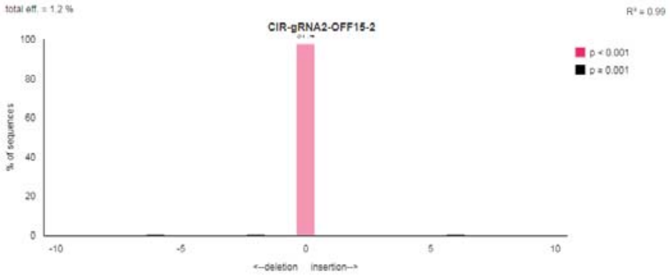
total eff. = 1.2 %
R^2 = 0.99
CIR-gRNA2-OFF15-2
p < 0.001
p ≥ 0.001
% of sequences
<--deletion insertion-->

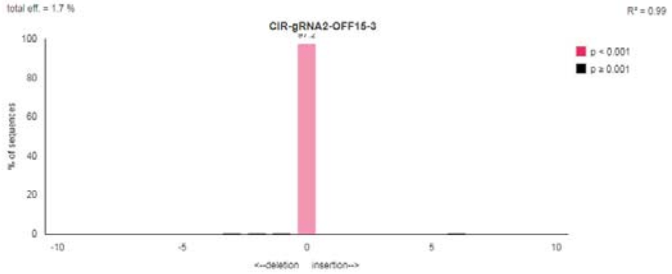
total eff. = 1.7 %
R^2 = 0.99
CIR-gRNA2-OFF15-3
p < 0.001
p ≥ 0.001
% of sequences
<--deletion insertion-->

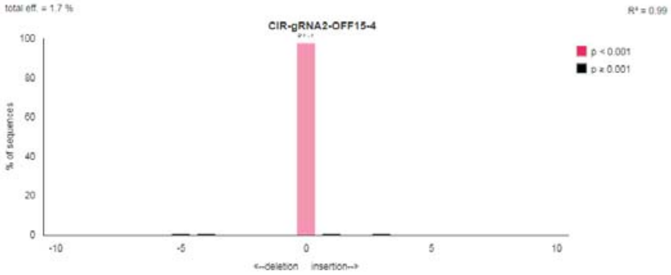
total eff. = 1.7 %
R^2 = 0.99
CIR-gRNA2-OFF15-4
p < 0.001
p ≥ 0.001
% of sequences
<--deletion insertion-->

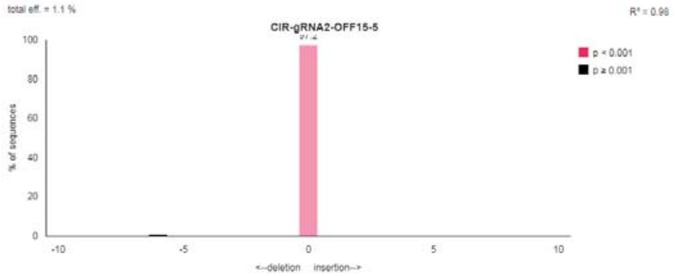
total eff. = 1.1 %
R^2 = 0.98
CIR-gRNA2-OFF15-5
p < 0.001
p ≥ 0.001
% of sequences
<--deletion insertion-->

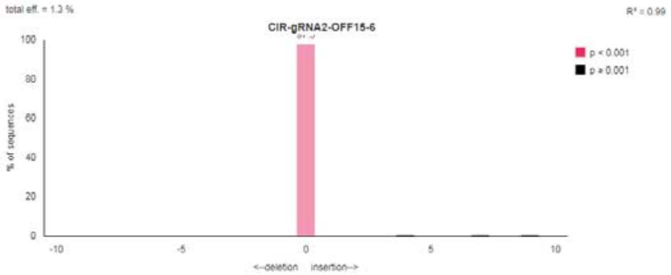
total eff. = 1.3 %
R^2 = 0.99
CIR-gRNA2-OFF15-6
p < 0.001
p ≥ 0.001
% of sequences
<--deletion insertion-->

gRNA2-OFF16

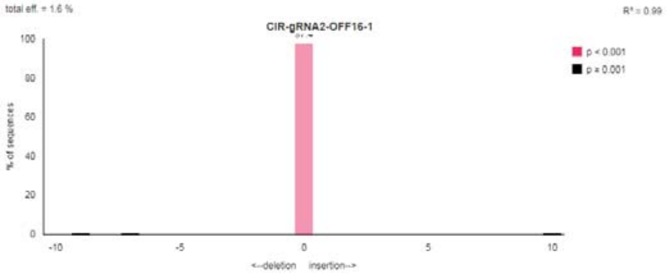
total eff. = 1.6 %
R² = 0.99
CIR-gRNA2-OFF16-1
p < 0.001
p ≥ 0.001
% of sequences
<--deletion insertion-->

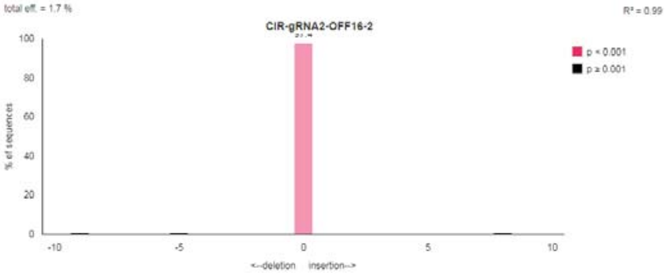
total eff. = 1.7 %
R² = 0.99
CIR-gRNA2-OFF16-2
p < 0.001
p ≥ 0.001
% of sequences
<--deletion insertion-->

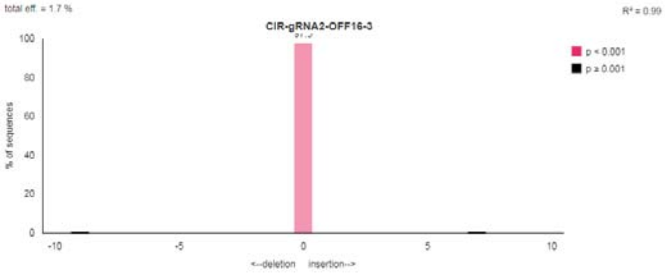
total eff. = 1.7 %
R² = 0.99
CIR-gRNA2-OFF16-3
p < 0.001
p ≥ 0.001
% of sequences
<--deletion insertion-->

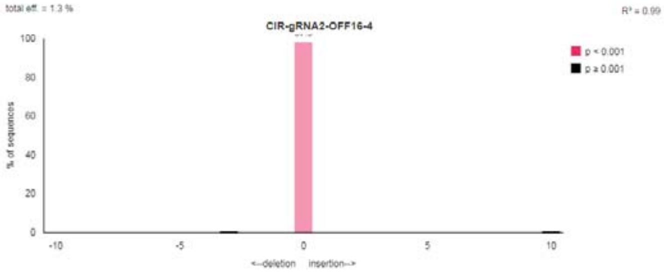
total eff. = 1.3 %
R² = 0.99
CIR-gRNA2-OFF16-4
p < 0.001
p ≥ 0.001
% of sequences
<--deletion insertion-->

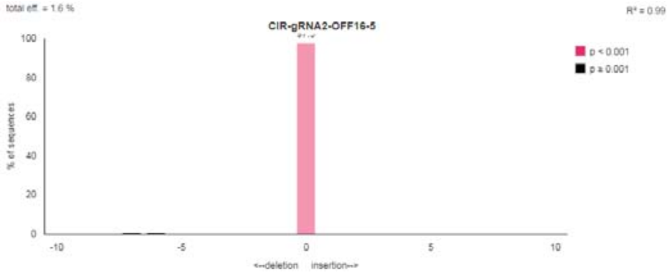
total eff. = 1.6 %
R² = 0.99
CIR-gRNA2-OFF16-5
p < 0.001
p ≥ 0.001
% of sequences
<--deletion insertion-->

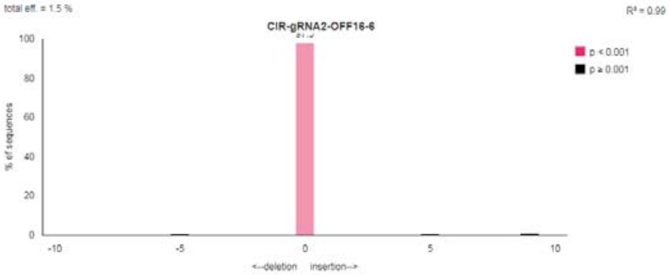
total eff. = 1.5 %
R² = 0.99
CIR-gRNA2-OFF16-6
p < 0.001
p ≥ 0.001
% of sequences
<--deletion insertion-->

gRNA2-OFF17

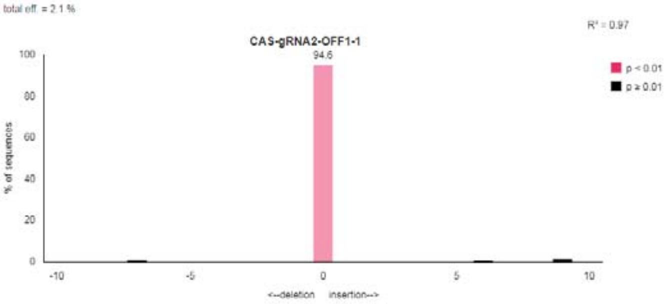
total eff. = 2.1 %
R² = 0.97
CAS-gRNA2-OFF1-1
94.6
p < 0.01
p ≥ 0.01
% of sequences
<--deletion insertion-->

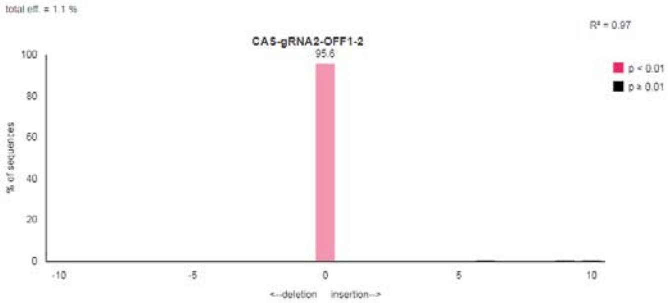
total eff. = 1.1 %
R² = 0.97
CAS-gRNA2-OFF1-2
95.6
p < 0.01
p ≥ 0.01
% of sequences
<--deletion insertion-->

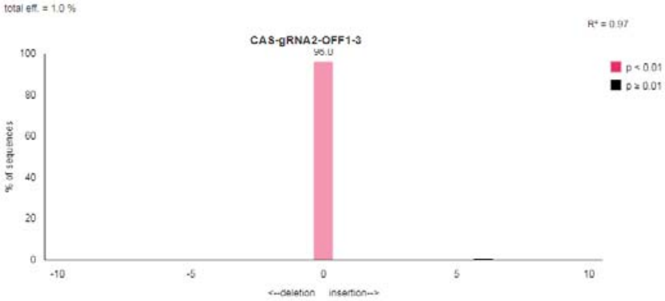
total eff. = 1.0 %
R² = 0.97
CAS-gRNA2-OFF1-3
p < 0.01
p ≥ 0.01
% of sequences
<--deletion insertion-->

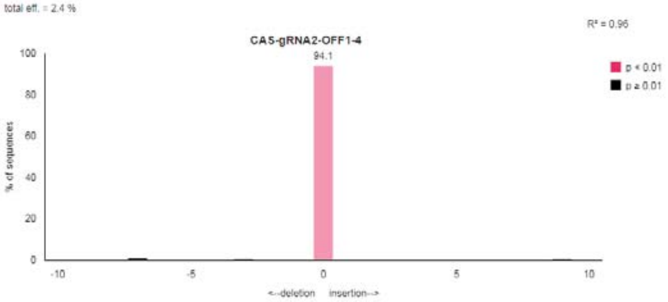
total eff. = 2.4 %
R² = 0.96
CAS-gRNA2-OFF1-4
94.1
p < 0.01
p ≥ 0.01
% of sequences
<--deletion insertion-->

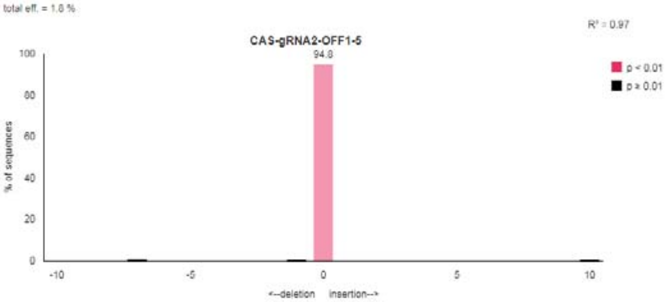
total eff. = 1.8 %
R² = 0.97
CAS-gRNA2-OFF1-5
94.8
p < 0.01
p ≥ 0.01
% of sequences
<--deletion insertion-->

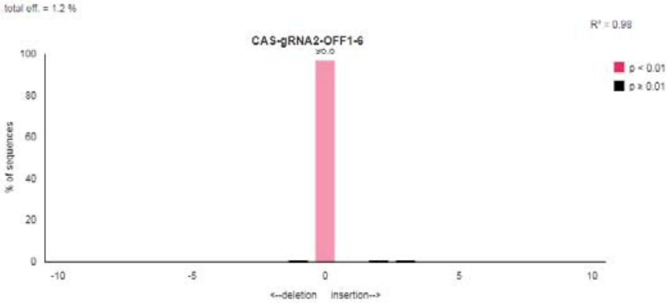
total eff. = 1.2 %
R² = 0.98
CAS-gRNA2-OFF1-6
p < 0.01
p ≥ 0.01
% of sequences
<--deletion insertion-->

gRNA2-OFF18

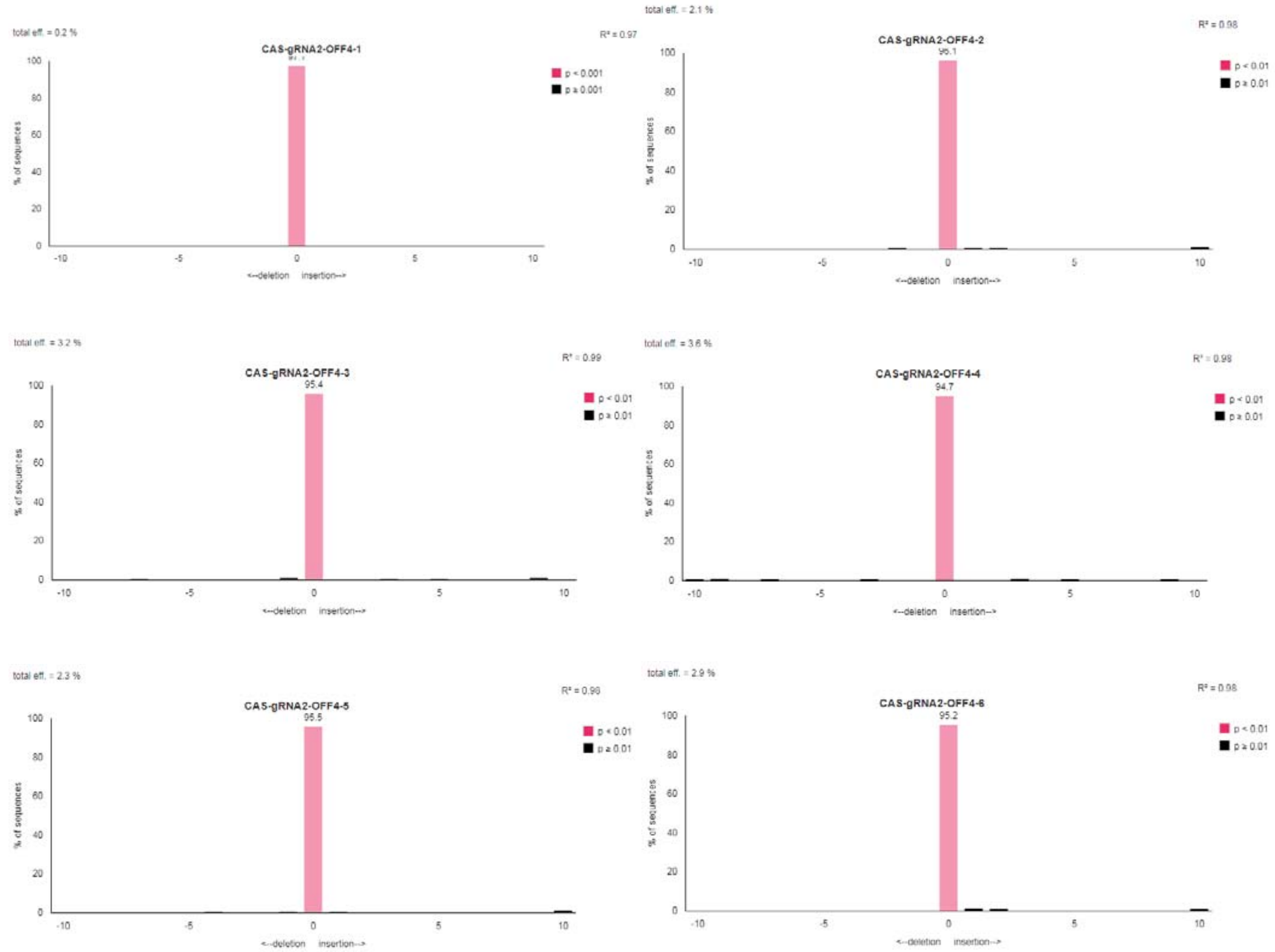

total eff. = 0.2 %
R² = 0.97
CAS-gRNA2-OFF4-1
p < 0.001
p ≥ 0.001
% of sequences
<--deletion insertion-->
total eff. = 2.1 %
R² = 0.98
CAS-gRNA2-OFF4-2
96.1
p < 0.01
p ≥ 0.01
% of sequences
<--deletion insertion-->
total eff. = 3.2 %
R² = 0.99
CAS-gRNA2-OFF4-3
95.4
p < 0.01
p ≥ 0.01
% of sequences
<--deletion insertion-->
total eff. = 3.6 %
R² = 0.98
CAS-gRNA2-OFF4-4
94.7
p < 0.01
p ≥ 0.01
% of sequences
<--deletion insertion-->
total eff. = 2.3 %
R² = 0.98
CAS-gRNA2-OFF4-5
95.5
p < 0.01
p ≥ 0.01
% of sequences
<--deletion insertion-->
total eff. = 2.9 %
R² = 0.98
CAS-gRNA2-OFF4-6
95.2
p < 0.01
p ≥ 0.01
% of sequences
<--deletion insertion-->

gRNA2-OFF19

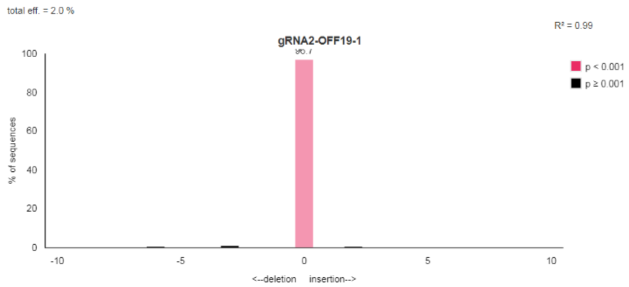

total eff. = 2.0 %
R² = 0.99
gRNA2-OFF19-1
p < 0.001
p ≥ 0.001
% of sequences
<--deletion insertion-->

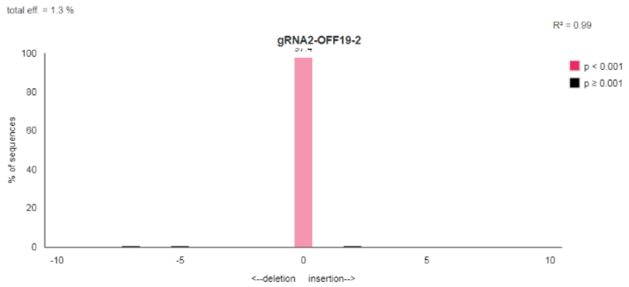

total eff. = 1.3 %
R² = 0.99
gRNA2-OFF19-2
p < 0.001
p ≥ 0.001
% of sequences
<--deletion insertion-->

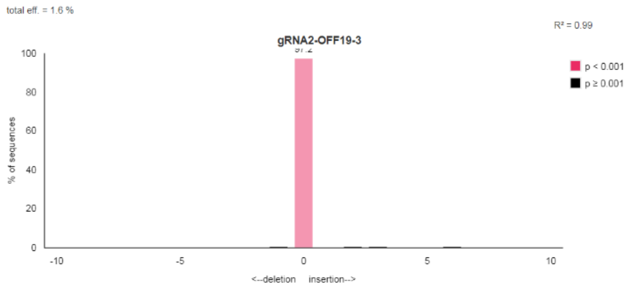

total eff. = 1.6 %
R² = 0.99
gRNA2-OFF19-3
p < 0.001
p ≥ 0.001
% of sequences
<--deletion insertion-->

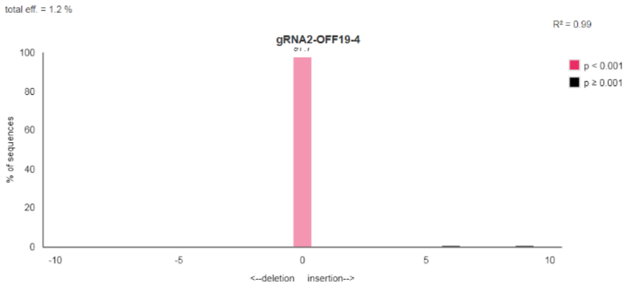

total eff. = 1.2 %
R² = 0.99
gRNA2-OFF19-4
p < 0.001
p ≥ 0.001
% of sequences
<--deletion insertion-->

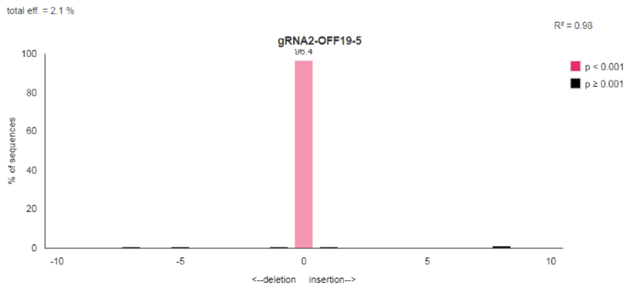

total eff. = 2.1 %
R² = 0.98
gRNA2-OFF19-5
96.4
p < 0.001
p ≥ 0.001
% of sequences
<--deletion insertion-->

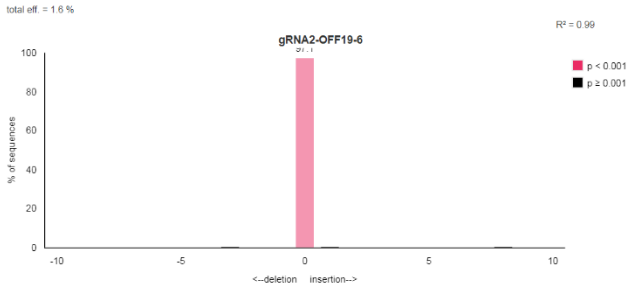

total eff. = 1.6 %
R² = 0.99
gRNA2-OFF19-6
p < 0.001
p ≥ 0.001
% of sequences
<--deletion insertion-->

gRNA2-OFF20

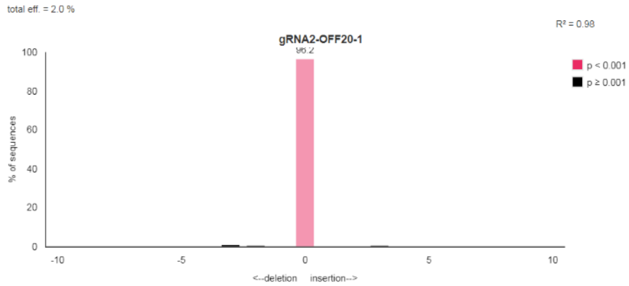
total eff. = 2.0 %
R² = 0.98
gRNA2-OFF20-1
96.2
p < 0.001
p ≥ 0.001
% of sequences
<--deletion insertion-->

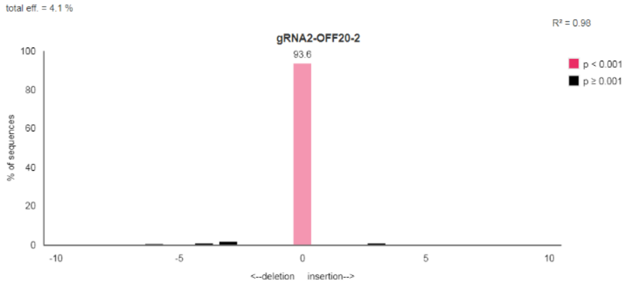
total eff. = 4.1 %
R² = 0.98
gRNA2-OFF20-2
93.6
p < 0.001
p ≥ 0.001
% of sequences
<--deletion insertion-->

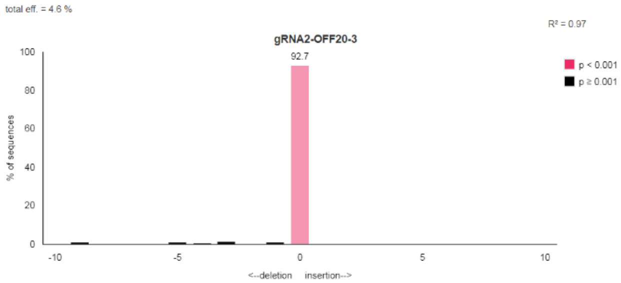
total eff. = 4.6 %
R² = 0.97
gRNA2-OFF20-3
92.7
p < 0.001
p ≥ 0.001
% of sequences
<--deletion insertion-->

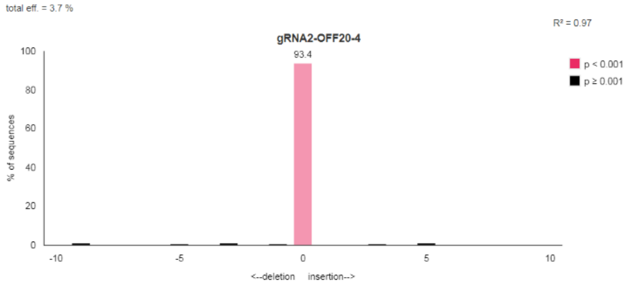
total eff. = 3.7 %
R² = 0.97
gRNA2-OFF20-4
93.4
p < 0.001
p ≥ 0.001
% of sequences
<--deletion insertion-->

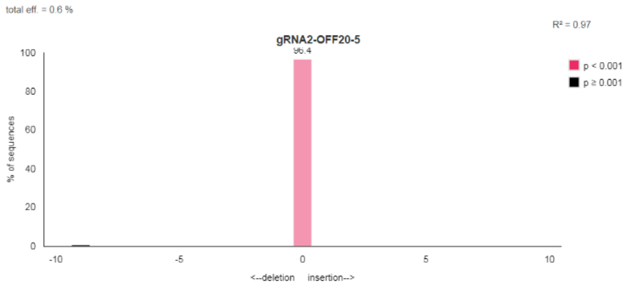
total eff. = 0.6 %
R² = 0.97
gRNA2-OFF20-5
96.4
p < 0.001
p ≥ 0.001
% of sequences
<--deletion insertion-->

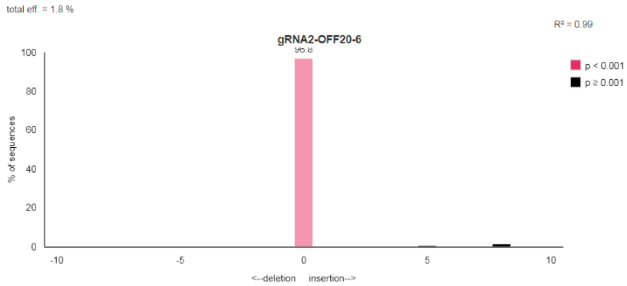
total eff. = 1.8 %
R² = 0.99
gRNA2-OFF20-6
96.8
p < 0.001
p ≥ 0.001
% of sequences
<--deletion insertion-->
